# Manganese(II) Complexes with Non-Steroidal Anti-Inflammatory Drugs: Structure and Biological Activity

**DOI:** 10.3390/ijms252413457

**Published:** 2024-12-16

**Authors:** Filitsa Dimiza, Antonios G. Hatzidimitriou, George Psomas

**Affiliations:** Department of General and Inorganic Chemistry, Faculty of Chemistry, Aristotle University of Thessaloniki, GR-54124 Thessaloniki, Greece

**Keywords:** NSAIDs, manganese(II) complexes, DNA interaction, affinity for albumins, antioxidant activity

## Abstract

Nine manganese(II) complexes with a series of non-steroidal anti-inflammatory drugs (namely sodium diclofenac, diflunisal, flufenamic acid, sodium meclofenamate, mefenamic acid, and tolfenamic acid) were prepared in the presence of diverse nitrogen donors, i.e., pyridine, 1,10–phenanthroline, 2,2′–bipyridine and neocuproine, as co-ligands and were characterized with spectroscopic techniques and single-crystal X-ray crystallography. The biological profile of the resultant complexes was investigated regarding their antioxidant potency and their interaction with DNA and serum albumins. The complexes interact with calf–thymus DNA in an intercalative mode and bind tightly and reversibly to human and bovine serum albumins studied. In order to assess the antioxidant activity of the Mn(II) complexes, their ability to scavenge 2,2′–azinobis(3–ethylbenzothiazoline–6–sulfonic acid) free radicals was monitored.

## 1. Introduction

Manganese is an important biometal not only because of its presence in the active center of many vital redox enzymes (oxygen-evolving center, superoxide dismutase, and catalase) but also due to its involvement in glucose metabolism, energy production, neuronal health, synthesis of cholesterol and fatty acids and protein digestion [1,2,3,4]. Manganese is also a cofactor in the synthesis and/or activation of diverse enzymes such as hydrolases, isomerases, transferases [5], and glutamine synthetase [6]. Despite its extended biological role, only two manganese compounds are used as chemotherapeutics, i.e., SC–52608 as an anticancer agent and Teslascan as a MRI contrast agent [7]. Nevertheless, the quest for medicinal inorganic chemistry regarding metallodrugs has included manganese complexes showing anticancer [8,9], antioxidant [10,11,12], antimicrobial [13,14], and antifungal [15] efficacy.

Non-steroidal anti-inflammatory drugs (NSAIDs) constitute a large category of drugs used for the treatment of symptoms, pain, and inflammation originating from injuries and/or diseases [16,17]. In addition, NSAIDs can enhance the activity of anticancer drugs [18] and exhibit activity against diverse cell lines following apoptotic [19] or radical scavenging mechanisms [20]. On the basis of their structural characteristic groups, NSAIDs include derivatives of anthranilic acid, phenylalkanoic acid, and salicylic acid, as well as furanones, oxicams, and sulfonamides [17].

**Figure 1:**
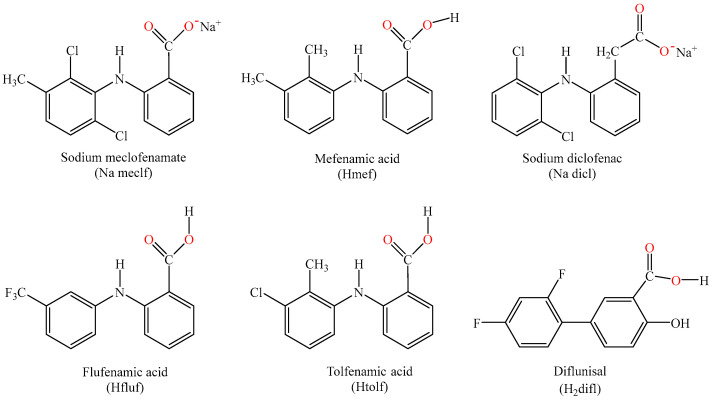
The formula of the NSAIDs used in the present study: sodium meclofenamate (Na meclf), mefenamic acid (Hmef), sodium diclofenac (Na dicl), flufenamic acid (Hfluf), tolfenamic acid (Htolf), and diflunisal (H_2_difl).

The NSAIDs used in the current research are the fenamates sodium meclofenamate (Na meclf), mefenamic acid (Hmef), flufenamic acid (Hfluf), and tolfenamic acid (Htolf), the salicylate derivative diflunisal (H_2_difl), and the phenylalkanoic sodium diclofenac (Na dicl) (Figure 1). As typical NSAIDs, they are commonly used to treat or alleviate various painful symptoms originating from inflammations such as migraines and acute (Hmef) [21] or moderate dysmenorrhea pain (Hfluf) [22], osteoarthritis and painful musculoskeletal disorders (Na meclf) [23], rheumatoid arthritis (Na dicl) [24], oral surgeries [25] and transthyretin amyloidosis cardiomyopathy [26] (H_2_difl), as well as in veterinary cases (Htolf) [27]. As a result of their analgetic antipyretic efficacy, most of the NSAIDs have been proposed for the treatment of COVID-19 and symptoms [28,29].

The significance of transition metal ions in biological systems (known as trace elements) has been well established for many years. In this context, bioinorganic chemists orientated their research toward the development of novel coordination compounds with enhanced or differentiated biological activity in comparison to the corresponding free ligands [30]. Other than the biological activity attributed to metal ions, their contribution to the coordination compounds is related to redox properties, unique coordination environments, Lewis acidic character, or charge variation possibilities [31,32]. Within this context, there are a lot of reports in the literature concerning the structural characterization and the biological activity of metal complexes bearing the aforementioned NSAIDs as ligands [16,17,33], including a series of Mn(II/III) [10,11,12,34,35,36,37,38], Fe(III) [39,40], Co(II) [41,42,43,44], Ni(II) [45,46], Cu(II) [47,48], Zn(II) [49,50], Ag(I) [51,52,53], Sn(IV) [54], Au(I) [55], and lanthanides(III) [56] complexes which presented enhanced biological behavior.

**Figure 2:**
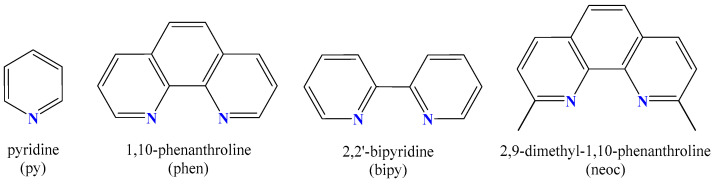
The formula of nitrogen donors used in the present study: pyridine (py), 1,10–phenanthroline (phen), 2,2′–bipyridine (bipy), and 2,9–dimethyl–1,10–phenanthroline (neoc).

According to the improved biological activity of metal–NSAID complexes and biological significance of manganese, and in continuation to our research projects concerning Mn(II)–NSAID complexes and the impact of nitrogen donor co-ligands on structural properties and biological activity [10,11,12,34,35], nine Mn(II) complexes with Na meclo, Hmef, Htolf, Hfluf, H_2_difl, and Na dicl in the presence or absence of the *N*–donor pyridine (py) or *N,N*′–donors 2,2′–bipyridine (bipy), 2,9–dimethyl–1,10–phenanthroline (neoc) and 1,10–phenanthroline (phen) (Figure 2) have synthesized and characterized. The resultant complexes **1**–**9** were characterized using spectroscopic (FT-IR and UV-vis) and single-crystal X-ray crystallography. The study of the biological profile of complexes **1**–**9** is focused on the following: (i) the antioxidant activity by determining the ability to scavenge 2,2′–azinobis–(3–ethylbenzothiazoline–6–sulfonic acid) (ABTS) free radicals; (ii) the interaction with calf–thymus (CT) DNA monitored with viscosity measurements, UV-vis spectroscopy, and through competition with the intercalation marker ethidium bromide (EB) by fluorescence emission spectroscopy; and (iii) the affinity for bovine serum albumin (BSA) and human serum albumin (HSA), investigated with fluorescence emission spectroscopy.

## 2. Results and Discussion

### 2.1. Synthesis and Characterization of the Complexes

The complexes were synthesized via the aerobic reaction of a methanolic solution of the corresponding salt of the NSAID with MnCl_2_·4H_2_O and pyridine in a 1:2:2 Mn^2+^: NSAID^−1^: py ratio for complexes **1**–**2**, while the use of corresponding *N,N′*–donor co-ligand (phen bipy, neoc) in a 1:2:1 Mn^2+^: NSAID^−1^: (*N,N′*–donor) ratio afforded complexes **3**–**9**.

All complexes **1**–**9** were characterized with IR and UV-vis spectroscopy and X-ray crystallography. The complexes proved stable in air, were soluble in DMSO and DMF (for complexes **1**–**4**), and were insoluble in H_2_O and most organic solvents. According to the values of molar conductivity (Λ_M_ = 6–10 mho∙cm^2^∙mol^–1^ for 1 mM DMSO solution), the complexes are non-electrolytes in DMSO solution (e.g., for a 1:1 electrolyte, a Λ_M_ value higher than 70 S cm^2^ mol^−1^ would be expected) [57] and thus keep their integrity in solution since they do not dissociate.

IR spectroscopy was used to confirm the existence of the NSAID ligands and nitrogen donor co-ligands in complexes **1**–**9**. In the IR spectra of the complexes (Figures S1–S4), two intense bands assignable to the antisymmetric (*v*_asym_(COO)) and the symmetric (*v*_sym_(COO)) stretching vibrations of the NSAID–carboxylato group were observed in the regions 1577–1613 cm^−1^ and 1376–1426 cm^−1^, respectively. The value of the parameter Δ*v*(COO) [determined as the difference *ν*_asym_(COO)–*ν*_sym_(COO)], when compared to the corresponding Δ*v*(COO) value of the corresponding salt, may indicate the coordination mode of the carboxylato group of a ligand. The values of Δ*v*(COO) for complexes **1**–**4** were calculated in the range of 192–197 cm^−1^, revealing a monodentate coordination mode (since they are higher than that of the corresponding salt) [58]. Similarly, for complexes **5**–**8**, the calculated Δ*v*(COO) values (= 175–190 cm^–1^) were lower than the corresponding Δ*v*(COO) value of the NSAID salt, leading to the conclusion of a bidentate chelating mode [58]. For complex **9**, two Δ*v*(COO) values were calculated (=182 cm^−1^ and 216 cm^−1^), revealing a bidentate chelating and a monodentate coordination mode, respectively [59]. Furthermore, the existence of the nitrogen donors was confirmed from the appearance of the bands assigned to characteristic out-of-plane ρ(C–H) vibrations in the “fingerprint” region 695–765 cm^−1^: i.e., 699–701 cm^−1^ for ρ(C–H)_py_ in complexes **1** and **2**, 729 cm^−1^ for ρ(C–H)_phen_ in complex **3**, 761 cm^−1^ for ρ(C–H)_bipy_ in **4**, 729–732 cm^−1^ for ρ(C–H)_neoc_ in **5**–**9** [58]. Such features regarding the coordination of the carboxylato group of the NSAID ligands and the presence of the nitrogen donor co-ligands are in good agreement with the crystal structures of the complexes determined and discussed in Section 3.2.

The electronic (UV-vis) spectra of complexes **1**–**9** were recorded as nujol mull and in DMSO solution and were found similar, suggesting that the complexes retain their structure in solution (the pharmacophore complex consisting of Mn(II), the NSAID ligands and the nitrogen donor co-ligand(s) remains stable). In the UV region of the spectra, the observed bands in the range of 270–368 nm (ε = 1700–25,000 M^−1^cm^−1^) are assigned to intra-ligand transitions.

### 2.2. Structure of the Complexes

The molecular structures of all complexes **1**–**9** were determined with single-crystal X-ray crystallography. All the complexes are mononuclear. All crystallographic experimental data are summarized in Tables S1–S5.

#### 2.2.1. Structure of Complexes **1** and **2**

**Figure 3:**
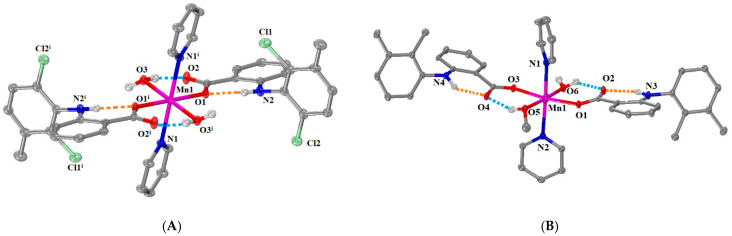
Molecular structure of (**A**) complex **1** (symmetry code: (i) −*x*+1, −*y*+1, −*z*+1) and (**B**) complex **2**. Methyl and aromatic hydrogen atoms and solvate molecules are omitted for clarity. Hydrogen bonds are given in (blue and orange for intramolecular and intraligand H bonds, respectively) dotted lines.

Complex **1** ([Μn(meclf–O)_2_(py)_2_(H_2_O)_2_]∙2py) and complex **2** ([Μn(mef–O)_2_(py)_2_(H_2_O)(MeOH)]∙1.5py), albeit of the same general formula [Mn(NSAID–O)_2_(py)_2_(O donor)_2_], have some differences which arise from the differentiation of the O–donor co-ligands. Both complexes **1** and **2** crystallized in a triclinic crystal system and *P–*1 space group (Table S1). Their molecular structures are depicted in Figure 3, and the selected bond distances and angles are given in Tables S6 and S7. Solvate pyridine molecules are also present in the structures of both complexes, i.e., two pyridine solvate molecules per molecule in complex **1** and three pyridine solvate molecules per two adjacent molecules in complex **2**.

In both complexes, the deprotonated NSAID ligands (meclofenamato and mefenamato ligands) are monodentately coordinated to the Mn1 ion via a carboxylato oxygen. The coordination sphere of the six-coordinate Mn(II) ion is MnN_2_O_4_ revealing a distorted octahedral geometry around Mn1 and includes the carboxylato oxygens from two monodentate NSAID ligands, two nitrogen atoms from two pyridine ligands and two oxygen atoms originating from two O–donors, i.e., two O atoms from H_2_O ligands in centrosymmetric complex **1** or one aqua and one methanol oxygen atoms in complex **2**. For both complexes, the Mn1—O_carboxylato_ bond distances are the shortest ones (2.1554(19) Å in **1**, 2.1215(18) Å and 2.1501(19) Å in **2**) and the Mn1—N_pyridine_ are the longest ones (2.325(2) Å in **1**, 2.259(2) Å and 2.280(2) Å in **2**) in the coordination spheres of Mn1 ions (Tables S6 and S7). Similar structures concerning metal–NSAID complexes with pyridine co-ligands were reported for complexes [Mn(naproxen)_2_(py)_2_(H_2_O)_2_] [12], [Mn(mef)_2_(imidazole)_2_(EtOH)_2_] and [Mn(mef)_2_(2–methylimidazole)_2_(EtOH)_2_] [38].

Three types of hydrogen bonds (intraligand, intramolecular, and intermolecular) are formed and provide further stabilization of the structure. In complex **1**, the H bonds present are intraligand H bonds between the coordinated carboxylate oxygen atoms O1 and the imine hydrogen atoms H21 of meclofenamato ligands, intramolecular H bonds between non-coordinated carboxylate oxygen atoms O2 and the aqua hydrogen H242, and intermolecular H bonds between the second aqua hydrogen H32 and N3 of pyridine solvate molecule (Table S8). In complex **2**, the non-coordinated carboxylato oxygens O4 and O2 participate in an extended H bond network forming intraligand H bonds with imine hydrogen atoms H515 and H41 of the mefenamato ligands and intramolecular H bonds with aqua H62 and H51 atoms, while intermolecular H bonds between the aqua hydrogen H61 and N3 of pyridine solvate molecule are formed (Table S8).

#### 2.2.2. Structure of Complexes **3** and **4**

**Figure 4:**
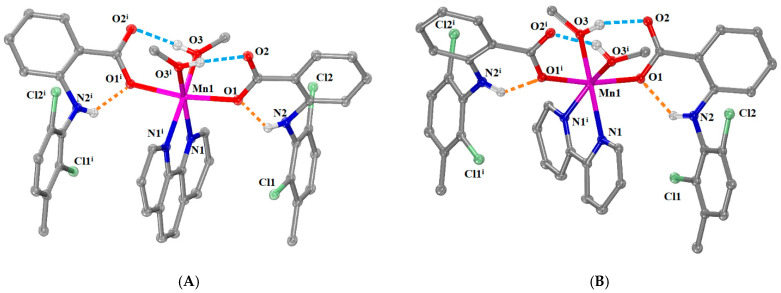
Molecular structure of (**A**) [Μn(meclf–O)_2_(phen)(MeOH)_2_] (complex **3**) (symmetry code: (i) −*x*+1, *y*, −*z*+1/2) and (**B**) [Mn(meclf–O)_2_(bipy)(MeOH)_2_] (complex **4**) (symmetry code: (i) −*x*+1, *y*, −*z*+1/2). Methyl and aromatic hydrogen atoms are omitted for clarity. Hydrogen bonds are depicted in (blue and orange for intramolecular and intraligand H bonds, respectively) dotted lines.

[Μn(meclf–O)_2_(phen)(MeOH)_2_] (complex **3**) and [Mn(meclf–O)_2_(bipy)(MeOH)_2_] (complex **4**) have the same general formula [Mn(meclf–O)_2_(*N,N’*–donor)(MeOH)_2_] with different *N,N’*–donor co-ligands, i.e., phen for **3** and bipy for **4**. Both complexes **3** and **4** crystallized in a monoclinic crystal system and *C*2/*c* space group (Table S2). The molecular structures are given in Figure 4, and the selected bond distances and angles are cited in Table S9.

The complexes are mononuclear and contain two deprotonated monodentate meclofenamato, a bidentate *N,N’*–donor (phen for **3** and bipy for **4**), and two methanol ligands, which result in MnN_2_O_4_ coordination sphere and a distorted octahedral geometry around six-coordinated Mn1. The complexes process a two-fold axis of symmetry that passes through the Mn1 ion and the middle of the coordinated *N,N’*–donor co-ligand. In the structures of both complexes, the Mn1—O_carboxylato_ bond distances are the shortest ones (2.133(2) Å in **3**, and 2.1162 (16) Å in **4**) and the Mn1—N*_N,N’_*_–donor_ are the longest ones (2.259(3) Å in **3**, and 2.283(2) Å in **4**) in the coordination spheres of Mn1 ions (Table S9). The arrangement of the NSAID and *N,N’*–donor ligands was similar to that observed in the complex [Mn(mef–O)_2_(bipy)(MeOH)_2_] [12,36].

The presence of intraligand and intramolecular hydrogen bonds stabilizes both structures further. More specifically, the intraligand hydrogen bonds are developed between the imino hydrogens H21 and the coordinated oxygens O1 of the meclofenamato ligands, while the intramolecular H bonds are formed between the methanol hydrogens H214 (in **3**) or H204 (in **4**) and the non-coordinated oxygens O2 of the meclofenamato ligands (Table S8).

#### 2.2.3. Structure of Complexes **5**–**9**

Complexes **5**–**9** possess the same general formula [Mn(NSAID)_2_(neoc)] but have differences concerning the binding of NSAID ligands or crystallographic concerns.

In particular, complexes **5**–**8** have similar general formulas of the type [Mn(NSAID–O,O’)_2_(neoc)]. They are mononuclear and contain two deprotonated NSAID ligands coordinated to Mn(II) ion in a bidentate chelating fashion and a bidentate neoc ligand. Such arrangement of the ligands results in MnN_2_O_4_ coordination sphere. In all these complexes, the geometry around the Mn(II) ions can be described as distorted trigonal prismatic; one carboxylato oxygen atom from each NSAID ligand and a nitrogen atom from neoc ligand form the basal planes of the prism. A similar arrangement of NSAID and *N,N’*–donor ligands (i.e., asymmetric bidentate chelating coordination of the NSAID and bidentate chelating coordination of the *N,N’*–donor ligands) was reported in the Mn(II) complex [Mn(dicl–O,O’)_2_(2,2′–bipyridylamine)] [10].

**Figure 5:**
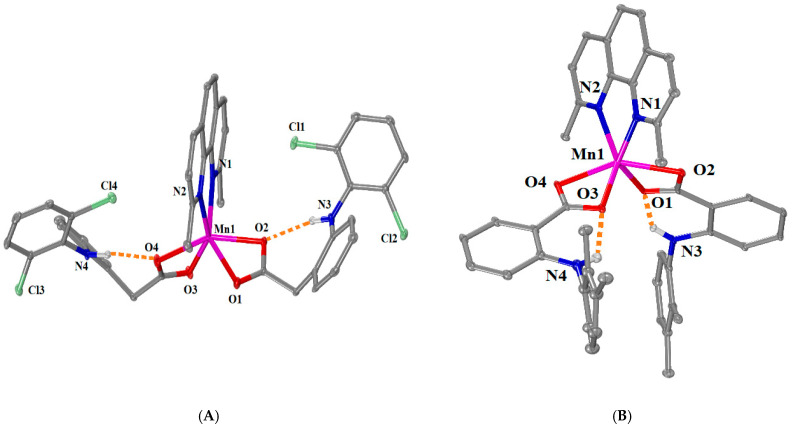
Molecular structure of (**A**) complex **5A** and (**B**) complex **6A**. Methyl and aromatic hydrogen atoms and solvate molecules are omitted for clarity. Intraligand hydrogen bonds are given in orange dotted lines.

Complex **5** ([Μn(dicl–O,O’)_2_(neoc)]∙0.25H_2_O) and complex **6** ([Μn(mef–O,O’)_2_(neoc)]∙1.5MeOH∙0.25 H_2_O) crystallized in the triclinic crystal system and *P–*1 space group (Table S3). The asymmetric unit of the complexes comprises two crystallographically independent neutral mononuclear Mn(II) complexes, notated as complex **A** and **B** (presented in Figure 5 and Figure S5, respectively), as well as methanol and/or water solvate molecules. Selected bond distances and angles are given in Tables S10 and S11. The bases of the trigonal prism are formed by O1, O4, and N1 and O2, O3, and N2 in complex **5A** and O5, O8, and N5 and O6, O7, and N6 in complex **5B** and have an angle of 16.2° and 19.2°, respectively. Similarly, the trigonal bases of the prism in complex **6A** are formed by O1, O4, and N2 and O2, O3, and N1 and by O5, O8, and N5 and O6, O7, and N6 in complex **6B**, forming an angle of 15.2° and 13.2°, respectively. Intraligand hydrogen bonds are developed between imine hydrogen atoms and coordinated carboxylato oxygens atoms of the diclofenac and mefenamato ligands in complexes **5** and **6**, respectively. Further stabilization of the structures of these complexes is provided by intermolecular hydrogen bonds between carboxylato oxygen atoms and methanol and/or water solvate molecules (Table S13).

**Figure 6:**
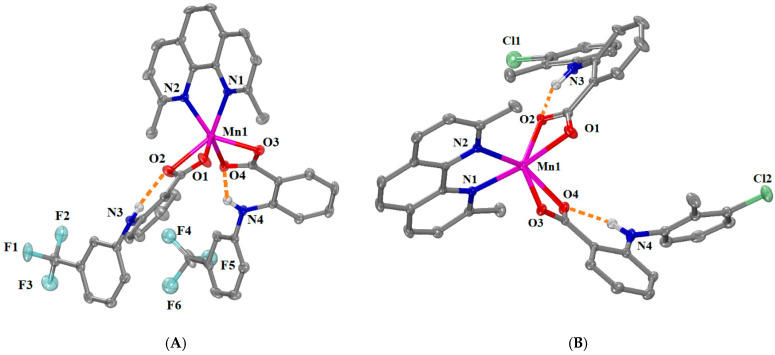
Molecular structure of (**A**) [Μn(fluf–O,O’)_2_(neoc)] (complex **7**) and (**B**) [Μn(tolf–O,O’)_2_(neoc)] (complex **8**). Methyl and aromatic hydrogen atoms are omitted for clarity. Intraligand hydrogen bonds are given in orange dotted lines.

Quite similar structures were determined for [Μn(fluf–O,O’)_2_(neoc)] (complex **7**) and [Μn(tolf–O,O’)_2_(neoc)] (complex **8**) with the difference that only one independent molecule was observed in the asymmetric unit. The structure of reported complex [Mn(flufenamato)_2_(neoc)] has been recently determined at 95 K [37] and is similar to that of complex **7** studied herein showing however differences in cell dimensions and cell volume as well as bond distances and angles. Complexes **7** and **8** crystallized in orthorhombic crystal system and *P*2_1_2_1_2_1_ space group, and monoclinic crystal system and *P*2_1_/*c* space group, respectively (Table S4). The molecular structures are given in Figure 6, and the selected bond distances and angles are shown in Table S12. The bases of the distorted trigonal prisms around Mn1 are formed in a similar way, i.e., by O1, O3, and N1 and O2, O4, and N2 with an angle of 11.8° in complex **7** and by O1, O3, and N2 and O2, O4, and N1 with and an angle of 13.2° in complex **8**. In addition, only intraligand H bonds are formed between imine hydrogen atoms and coordinated carboxylato oxygens atoms of the flufenamato and tolfenamato ligands in complexes **7** and **8**, respectively (Table S13).

**Figure 7:**
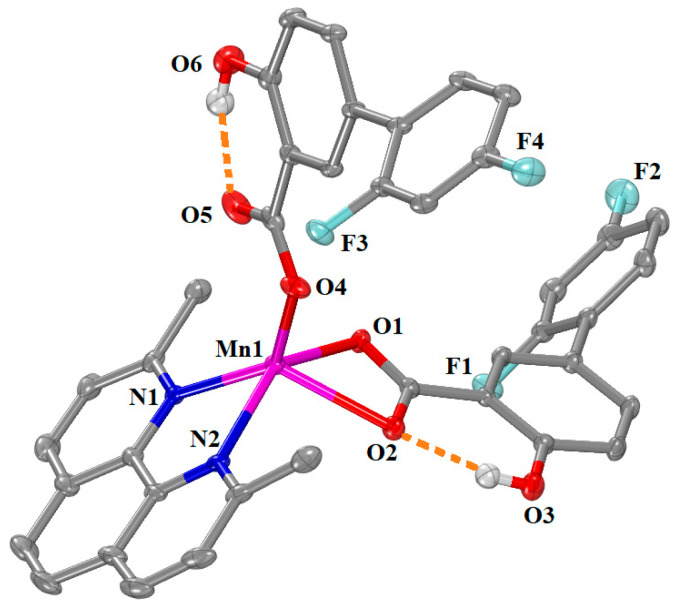
Molecular structure of complex **9**. Methyl and aromatic hydrogen atoms are omitted for clarity. Intraligand hydrogen bonds are given in orange dotted lines.

[Μn(Hdifl–O,O’)(Hdifl–O)(neoc)]·0.5MeOH (complex **9**) crystallized in triclinic crystal system and *P–*1 space group (Table S5). The molecular structure of complex **9** is depicted in Figure 7, and the selected bond distances and angles are summarized in Table S14. In this mononuclear complex, the two deprotonated diflunisal ligands behave in different coordination modes; one diflunisal ligand is bound to the Mn1 ion in an asymmetrical bidentate chelating mode via two carboxylate oxygen atoms O1 and O2 (Mn1—O1 = 2.1777(16) Å and Mn1—O2 = 2.3130(17) Å), while the other one is monodentately bound to the Mn1 ion via a carboxylate oxygen atom O4 (Mn1—O4 = 2.0357(17) Å; the non-coordinated O5 atom lies at 2.790 Å from Mn1). A combination of the monodentate and the bidentate chelating mode for a NSAID ligand was reported for the five-coordinate Mn(II)–NSAID complex [Mn(fenamato–O)(fenamato–O,O’)(neoc)] [37] and the six-coordinate Mn(II)–NSAID complexes [Mn(tolf–O)(tolf–O,O’)(phen)(H_2_O)] [11], [Mn(naproxen–O)(naproxen–O,O’)(phen)(H_2_O)] [12], and [Mn(mef–O)(mef–O,O’)(phen)(H_2_O)] [12,36].

The manganese atom is five-coordinate, and the other two vertices of its coordination polyhedron are occupied by two nitrogen atoms (N1 and N2 with Mn1—N1 = 2.2029(19) Å and Mn1—N2 = 2.2289(19) Å) from the neoc ligand. According to the value of the trigonality index τ_5_ = 0.105 (=(141.74°–135.46°)/60°, τ_5_ = (φ_1_ − φ_2_)/60°, φ_1_ and φ_2_ are the largest angles in the coordination sphere [60]; τ_5_ = 0 is found for a perfect square pyramid and τ_5_ = 1 for a perfect trigonal bipyramid)), the geometry around Mn1 ion can be described as a slightly distorted square pyramid with O1, N1, N2, and O4 forming the basal plane and O2 being at the apical of the pyramid. Intraligand hydrogen bonds are developed between the phenolic hydrogens H31 and H61 and carboxylato oxygens O2 and O5 of the Hdifl^–^ ligands (Table S13) providing additional stabilization of the structure.

### 2.3. Antioxidant Activity of the Complexes

Antioxidants are compounds that protect from or inhibit oxidation which is a chemical reaction that can produce free radicals. Antioxidants give up their own electrons to free radicals, inactivating their ability to damage biological molecules [61,62]. Diseases including cancer, inflammation, heart diseases, autoimmune diseases, aging, and Alzheimer’s disease are due to the presence of free radicals, and their treatment is the elimination of free radicals and oxidative stress with the use of an antioxidant agent [61,63]. NSAIDs act either through scavenging free radicals or by inhibiting their production [17,61]. Such antioxidant compounds may play a crucial role in the treatment of inflammation and open the paths for more effective pharmaceuticals [48].

The cationic radical of 2,2′–azino–bis–(3–ethylbenzothiazoline–6–sulfonic acid) (=ABTS) usually reacts with compounds that can act as antioxidants such as vitamin C [64]. ABTS is a commonly used agent to measure the antioxidant capacity of foods [65]. In general, ABTS is a marker of total radical scavenging efficacy, and the determination of its scavenging is related to the overall antioxidant capacity of a compound [66]. The determination of ABTS scavenging activity of the compounds is based on the discoloration of a dark green solution of the radical ABTS^•+^ resulting from the presence of the compounds under study. 6–Hydroxy–2,5,7,8–tetramethylchromane–2–carboxylic acid (trolox) is the most common reference compound for the evaluation of the ABTS scavenging ability, and the results concerning complexes **1**–**9** are summarized in Table 1.

Most complexes are highly active toward ABTS radicals and, in many cases, more active than the corresponding free NSAIDs. Especially, complexes **2**, **4**–**6**, and **8** (namely the Mn(II) complexes with mefenamato, tolfenamato, and diclofenac ligands) present significantly higher activity (ABTS% = 92.56 ± 0.74–99.63 ± 0.07%) than the reference compound trolox (ABTS% = 91.8 ± 0.17%) (Table 1). Complex **8** is the best ABTS scavenger (ABTS% = 99.63 ± 0.07%) among complexes **1**–**9** under study and other reported metal(II)–NSAID complexes [11,17,39,40,41,45,48].

### 2.4. Interaction of the Complexes with CT DNA

The investigation of the DNA affinity of the compounds is crucial for a variety of biomedical applications [50]. NSAIDs and their metal complexes may interact with DNA in different ways according to the structure and stability of the complexes and the nature of the corresponding ligands [67]. More specifically, the coordination compounds either interact covalently with DNA (covalently bound to nitrogens of DNA bases) or noncovalently (i.e., through intercalation, groove binding, or electrostatic interaction) and/or may induce cleavage of the DNA helix [50,68]. The interaction of complexes **1**–**9** with CT DNA was monitored with UV-vis spectroscopy and viscosity measurements and through competitive studies with EB monitored with fluorescence emission spectroscopy.

UV-vis spectroscopy is employed to obtain initial information concerning the affinity, interaction mode, and strength between complexes and DNA through the calculation of the corresponding DNA–binding constant (K_b_). For this purpose, titration studies using UV-vis spectroscopy were employed to monitor the alterations in the UV-vis spectra of a DNA solution in the presence of the complexes and vice versa.

Initially, the UV-vis spectra of a CT DNA solution in buffer were recorded in the presence of complexes **1**–**9** at diverse ratios of [compound]/[DNA] (= *r*). Upon addition of increasing amounts of the compounds, a slight decrease or increase of the absorbance (either hypochromism or hyperchromism) of the CT DNA band located at λ_max_ = 258–260 nm was observed (Figure S6), indicating the existence of an interaction.

On the reverse titrations, the electronic spectra of complexes **1**–**9** were recorded in the presence of increasing amounts of CT DNA solution, and a series of changes in λ_max_ and/or absorbance of the intraligand band(s) of the complexes were observed (Figure S7, Table 2). The changes in the UV region of the spectrum are an indication of the interaction mode, since hypochromism due to π→π* interactions may occur in the case of intercalative interaction, while hyperchromism occurs in the case of groove binding or electrostatic interaction. In parallel, the presence of bathochromism is attributed to a stabilization of the CT DNA double helix [69].

**Figure 8:**
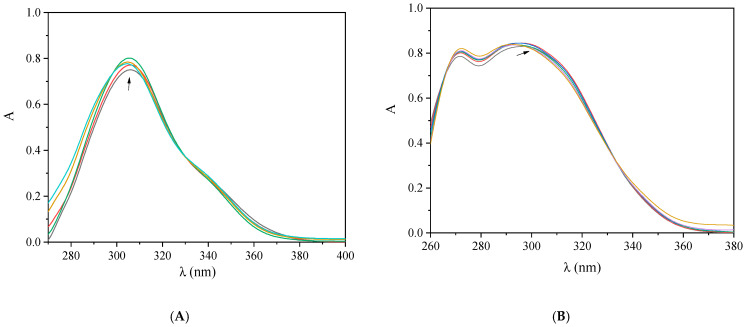
UV-vis spectra of a DMSO solution of (**A**) complex **2** (0.02 mM) and (**B**) complex **8** (0.02 mM) in the presence of increasing amounts of CT DNA. The colors in figures represent different concentrations of CT DNA and the arrows show the changes upon increasing amounts of CT DNA.

In the UV-vis spectra of complexes **1**–**9**, one or two intraligand bands were observed (Figure 8 and Figure S7). Upon incremental addition of a CT DNA solution, a slight decrease or increase in the absorbance of these bands was observed, which, in most cases, was accompanied by a bathochromism (Table 2), revealing a stabilization of the novel adduct (resulting from the interaction of the complexes with DNA). The overall spectroscopic changes reveal the existence of interaction, although the interaction mode cannot be firmly suggested [69]. In order to shed light on the DNA interaction mode of the complexes, DNA viscosity measurements and EB displacement studies were employed.

The DNA binding constants (K_b_) of complexes **1**–**9** (Table 2) were calculated with the Wolfe–Shimer equation (Equation (S1)) [70] and the corresponding plots [DNA]/(ε_A_ − ε_F_) *versus* [DNA] (Figure S8). Almost all complexes **1**–**9** presented higher affinity for DNA than the corresponding NSAIDs (Table 2), with complex **7** being the tightest DNA binder among the complexes (K_b_ = 1.10 (±0.02) × 10^6^ M^−1^). The K_b_ values of complexes **1**–**9** were of the same or higher magnitude than that of the classic intercalator EB (K_b_ = 1.23 (±0.07) × 10^5^ M^−1^) [71] and within the range reported for a series of metal(II)–NSAID complexes [11,41,45,48].

The viscosity measurement of a DNA solution is also used as a method of investigating and clarifying the interaction mode of the complexes with CT DNA. The viscosity of the DNA solution is sensitive to changes in the relative length of the DNA chain, constituting viscometry, which is a method useful to clarify the possible DNA interaction mode. In the case of classic intercalation, the relative DNA viscosity shows an increase, while in the case of nonclassical intercalation (groove binding or electrostatic interaction), it decreases slightly or remains unchanged [72].

**Figure 9:**
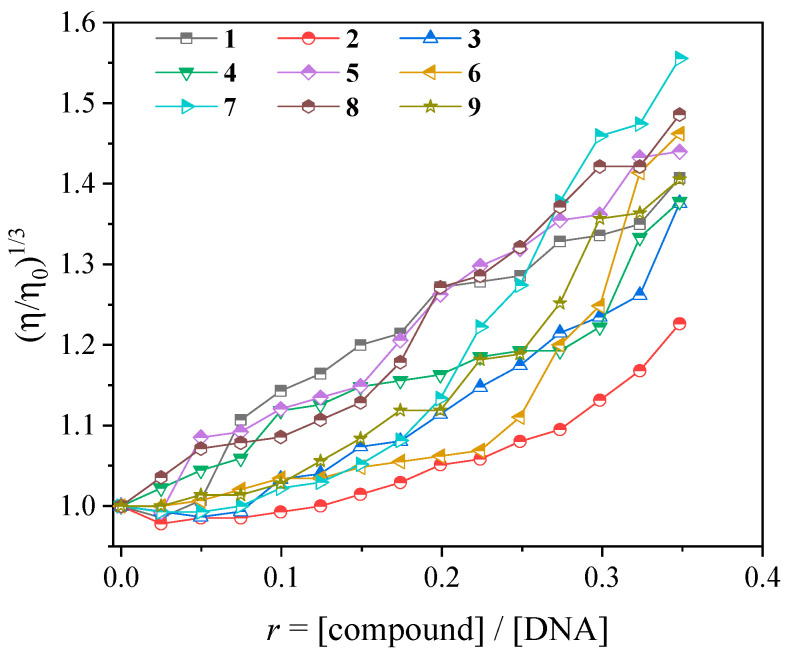
Relative viscosity of CT DNA (η/η_ο_)^1/3^ in buffer solution (150 mM NaCl and 15 mM trisodium citrate at pH 7.0) in the presence of complexes **1**–**9** at increasing amounts (*r* = [compound]/[DNA]).

The viscosity of a CT DNA solution (0.1 mM) was monitored upon the addition of increasing amounts of complexes **1**–**9** (up to *r* = [compound]/[DNA] = 0.36), and an increase in the relative DNA viscosity was observed (Figure 9). This increase in DNA viscosity originates from an increase in the relative DNA length, which is due to the increase of separation distances between DNA bases upon insertion of an intercalating molecule (i.e., complexes **1**–**9** in the present case) between the DNA bases and serves as evidence of possible intercalation between DNA and each complex [72].

EB is a typical DNA intercalator having fluorescent properties including the existence of a characteristic emission band with λ_max_ in the range of 592–594 nm when bound to DNA. The addition of an intercalating compound into an EB–DNA solution is expected to result in a displacement of EB from the EB–DNA adduct, and subsequently, a quenching of the intensity of the EB–DNA emission band will appear, while the presence of a non-intercalating compound will induce slight/negligible decrease in fluorescence emission [73]. The solutions of NSAIDs and their complexes **1**–**9** do not fluoresce in the absence or presence of CT DNA solution when excited at 540 nm; therefore, the alterations observed in the emission spectra of the EB–DNA adduct can be monitored to investigate the ability of the complexes to displace EB from the EB–DNA complex [73].

**Figure 10:**
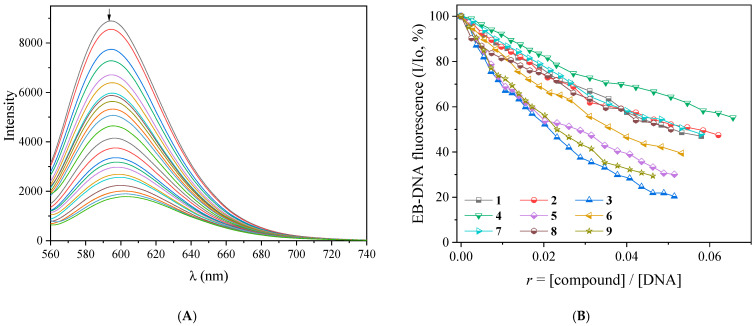
(**A**) Fluorescence emission spectra (λ_excitation_ = 540 nm) for EB–DNA ([EB] = 40 μM, [DNA] = 45 μM) in buffer solution (150 mM NaCl and 15 mM trisodium citrate at pH 7.0) in the absence and presence of increasing amounts of complex **3**. The colors in the figure represent different concentrations of the complex and the arrow shows the changes in intensity upon increasing amounts of **3**. (**B**) Plot of relative EB–DNA fluorescence intensity at λ_emission_ = 592 nm (I/Io, %) versus r (*r* = [compound]/[DNA]) in the presence of complexes **1**–**9** (up to 47.0% of the initial EB–DNA fluorescence emission intensity for **1**, 47.4% for **2**, 20.4% for **3**, 55.2% for **4**, 30.1% for **5**, 39.4% for **6**, 47.9% for **7**, 50.0% for **8**, and 29.4% for **9**).

The EB–DNA adduct was prepared from the 1 h pre-treatment of a solution containing EB ([EB] = 40 μM) and CT DNA ([DNA] = 45 μM). The fluorescence emission spectra of the EB–DNA adduct were recorded in the presence of incremental additions of complexes **1**–**9** (the effect of complex **3** is shown in Figure 10A). For all complexes, **1**–**9** (Figure S9), the emission of the EB–DNA band with λ_max_ in the range of 592–594 nm presented a significant decrease up to 79.6% of the initial EB–DNA fluorescence (Figure 10B and Table 3). Such high quenching obviously originates from the displacement of EB, which is due to the complexes and reveals their preference for the DNA intercalation sites [74].

The Stern–Volmer (K_sv_) and quenching (k_q_) constants (Table 3) were calculated with the Stern–Volmer equation (Equation (S2)) [73] (R~0.99 in the Stern–Volmer plots (Figure S10)) and Equation (S3) (the fluorescence lifetime of the EB–DNA (τ_o_) is equal to 23 ns [75]), respectively, with complex **3** exhibiting the highest K_sv_ and k_q_ constants (K_sv_ = 1.18 (±0.32) × 10^6^ M^–1^ and k_q_ = 5.15 (±0.14) × 10^13^ M^–1^s^–1^) among the complexes under study. The derived k_q_ values are much higher than the value of 10^10^ M^−1^s^−1^, indicating the presence of a static quenching mechanism [73], which may further confirm the formation of a new adduct between CT DNA and each complex. The K_SV_ and k_q_ values determined for complexes **1**–**9** are in the range found for other metal–NSAID complexes [11,39,40,41,45,48].

### 2.5. Interaction of the Complexes with Albumins

Serum albumin (SA) is the predominant protein in blood and among the most important ones in the body’s circulatory system [76]. Albumin is related to the transportation of drugs, fatty acids, organic substances, metabolites, and metal compounds toward their biological targets through the bloodstream to cells and tissues [77,78]. The binding to such proteins can lead to alterations of the biological properties of the original drug or reveal novel paths for transportation. A lot of previous studies have reported the dependence of the pharmacological and pharmacokinetic properties of drugs on their interaction with albumin, which is the key carrier protein present in blood plasma [79]. Solutions of albumins HSA and BSA, when excited at 295 nm, show an intense fluorescence emission band in the range of 340–350 nm arising from the tryptophan residues (i.e., tryptophan at position 214 in HSA and tryptophans −134 and −212 for its homolog BSA) [73]. The interaction of complexes **1**–**9** with the SAs was evaluated by monitoring the quenching of the tryptophan fluorescence emission band upon the addition of incremental amounts of the complexes.

**Figure 11:**
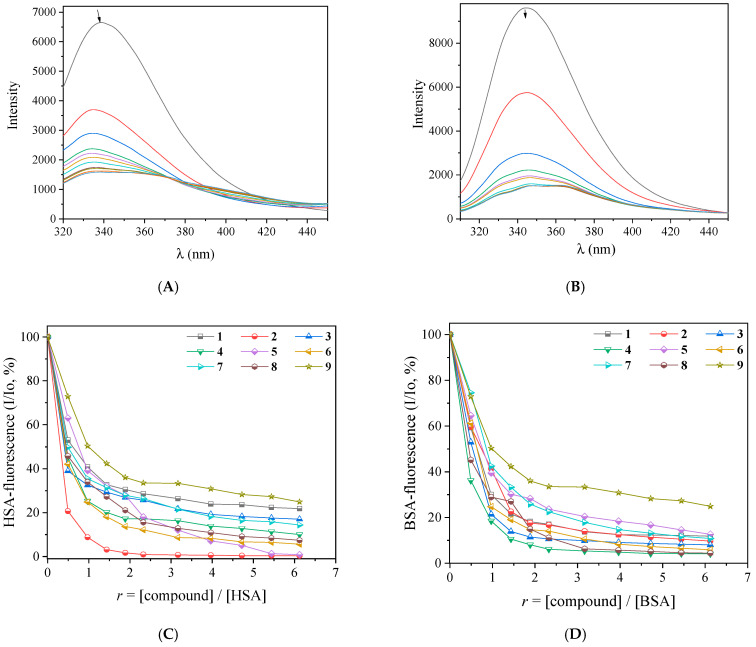
(**A**) Fluorescence emission spectra (λ_excitation_ = 295 nm) of a buffer solution (150 mM NaCl and 15 mM trisodium citrate at pH 7.0) of HSA (3 μM) in the presence of increasing amounts of complex **1**. The colors in the figure represent different concentrations of the complex and the arrow shows the changes in intensity upon increasing amounts of the complex. (**B**) Fluorescence emission spectra (λ_excitation_ = 295 nm) of a buffer solution (150 mM NaCl and 15 mM trisodium citrate at pH 7.0) of BSA (3 μM) in the presence of increasing amounts of complex **1**. The colors in the figure represent different concentrations of the complex and the arrow shows the changes in intensity upon increasing amounts of the complex. (**C**) Plot of % relative HSA fluorescence emission intensity (I/Io %) at λ_em,max_ = 340 nm versus *r* (= [compound]/[HSA]) for complexes **1**–**9** (up to 21.8% of the initial HSA fluorescence for **1**, 0.3% for **2**, 17.0% for **3**, 10.0% for **4**, 0.8% for **5**, 5.6% for **6**, 14.4% for **7**, 7.4% for **8**, and 24.9% for **9**). (**D**) Plot of % relative BSA fluorescence emission intensity (I/Io, %) at λ_em,max_ = 345 nm versus *r* (= [compound]/[BSA]) for complexes **1**–**9** (up to 11.8% of the initial BSA fluorescence for **1**, 9.7% for **2**, 8.0% for **3**, 4.1% for **4**, 12.7% for **5**, 5.9% for **6**, 10.8% for **7**, 4.4% for **8**, and 24.8% for **9**).

The fluorescence emission spectra of the albumins (3 mM) in buffer solution were recorded in the range of 300–500 nm for λ_excitation_ = 295 nm. The incremental addition of complexes **1**–**9** leads to an intense decrease in fluorescence intensity of the albumin emission band at λ_max,emission_ = 340 nm for HSA and 345 nm for BSA (shown representatively for complex **1** in Figure 11A,B). For complexes **5**–**9** bearing the neoc co-ligand, an additional emission band appeared at 370 nm (representatively shown in Figures S11 and S12). In order to evaluate this interaction further, the fluorescence emission spectra of free complexes **1**–**9** were also recorded (with λ_excitation_ = 295 nm) and afterward were subtracted from the overall spectra. The inner filter effect was also checked with Equation (S4) [80], and it was found to be negligible. The overall quenching observed was significantly high (for complex **2**, it was up to 99.7% of the fluorescence intensity initially measured for albumin, Figure 11C,D); such quenching may be assigned to re-arrangement or modifications of the secondary structure of the albumin resulting from its interaction with complexes **1**–**9** [73,79].

The values of the corresponding Stern–Volmer constant (K_SV_) and the SA–quenching constant (k_q_) for the interaction with both albumins were calculated for complexes **1**–**9** with the Stern–Volmer equation (Equations (S2) and (S3)) and the corresponding plots (Figures S13 and S14). The calculated k_q_ values of complexes **1**–**9** (Table 4) are much higher than the value of 10^10^ M^−1^s^−1^ and indicate the presence of a static quenching mechanism which subsequently confirms their interaction with both albumins [73].

The SA binding constants (K) for the compounds were determined using the Scatchard equation (Equation (S5)) and the corresponding plots (Figures S15 and S16). The values of K of complexes **1**–**9** (Table 4) are relatively high, of the magnitude 10^5^–10^6^ M^–1^, and similar to the values reported for other metal–NSAID complexes [11,41,45,48]. Complexes **2** and **3** possess the highest HSA binding (K_(HSA),**2**_ = 1.92 (±0.10) × 10^6^ M^–1^) and BSA binding (K_(BSA),**3**_ = 2.71 (±0.16) × 10^6^ M^–1^) constants, respectively, among the compounds under study. These values calculated for complexes **1**–**9** satisfy the condition of a reversible binding (including binding, safe transport, and potential release to the potential target) to albumins, since they are much lower than the binding constant of various compounds with avidin (K ≈ 10^15^ M^–1^) which is known to form among the strongest non-covalent interactions [81].

## 3. Materials and Methods

### 3.1. Materials—Instrumentation—Physical Measurements

The reagents Hfluf, Hmef, Na meclf, phen, py, CT DNA, EB, BSA, HSA, ABTS, and trolox were purchased from Sigma–Aldrich Co. (St. Louis, MI, USA), MnCl_2_∙4H_2_O NaCl, KOH and trisodium citrate were purchased from Merck (Rahway, NJ, USA), Na dicl and Htolf were purchased from Tokyo Chemical Industry (TCI Europe, Zwijndrecht, Belgium), neoc was purchased from FluoroChem company (Fluorochem UK, Hadfield, United Kingdom), and H_2_difl was purchased from Fluka (Buchs, Switzerland). All the reagents and solvents were of reagent grade and were used as purchased from commercial sources without any further purification.

The stock solution of CT DNA was prepared by dissolving CT DNA into a buffer solution (containing 150 mM NaCl and 15 mM trisodium citrate at pH 7.0), which was followed by exhaustive stirring. The CT DNA stock solution was kept at 4 °C for no longer than 2 weeks. This stock solution gave a ratio of UV absorbance at 260 and 280 nm (A_260_/A_280_) in the range of 1.85–1.90, an indication that DNA was sufficiently free of protein contamination [82]. The DNA concentration was determined by measuring the UV absorbance at 260 nm after 1:20 dilution using ε = 6600 M^–1^cm^–1^ [83].

IR spectra were recorded in the range (400–4000 cm^–1^) on a Nicolet FT-IR 6700 spectrometer with samples prepared as KBr pellets (abbreviations used: vs = very strong; s = strong; m = medium; Δ*ν*(COO) = *v*_asym_(COO) − *v*_sym_(COO)). UV-vis spectra were recorded as nujol mulls and in solution at concentrations in the range of 10^–5^ to 10^–3^ M on a Hitachi U–2001 dual beam spectrophotometer. C, H, and N elemental analysis were performed on a PerkinElmer 240B elemental analyzer (PerkinElmer, Waltham, MA, USA). Molar conductivity measurements (1 mM DMSO solution of the complexes) were carried out with a Crison Basic 30 conductometer (Crison Instruments, Barcelona, Spain). The fluorescence emission spectra were recorded in solution on a Hitachi (Hitachi High-Tech Corporation, Ibaraki, Japan) F–7000 fluorescence spectrophotometer. Viscosity experiments were carried out using an ALPHA L Fungilab rotational viscometer (Fungilab, Barcelona, Spain) equipped with an 18 mL LCP spindle, and the measurements were performed at 100 rpm.

### 3.2. Synthesis of the Complexes

#### 3.2.1. Synthesis of the Complexes Bearing N Donors (Complexes **1** and **2**)

Complexes **1**–**2** were prepared using a similar procedure. More specifically, a methanolic solution (10 mL) containing a salt of the corresponding NSAID (0.4 mmol, either used as purchased or formed in situ by the addition of KOH into a solution of the NSAID) was added into a methanolic solution (~10 mL) of MnCl_2_·6H_2_O (0.2 mmol, 39 mg) followed by the addition of 2 mL of pyridine. The resultant solution was stirred for an hour and was left for slow evaporation at room temperature.

**[Μn(meclf–O)_2_(py)_2_(H_2_O)_2_]∙2py (complex 1)**: Na meclf (0.4 mmol, 127 mg) was used as the salt of the NSAID. Colorless single crystals of complex **1** suitable for X-ray crystallography were isolated after 20 days. Yield: 90 mg, 45%. Anal. calcd for [Μn(meclf)_2_(py)_2_(H_2_O)_2_]∙2py (C_48_H_44_Cl_4_MnN_6_O_6_, MW = 997.65): C 57.79, H 4.45, N 8.42; found: C 57.61, H 4.35, N 8.30%. IR (KBr disk), v_max_/cm^−1^: v_asym_(COO): 1579 (s); v_sym_(COO): 1385 (m); Δv(COO) = 194; ρ(C–H)_py_ = 699 (s). UV-vis: as nujol mull, λ/nm: 303; in DMSO solution, λ/nm (ε/M^−1^cm^−1^): 306 (3100). The complex is soluble in DMSO and DMF and is non-electrolyte (Λ_M_ = 9 mho∙cm^2^∙mol^–1^ in 1 mM DMSO).

**[Μn(mef–O)_2_(py)_2_(H_2_O)(MeOH)]∙1.5py (complex 2)**: KOH (0.4 mmol, 0.4 mL of 1 M solution) and Hmef (0.4 mmol, 97 mg) were used for the formation of salt of the NSAID. Colorless single crystals of complex **2** suitable for X-ray crystallography were isolated after 3 weeks. Yield: 75 mg, 45%. Anal. calcd for [Μn(mef)_2_(py)_2_(H_2_O)(MeOH)]∙1.5py (C_48.50_H_51.50_MnN_5.50_O_6_, MW = 862.40): C 67.55, H 6.02, N 8.93; found: C 67.35, H 5.85, N 8.70%. IR (KBr disk), v_max_/cm^−1^: v_asym_(COO): 1581 (s); v_sym_(COO): 1389 (s); Δv(COO) = 192; ρ(C–H)_py_ = 701 (s). UV-vis: as nujol mull, λ/nm: 331, 302; in DMSO solution, λ/nm (ε/M^−1^cm^−1^): 338 (5100), 306 (12500). The complex is soluble in DMSO and DMF and is non-electrolyte (Λ_M_ = 8 mho∙cm^2^∙mol^–1^ in 1 mM DMSO).

#### 3.2.2. Synthesis of the Complexes Bearing *N,N’*–Donors (Complexes **3**–**9**)

Complexes **3**–**9** were prepared following a similar procedure. More specifically, a methanolic solution (5–10 mL) containing a salt of the corresponding NSAID (0.4 mmol, either used as purchased or generated in situ by the addition of KOH into a solution of the NSAID) was added into a methanolic solution (∼10 mL) of MnCl_2_·6H_2_O (0.2 mmol, 39 mg) followed by the addition of the corresponding *N,N’*–donor (0.2 mmol) (i.e., neoc, phen, bipy). After stirring for 1 h, the reaction solution was left to evaporate slowly at room temperature.

**[Μn(meclf–O)_2_(phen)(MeOH)_2_] (complex 3)**: Na meclf (0.4 mmol, 127 mg) was used as the NSAID salt, and phen (0.2 mmol, 36 mg) was the corresponding *N,N’*–donor. Yellow single crystals of complex **3** suitable for X-ray crystallography were isolated after 24 h. Yield: 125 mg, 70%. Anal. calcd for [Μn(meclf)_2_(phen)(MeOH)_2_] (C_42_H_36_Cl_4_MnN_4_O_6_, MW = 889.51): C 56.71, H 4.08, N 6.30; found: C 56.88, H 3.95, N 6.18%. IR (KBr disk), v_max_/cm^−1^: v_asym_(COO): 1579 (s); v_sym_(COO): 1382 (m); Δv(COO) = 197; ρ(C–H)_phen_ = 729 (s). UV-vis: as nujol mull, λ/nm: 307; in DMSO solution, λ/nm (ε/M^−1^cm^−1^): 310 (5700). The complex is soluble in DMSO, and DMF and is non-electrolyte (Λ_M_ = 7 mho∙cm^2^∙mol^–1^ in 1 mM DMSO).

**[Mn(meclf–O)_2_(bipy)(MeOH)_2_] (complex 4)**: Na meclf (0.4 mmol, 127 mg) was used as the salt of the NSAID and bipy (0.2 mmol, 31 mg) was the corresponding *N,N’*–donor. Yellowish single crystals of [Mn(meclf)_2_(bipy)(MeOH)_2_] suitable for X-ray structure determination were deposited after 2 weeks. Yield: 110 mg, 64%. Anal. calcd for [Mn(meclo)_2_(bipy)(MeOH)_2_] (C_40_H_36_Cl_4_MnN_4_O_6_, MW = 865.49): C 55.51, H 4.19, N 6.47; found: C 55.67, H 4.03, N 6.29%. IR (KBr disk), v_max_/cm^−1^: v_asym_(COO): 1580 (s); v_sym_(COO): 1383 (s); Δ_v_(COO) = 197; ρ(C–H)_bipy_ = 761 (s). UV-vis: as nujol mull, λ/nm: 315, 293; in DMSO solution, λ/nm (ε/M^−1^cm^−1^): 319 (16000), 287 (25000). The complex is soluble in DMSO and DMF and is non-electrolyte (Λ_M_ = 6 mho∙cm^2^∙mol^–1^ in 1 mM DMSO).

**[Μn(dicl–O,O’)_2_(neoc)]∙0.25 H_2_O (complex 5)**: Na dicl (0.4 mmol, 92 mg) was used as the NSAID salt and neoc (0.2 mmol, 46 mg) was the corresponding *N,N’*–donor. Yellow single crystals, suitable for X-ray structure determination, were collected after 2 days. Yield: 105 mg, 60%. Anal. calcd for [Μn(dicl)_2_(neoc)]∙0.25 H_2_O (C_42_H_32.5_Cl_4_MnN_4_O_4.25_, MW = 857.98): C 58.80, H 3.82, N 6.53; found: C 58.70, H 3.75, N 6.67%. IR (KBr disk), v_max_/cm^−1^: v_asym_(COO): 1590 (s); v_sym_(COO): 1415 (s); Δ_v_(COO) = 175; ρ(C–H)_neoc_ = 731 (m). UV-vis: as nujol mull, λ/nm: 370, 289, 275; in DMSO solution, λ/nm (ε/M^−1^cm^−1^): 368 (1700), 285 (13300), 271(14400). The complex is soluble in DMSO and is non-electrolyte (Λ_M_ = 8 mho∙cm^2^∙mol^–1^ in 1 mM DMSO).

**[Μn(mef–O,O’)_2_(neoc)]∙1.5MeOH∙0.25 H_2_O (complex 6)**: KOH (0.4 mmol, 0.4 mL of 1 M solution) and Hmef (0.4 mmol, 97 mg) were used for the formation of NSAID salt and neoc (0.2 mmol, 46 mg) was the corresponding *N,N’*–donor. Pale-yellow single crystals of complex **6** suitable for X-ray crystallography were isolated after 2 days. Yield: 70 mg, 45%. Anal. calcd for [Μn(mef)_2_(neoc)]∙1.5MeOH∙0.25 H_2_O (C_45.5_H_46.5_MnN_4_O_5.75_, MW = 796.32): C 68.63, H 5.89, N 7.04; found: C 68.50, H 5.75, N 6.81%. IR (KBr disk), v_max_/cm^−1^: v_asym_(COO): 1582 (s); v_sym_(COO): 1396 (s); Δ_v_(COO) = 186; ρ(C–H)_neoc_ = 731 (s). UV-vis: as nujol mull, λ/nm: 334, 290, 275; in DMSO solution, λ/nm (ε/M^−1^cm^−1^): 339(sh) (3200), 291 (11800), 272 (11700). The complex is soluble in DMSO and is non-electrolyte (Λ_M_ = 9 mho∙cm^2^∙mol^–1^ in 1 mM DMSO).

**[Μn(fluf–O,O’)_2_(neoc)] (complex 7)**: KOH (0.4 mmol, 0.4 mL of 1 M solution) and Hfluf (0.4 mmol, 112 mg) were used for the formation of NSAID salt and neoc (0.2 mmol, 46 mg) was the corresponding *N,N’*–donor. Yellowish single crystals of complex **7**, suitable for X-ray crystallography, were collected after one month. Yield: 115 mg, 70%. Anal. calcd for [Μn(fluf)_2_(neoc)] (C_42_H_30_F_6_MnN_4_O_4_, MW = 823.64). C 61.25, H 3.67, N 6.80; found: C 61.40, H 3.55, N 6.68%. IR (KBr disk), v_max_/cm^−1^: v_asym_(COO): 1582 (s); v_sym_(COO): 1397 (vs); Δ_v_(COO) = 185; ρ(C–H)_neoc_ = 729 (s). UV-vis: as nujol mull, λ/nm: 279; in DMSO solution, λ/nm (ε/M^−1^cm^−1^): 275 (15300). The complex is soluble in DMSO and is non-electrolyte (Λ_M_ = 10 mho∙cm^2^∙mol^–1^ in 1 mM DMSO).

**[Μn(tolf–O,O’)_2_(neoc)] (complex 8)**: KOH (0.4 mmol, 0.4 mL of 1 M solution) and Htolf (0.4 mmol, 104 mg) were used for the formation of NSAID salt and neoc (0.2 mmol, 46 mg) was the corresponding *N,N’* donor. Yellow single crystals of [Μn(tolf–O,O’)_2_(neoc)], **8** suitable for X-ray structure determination were deposited after 2 days. Yield: 100 mg, 64%. Anal. calcd for [Μn(tolf)_2_(neoc)] (C_42_H_34_Cl_2_MnN_4_O_4_, MW = 784.59): C 64.30, H 4.37, N 7.14; found: C 64.18, H 4.25, N 7.27%. IR (KBr disk), v_max_/cm^−1^: v_asym_(COO): 1580 (s); v_sym_(COO): 1390 (vs); Δ_v_(COO) = 190; ρ(C–H)_neoc_ = 730 (m). UV-vis: as nujol mull, λ/nm: 300, 279; in DMSO solution, λ/nm (ε/M^−1^cm^−1^): 298 (22900), 272 (21600). The complex is soluble in DMSO and is non-electrolyte (Λ_M_ = 9 mho∙cm^2^∙mol^–1^ in 1 mM DMSO).

**[Μn(Hdifl–O,O’)(Hdifl–O)(neoc)]·0.5MeOH (complex 9)**: KOH (0.4 mmol, 0.4 mL of 1 M solution) and H_2_difl (0.4 mmol, 100 mg) were used for the formation of NSAID salt and neoc (0.2 mmol, 46 mg) was the corresponding *N,N’*–donor. Yellow single crystals of complex **9** suitable for X-ray crystallography were collected after 10 days. Yield: 75 mg, 48%. Anal. calcd for [Μn(Hdifl)_2_(neoc)]·0.5MeOH (C_40.50_H_28_F_4_MnN_2_O_6.50_, MW = 777.58). C 62.56, H 3.63, N 3.60; found: C 62.75, H 3.50, N 3.65%. IR (KBr disk), v_max_/cm^−1^: v_asym_(COO): 1595 (m); v_sym_(COO): 1413 (m); 1379 (m); Δv(COO) = 182, 216; ρ(C–H)_neoc_ = 732 (m). UV-vis: as nujol mull, λ/nm: 273; in DMSO solution, λ/nm (ε/M^−1^cm^−1^): 270 (17100). The complex is soluble in DMSO and is non-electrolyte (Λ_M_ = 7 mho∙cm^2^∙mol^–1^ in 1 mM DMSO).

### 3.3. Single-Crystal X-Ray Crystallography

For the structural determination of complexes **1**–**9**, single crystals suitable for crystal structure analysis were mounted at room temperature on a Bruker Kappa APEX2 diffractometer equipped with a Triumph monochromator using Mo Kα (λ = 0.71073 Å, source operating at 50 kV and 30 mA) radiation. Unit cell dimensions were determined and refined by using the angular settings of at least 112 and up to 514 high-intensity reflections (>10σ(I)) in the range of 11 < 2θ < 36. Intensity data were recorded using φ and ω scans. All crystals presented no decay during the data collection. The frames collected for each crystal were integrated with the Bruker SAINT Software package version 1.0 [84] using a narrow-frame algorithm. Data were corrected for absorption using the numerical method (SADABS) based on crystal dimensions [85]. All structures were solved using SUPERFLIP [86] incorporated in Crystals. Data refinement (full-matrix least-squares methods on F2) and all subsequent calculations were carried out using the Crystals version 14.61 build 6236 program package [87]. All non-hydrogen non-disordered atoms were refined anisotropically. For all disordered atoms (from solvate molecules in complexes **2**, **5**, **6** and fluorine atoms in complex **7**), a typical refinement procedure concerning the occupation factors and, then, positions and isotropic/anisotropic displacement factors has been followed. Hydrogen atoms riding on non-disordered parent atoms were located from different Fourier maps and refined at idealized positions riding on the parent atoms with isotropic displacement parameters Uiso(H) = 1.2Ueq(C) or 1.5Ueq (methyl and -OH hydrogens) and at distances C–H 0.95 Å and O–H 0.82 Å. All methyl and OH hydrogen atoms were allowed to rotate. Hydrogen atoms riding on disordered oxygen atoms of water and methanol solvent molecules were positioned geometrically, when possible, to fulfill hydrogen bonding demands. The remaining methyl-aromatic hydrogen atoms were positioned geometrically to their parent atoms.

Important crystallographic and refinement data for complexes **1**–**9** are listed in Tables S1–S5. Further details on the crystallographic studies as well as atomic displacement parameters are given as Supplementary Materials File S1 in the form of cif files.

CCDC deposition numbers 2404798–2404806 contain the supplementary crystallographic data for the complexes. These data can be obtained free of charge via www.ccdc.cam.ac.uk/conts/retrieving.html (or from the Cambridge Crystallographic Data Centre, 12 Union Road, Cambridge CB21EZ, UK; fax: (+44) 1223–336–033; or deposit@ccdc.cam.ac.uk).

### 3.4. In Vitro Biological Activity Studies

In order to study in vitro the biological activity (interaction with DNA or albumins, antioxidant activity) of complexes **1**–**9**, the compounds were dissolved in DMSO (1 mM) due to their low aqueous solubility. Mixing of each solution with the aqueous buffer solution of DNA or albumins used in the studies never exceeded 5% DMSO (*v*/*v*) in the final solution. Control experiments with DMSO were performed, and no significant effect on the measurements was observed.

The antioxidant activity of the complexes was evaluated by determining their ability to scavenge ABTS free radicals (expressed as a percentage of radical scavenging, ABTS%). The interaction of the complexes with CT DNA was investigated by UV-vis spectroscopy, viscosity measurements, and via the evaluation of their EB-displacing ability, which was monitored by fluorescence emission spectroscopy. The serum albumin (BSA or HSA) binding studies were performed using tryptophan fluorescence quenching experiments. Detailed procedures [70,73,75,80,88,89] regarding the in vitro study of the biological activity of the complexes are given in the ESI file (Sections S1–S3).

## 4. Conclusions

Nine manganese(II) coordination compounds with a series of carboxylate NSAIDs as ligands have been isolated and characterized in the presence of diverse nitrogen donors as co-ligands. The molecular structures of all complexes **1**–**9** were determined using single-crystal X-ray crystallography. In these complexes, the NSAIDs are simply deprotonated ligands in a monodentate or bidentate chelating coordination fashion. In complexes **1**–**8**, the manganese(II) ion is six-coordinate in a distorted octahedral environment, while in complex **9**, a distorted square pyramidal geometry around five-coordinate Mn(II) is observed.

The biological activity of the complexes included the evaluation of their interaction with albumins and CT DNA, as well as the scavenging of ABTS radicals. The complexes may interact with CT DNA in an intercalative mode, and complex **7** has the highest DNA binding constant (K_b_ = 1.10 (±0.02) × 10^6^ M^–1^). The binding of complexes **1**–**9** to serum albumins is reversible and tight, revealing the potency for transportation toward potential biological targets where they may be released. Regarding the ABTS scavenging activity, almost all complexes are better scavengers than the corresponding free NSAIDs. The Mn(II) complexes with mefenamato, diclofenac, and tolfenamato ligands (complexes **2**, **4**–**6**, and **8**) have a significantly high ability to scavenge ABTS radicals and are more active toward ABTS radicals than the reference compound trolox. [Μn(tolf)_2_(neoc)] (complex **8**) is the best ABTS scavenger (ABTS% = 99.63 ± 0.07%) among almost all metal complexes with NSAID ligands reported till now.

In conclusion, the herein studied Mn(II)–NSAID complexes bear significant overall radical scavenging ability, interact tightly with CT DNA, and bind tightly and reversibly to albumins. Such biological activity is usually a necessary condition for potential biological activity (e.g., anticancer), and the herein reported compounds deserve the attention for further and elaborate biological studies, such as anticholinergic activity and cytotoxicity studies.

## Figures and Tables

**Table 1:** % ABTS radical scavenging activity (ABTS%) of complexes **1**–**9** and the corresponding NSAIDs. Trolox is the reference compound.

Compound	ABTS%
Complex **1**	39.18 ± 3.27
Complex **2**	92.56 ± 0.74
Complex **3**	18.66 ± 0.71
Complex **4**	98.69 ± 0.06
Complex **5**	99.20 ± 0.06
Complex **6**	98.69 ± 0.07
Complex **7**	29.70 ± 2.86
Complex **8**	99.63 ± 0.07
Complex **9**	25.80 ± 1.43
Na meclf [39]	59.48 ± 0.06
Hmef [39]	66.32 ± 0.38
Na dicl [40]	76.35 ± 0.75
Hfluf [39]	64.57 ± 0.43
Htolf [39]	59.43 ± 0.33
H_2_difl [40]	76.58 ± 0.74
Trolox	91.8 ± 0.17

Each experiment was performed at least in triplicate, SD < ±10%.

**Table 2:** UV-vis spectra features from the interaction study of complexes **1**–**9** with CT DNA. UV band (λ in nm) (percentage of the observed hyper-/hypo-chromism (ΔA/A_0_, %), blue/red shift of the λ_max_ (Δλ, nm)) and DNA binding constants (K_b_).

Compound	λ_max_ (nm) (ΔA/A_0_ (%) ^a^, Δλ (nm) ^b^)	K_b_ (M^–1^)
Complex **1**	306 (+10, −2)	5.16 (±0.09) × 10^5^
Complex **2**	306 (+7, 0); 345(<−50 ^c^, elim ^d^)	3.71 (±0.15) × 10^5^
Complex **3**	307(−6, +2)	1.62 (±0.04) × 10^4^
Complex **4**	287 (+15, −7); 319(+9, −2)	6.25 (±0.35) × 10^4^
Complex **5**	272 (+8, +2); 285(sh) (+7, −4)	6.58 (±0.20) × 10^5^
Complex **6**	271 (+3, 0); 289 (+2, −2)	2.48 (±0.33) × 10^4^
Complex **7**	275 (−6, 0)	1.10 (±0.02) × 10^6^
Complex **8**	271 (+5, +1); 296 (+2 +2)	4.15 (±0.23) × 10^4^
Complex **9**	269 (+6, 0)	2.24 (±0.71) × 10^5^
Na meclf [48]	302(−12, −1); 315(sh) (+5, −2)	1.51 (±0.12) × 10^5^
Hmef [39]	324(+10, 0)	1.05 (±0.02) × 10^5^
Na dicl [40]	295(−7.5, −5)	3.16 (±0.14) × 10^4^
Hfluf [39]	292(+40, +10); 344(<−50, 0)	2.70 (±0.11) × 10^5^
Htolf [39]	304 (+40, +5); 348 (<−50, −2)	5.00 (±0.10) × 10^4^
H_2_difl [40]	295 (+15, +2)	3.08 (±0.15) × 10^3^

^a^ “+” denotes hyperchromism, “−” denotes hypochromism. ^b^ “+” denotes red shift, “−” denotes blue shift. ^c^ “<−50” denotes intense hypochromism. ^d^ “elim” denotes elimination.

**Table 3:** Fluorescence features of the EB displacement studies for complexes **1**–**9** and the corresponding NSAIDs: percentage of EB–DNA fluorescence quenching (ΔI/I_0_, in %), Stern–Volmer constants (K_SV_, in M^−1^) and quenching constants of the EB–DNA fluorescence (k_q_, in M^−1^s^−1^).

Compound	ΔI/Io (%)	K_SV_ (M^–1^)	k_q_ (M^–1^s^–1^)
Complex **1**	53.0	1.15 (±0.17) × 10^5^	4.99 (±0.15) × 10^12^
Complex **2**	52.7	2.30 (±0.53) × 10^5^	1.00 (±0.155) × 10^13^
Complex **3**	79.6	1.18 (±0.32) × 10^6^	5.15 (±0.14) × 10^13^
Complex **4**	44.8	1.54 (±0.31) × 10^5^	6.69 (±0.13) × 10^12^
Complex **5**	69.9	7.37 (±0.05) × 10^5^	3.20 (±0.21) × 10^13^
Complex **6**	60.6	4.29 (±0.32) × 10^5^	1.86 (±0.18) × 10^13^
Complex **7**	52.1	5.55(±0.12) × 10^5^	2.41 (±0.05) × 10^13^
Complex **8**	50.0	2.35 (±0.48) × 10^5^	1.02 (±0.03) × 10^13^
Complex **9**	31.6	1.01 (±0.42) × 10^6^	4.39 (±0.10) × 10^13^
Na meclf [48]	80.1	8.20 (±0.26) × 10^4^	3.57 (±0.11) × 10^12^
Hmef [39]	80.0	1.58 (±0.06) × 10^5^	6.87 (±0.26) × 10^12^
Na dicl [40]	65.0	2.47 (±0.06) × 10^5^	1.07 (±0.03) × 10^13^
Hfluf [39]	67.0	6.34 (±0.30) × 10^5^	2.76 (±0.13) × 10^13^
Htolf [39]	74.0	1.15 (±0.04) × 10^6^	5.00 (±0.17) × 10^13^
H_2_difl [40]	65.0	8.59 (±0.35) × 10^5^	3.73 (±0.15) × 10^13^

**Table 4:** The albumin (HSA/BSA) quenching constants (k_q_) and binding constants (K) for complexes **1**–**9** and the corresponding NSAIDs.

Compound	k_q(BSA)_ (M^–1^s^–1^)	Κ_(BSA)_ (M^–1^)	k_q(HSA)_ (M^–1^s^–1^)	Κ_(HSA)_ (M^–1^)
Complex **1**	6.88 (±0.05) × 10^13^	9.58 (±0.07) × 10^5^	4.05 (±0.42) × 10^13^	8.83 (±0.24) × 10^5^
Complex **2**	5.30 (±0.29) × 10^13^	1.03 (±0.16) × 10^6^	1.87 (±0.01) × 10^15^	1.92 (±0.10) × 10^6^
Complex **3**	1.34 (±0.16) × 10^14^	2.71 (±0.16) × 10^6^	2.30 (±0.91) × 10^13^	8.73 (±0.06) × 10^5^
Complex **4**	1.76 (±0.11) × 10^14^	9.61 (±0.06) × 10^5^	4.69 (±0.15) × 10^13^	9.38 (±0.29) × 10^5^
Complex **5**	3.46 (±0.16) × 10^13^	6.32 (±0.32) × 10^5^	7.67 (±0.10) × 10^13^	3.09 (±0.21) × 10^5^
Complex **6**	1.87 (±0.04) × 10^14^	1.08 (±0.38) × 10^6^	9.06 (±0.35) × 10^13^	9.48 (±0.35) × 10^5^
Complex **7**	4.62 (±0.11) × 10^13^	4.69 (±0.37) × 10^5^	3.02 (±0.15) × 10^13^	8.33 (±0.04) × 10^5^
Complex **8**	1.36 (±0.53) × 10^14^	7.23 (±0.23) × 10^5^	6.79 (±0.14) × 10^13^	6.35 (±0.29) × 10^5^
Complex **9**	2.99 (±0.16) × 10^13^	5.67 (±0.27) × 10^5^	2.97 (±0.13) × 10^12^	5.44 (±0.24) × 10^5^
Na meclf [48]	4.84 (±0.32) × 10^13^	1.78 (±0.11) × 10^6^	2.98 (±0.31) × 10^13^	1.05 (±0.03) × 10^6^
Hmef [39]	2.78 (±0.20) × 10^13^	1.35 (±0.22) × 10^5^	7.13 (±0.34) × 10^12^	1.32 (±0.15) × 10^5^
Na dicl [40]	8.11 (±0.34) × 10^12^	3.55 (±0.22) × 10^5^	1.81 (±0.17) × 10^12^	1.63 (±0.15) × 10^5^
Hfluf [39]	1.83 (±0.20) × 10^13^	1.06 (±0.04) × 10^6^	1.86 (±0.21) × 10^12^	1.79 (±0.17) × 10^5^
Htolf [39]	2.18 (±0.12) × 10^13^	1.60 (±0.14) × 10^5^	6.10 (±0.38) × 10^12^	3.12 (±0.25) × 10^5^
H_2_difl [40]	1.53 (±0.08) × 10^13^	1.93 (±0.15) × 10^5^	2.67 (±0.16) × 10^12^	1.22 (±0.07) × 10^5^

## Data Availability

Data contained within the article or Supplementary Materials.

## Supplementary Materials

The following Supporting Information can be downloaded at: https://www.mdpi.com/article/10.3390/ijms252413457/s1. References [88,89] are cited in the Supplementary Materials.

## Abbreviations

ABTS	2,2′–azinobis(3–ethylbenzothiazoline–6–sulfonic acid) radical cation
ABTS%	ABTS scavenging activity
bipy	2,2′–bipyridine
BSA	bovine serum albumin
CT	calf–thymus
dicl^–^	anion of diclofenac
difl^–2^	dianion of diflunisal
EB	ethidium bromide, 3,8–diamino–5–ethyl–6–phenyl–phenanthridinium bromide
fluf^–^	flufenamato anion
Hdicl	diclofenac
Hdifl^−^	anion of diflunisal
Hfluf	flufenamic acid
Hmeclf	meclofenamic acid
Hmef	mefenamic acid
HSA	human serum albumin
Htolf	tolfenamic acid
H_2_difl	diflunisal
K	SA binding constant
K_b_	DNA binding constant
k_q_	quenching constant
K_SV_	Stern–Volmer constant
meclf^−^	meclofenamato anion
mef^−^	mefenamato anion
neoc	neocuproine, 2,9–dimethyl–1,10–phenanthroline
NSAID	non-steroidal anti-inflammatory drug
phen	1,10–phenqnthroline
py	pyridine
r	[compound]/[DNA] or [SA] ratio
SA	serum albumin
trolox	6–hydroxy–2,5,7,8–tetramethylchromane–2–carboxylic acid
tolf^−^	tolfenamato anion
Δν(COO)	ν_asym_(COO) − ν_sym_(COO)
