# Supplementary material for: Manganese(II) Complexes with Non-Steroidal Anti-Inflammatory Drugs: Structure and Biological Activity
Source: Int J Mol Sci. 2024 Dec 16;25(24):13457. doi: 10.3390/ijms252413457 (PMC11676910; doi:10.3390/ijms252413457)

checkCIF/PLATON report

Structure factors have been supplied for datablock(s) I

THIS REPORT IS FOR GUIDANCE ONLY. IF USED AS PART OF A REVIEW PROCEDURE FOR PUBLICATION, IT SHOULD NOT REPLACE THE EXPERTISE OF AN EXPERIENCED CRYSTALLOGRAPHIC REFEREE.

No syntax errors found. CIF dictionary Interpreting this report

Datablock: I

Bond precision: C-C = 0.0056 A Wavelength=0.71073

Cell:	a=13.7438(14)	b=15.908(2)	c=20.007(3)
	alpha=71.419(7)	beta=87.773(6)	gamma=84.475(6)
Temperature:	140 K		

	Calculated	Reported
Volume	4126.8(9)	4126.7(9)
Space group	P -1	P -1
Hall group	-P 1	?
Moiety formula	4(C44 H40 Mn N4 O4), 6(C H4 O), H2 O	C91 H93 Mn2 N8 O11.50
Sum formula	C182 H186 Mn4 N16 O23	C91 H93 Mn2 N8 O11.50
Mr	3185.23	1592.64
Dx,g cm-3	1.282	1.282
Z	1	2
Mu (mm-1)	0.372	0.372
F000	1674.0	1674.0
F000’	1676.01	
h,k,lmax	16,19,24	16,19,24
Nref	16352	15904
Tmin,Tmax	0.935,0.963	0.950,0.960
Tmin’	0.908	

Correction method= # Reported T Limits: Tmin=0.950 Tmax=0.960
AbsCorr = NUMERICAL

Data completeness= 0.973 Theta(max)= 26.075

R(reflections)= 0.0526(10630) wR2(reflections)= 0.1195(10630)

S = 1.000 Npar= 1007

The following ALERTS were generated. Each ALERT has the format
test-name_ALERT_alert-type_alert-level.
Click on the hyperlinks for more details of the test.

Alert level C

```
PLAT220_ALERT_2_C NonSolvent   Resd 1  C   Ueq(max)/Ueq(min) Range        3.2 Ratio
PLAT911_ALERT_3_C Missing FCF Refl Between Thmin & STh/L=    0.600        115 Report
```

Alert level G

```
PLAT002_ALERT_2_G Number of Distance or Angle Restraints on AtSite         12 Note
PLAT007_ALERT_5_G Number of Unrefined Donor-H Atoms ..............         12 Report
PLAT042_ALERT_1_G Calc. and Reported MoietyFormula Strings  Differ     Please Check
PLAT045_ALERT_1_G Calculated and Reported Z Differ by a Factor ...       0.50 Check
PLAT300_ALERT_4_G Atom Site Occupancy of O10        Constrained at        0.5 Check
PLAT300_ALERT_4_G Atom Site Occupancy of C89        Constrained at        0.5 Check
PLAT300_ALERT_4_G Atom Site Occupancy of H101       Constrained at        0.5 Check
PLAT300_ALERT_4_G Atom Site Occupancy of H891       Constrained at        0.5 Check
PLAT300_ALERT_4_G Atom Site Occupancy of H892       Constrained at        0.5 Check
PLAT300_ALERT_4_G Atom Site Occupancy of H893       Constrained at        0.5 Check
PLAT300_ALERT_4_G Atom Site Occupancy of O11        Constrained at        0.5 Check
PLAT300_ALERT_4_G Atom Site Occupancy of C90        Constrained at        0.5 Check
PLAT300_ALERT_4_G Atom Site Occupancy of H901       Constrained at        0.5 Check
PLAT300_ALERT_4_G Atom Site Occupancy of H902       Constrained at        0.5 Check
PLAT300_ALERT_4_G Atom Site Occupancy of H903       Constrained at        0.5 Check
PLAT300_ALERT_4_G Atom Site Occupancy of H947       Constrained at        0.5 Check
PLAT300_ALERT_4_G Atom Site Occupancy of O12        Constrained at        0.5 Check
PLAT300_ALERT_4_G Atom Site Occupancy of C91        Constrained at        0.5 Check
PLAT300_ALERT_4_G Atom Site Occupancy of H911       Constrained at        0.5 Check
PLAT300_ALERT_4_G Atom Site Occupancy of H912       Constrained at        0.5 Check
PLAT300_ALERT_4_G Atom Site Occupancy of H913       Constrained at        0.5 Check
PLAT300_ALERT_4_G Atom Site Occupancy of H948       Constrained at        0.5 Check
PLAT300_ALERT_4_G Atom Site Occupancy of O13        Constrained at        0.5 Check
PLAT300_ALERT_4_G Atom Site Occupancy of C92        Constrained at        0.5 Check
PLAT300_ALERT_4_G Atom Site Occupancy of H921       Constrained at        0.5 Check
PLAT300_ALERT_4_G Atom Site Occupancy of H922       Constrained at        0.5 Check
PLAT300_ALERT_4_G Atom Site Occupancy of H923       Constrained at        0.5 Check
PLAT300_ALERT_4_G Atom Site Occupancy of H949       Constrained at        0.5 Check
PLAT300_ALERT_4_G Atom Site Occupancy of O14        Constrained at        0.5 Check
PLAT300_ALERT_4_G Atom Site Occupancy of C93        Constrained at        0.5 Check
PLAT300_ALERT_4_G Atom Site Occupancy of H931       Constrained at        0.5 Check
PLAT300_ALERT_4_G Atom Site Occupancy of H932       Constrained at        0.5 Check
PLAT300_ALERT_4_G Atom Site Occupancy of H933       Constrained at        0.5 Check
PLAT300_ALERT_4_G Atom Site Occupancy of H946       Constrained at        0.5 Check
PLAT300_ALERT_4_G Atom Site Occupancy of O15        Constrained at        0.5 Check
PLAT300_ALERT_4_G Atom Site Occupancy of C94        Constrained at        0.5 Check
PLAT300_ALERT_4_G Atom Site Occupancy of H941       Constrained at        0.5 Check
PLAT300_ALERT_4_G Atom Site Occupancy of H942       Constrained at        0.5 Check
PLAT300_ALERT_4_G Atom Site Occupancy of H943       Constrained at        0.5 Check
PLAT300_ALERT_4_G Atom Site Occupancy of H950       Constrained at        0.5 Check
PLAT300_ALERT_4_G Atom Site Occupancy of O9         Constrained at        0.5 Check
PLAT300_ALERT_4_G Atom Site Occupancy of H91        Constrained at        0.5 Check
PLAT300_ALERT_4_G Atom Site Occupancy of H92        Constrained at        0.5 Check
PLAT302_ALERT_4_G Anion/Solvent/Minor-Residue Disorder (Resd  3  )       100% Note
PLAT302_ALERT_4_G Anion/Solvent/Minor-Residue Disorder (Resd  4  )       100% Note
PLAT302_ALERT_4_G Anion/Solvent/Minor-Residue Disorder (Resd  5  )       100% Note
PLAT302_ALERT_4_G Anion/Solvent/Minor-Residue Disorder (Resd  6  )       100% Note
PLAT302_ALERT_4_G Anion/Solvent/Minor-Residue Disorder (Resd  7  )       100% Note
PLAT302_ALERT_4_G Anion/Solvent/Minor-Residue Disorder (Resd  8  )       100% Note
PLAT302_ALERT_4_G Anion/Solvent/Minor-Residue Disorder (Resd  9  )       100% Note
```

```
PLAT304_ALERT_4_G Non-Integer Number of Atoms in ..... (Resd  9  )       1.50 Check
PLAT415_ALERT_2_G Short Inter D-H..H-X       H91      ..H551     .       1.43 Ang.
                                                 1+x,-1+y,z  =      1_645 Check
PLAT415_ALERT_2_G Short Inter D-H..H-X       H91      ..H561     .       1.82 Ang.
                                                 1+x,-1+y,z  =      1_645 Check
PLAT415_ALERT_2_G Short Inter D-H..H-X       H92      ..H561     .       2.09 Ang.
                                                 1+x,-1+y,z  =      1_645 Check
PLAT432_ALERT_2_G Short Inter X...Y Contact  O9       ..C56              2.07 Ang.
                                                 1+x,-1+y,z  =      1_645 Check
PLAT432_ALERT_2_G Short Inter X...Y Contact  O9       ..C55              2.16 Ang.
                                                 1+x,-1+y,z  =      1_645 Check
PLAT432_ALERT_2_G Short Inter X...Y Contact  O9       ..C16              2.27 Ang.
                                                      x,y,z  =      1_555 Check
PLAT432_ALERT_2_G Short Inter X...Y Contact  O9       ..C17              2.99 Ang.
                                                      x,y,z  =      1_555 Check
PLAT432_ALERT_2_G Short Inter X...Y Contact  O12      ..C92              2.39 Ang.
                                                1-x,1-y,2-z  =      2_667 Check
PLAT769_ALERT_4_G CIF Embedded explicitly supplied scattering data     Please Note
PLAT794_ALERT_5_G Tentative Bond Valency for Mn2       (II)      .       1.94 Info
PLAT808_ALERT_5_G No Parseable SHELXL Style Weighting Scheme Found     Please Check
PLAT860_ALERT_3_G Number of Least-Squares Restraints .............          6 Note
PLAT882_ALERT_1_G No Datum for _diffrn_reflns_av_unetI/netI ......     Please Do !
PLAT912_ALERT_4_G Missing # of FCF Reflections Above STh/L=  0.600        370 Note
PLAT929_ALERT_5_G No Weight Pars,Obs and Calc R1,wR2,S not Checked          ! Info
```

---

   0 **ALERT level A** = Most likely a serious problem - resolve or explain
   0 **ALERT level B** = A potentially serious problem, consider carefully
   2 **ALERT level C** = Check. Ensure it is not caused by an omission or oversight
  66 **ALERT level G** = General information/check it is not something unexpected

   3 ALERT type 1 CIF construction/syntax error, inconsistent or missing data
  10 ALERT type 2 Indicator that the structure model may be wrong or deficient
   2 ALERT type 3 Indicator that the structure quality may be low
  49 ALERT type 4 Improvement, methodology, query or suggestion
   4 ALERT type 5 Informative message, check

It is advisable to attempt to resolve as many as possible of the alerts in all categories. Often the minor alerts point to easily fixed oversights, errors and omissions in your CIF or refinement strategy, so attention to these fine details can be worthwhile. In order to resolve some of the more serious problems it may be necessary to carry out additional measurements or structure refinements. However, the purpose of your study may justify the reported deviations and the more serious of these should normally be commented upon in the discussion or experimental section of a paper or in the "special_details" fields of the CIF. checkCIF was carefully designed to identify outliers and unusual parameters, but every test has its limitations and alerts that are not important in a particular case may appear. Conversely, the absence of alerts does not guarantee there are no aspects of the results needing attention. It is up to the individual to critically assess their own results and, if necessary, seek expert advice.

Publication of your CIF in IUCr journals

A basic structural check has been run on your CIF. These basic checks will be run on all CIFs submitted for publication in IUCr journals (*Acta Crystallographica*, *Journal of Applied Crystallography*, *Journal of Synchrotron Radiation*); however, if you intend to submit to *Acta Crystallographica Section C* or *E* or *IUCrData*, you should make sure that full publication checks are run on the final version of your CIF prior to submission.

Publication of your CIF in other journals

Please refer to the *Notes for Authors* of the relevant journal for any special instructions relating to CIF submission.

PLATON version of 05/12/2020; check.def file version of 05/12/2020

Datablock I - ellipsoid plot

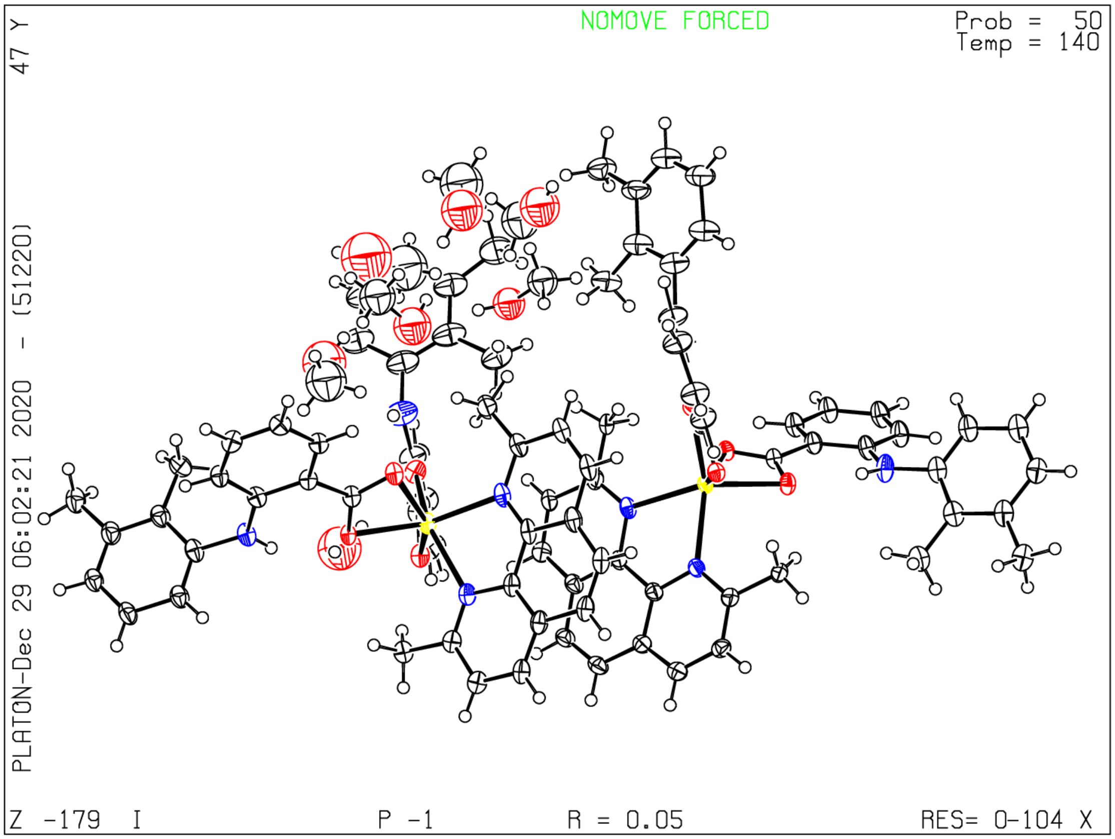

Supplement: Supplementary file 1 [file ijms-25-13457-s001.zip › Supplementary File S1/Checkcif of complex 6.pdf]
