# Supplementary material for: Manganese(II) Complexes with Non-Steroidal Anti-Inflammatory Drugs: Structure and Biological Activity
Source: Int J Mol Sci. 2024 Dec 16;25(24):13457. doi: 10.3390/ijms252413457 (PMC11676910; doi:10.3390/ijms252413457)

checkCIF/PLATON report

Structure factors have been supplied for datablock(s) I

THIS REPORT IS FOR GUIDANCE ONLY. IF USED AS PART OF A REVIEW PROCEDURE FOR PUBLICATION, IT SHOULD NOT REPLACE THE EXPERTISE OF AN EXPERIENCED CRYSTALLOGRAPHIC REFEREE.

No syntax errors found. CIF dictionary Interpreting this report

Datablock: I

```
Bond precision:  C-C = 0.0077 A         Wavelength=0.71073

Cell:              a=9.780(6)      b=13.108(8)      c=29.107(17)
                   alpha=90        beta=90          gamma=90
Temperature:       295 K

                Calculated                 Reported
Volume          3731(4)                    3731(4)
Space group     P 21 21 21                 P 21 21 21
Hall group      P 2ac 2ab                  ?
Moiety formula  C42 H30 F6 Mn N4 O4        C42 H30 F6 Mn1 N4 O4
Sum formula     C42 H30 F6 Mn N4 O4        C42 H30 F6 Mn1 N4 O4
Mr              823.64                     823.64
Dx,g cm-3       1.466                      1.466
Z               4                          4
Mu (mm-1)       0.433                      0.433
F000            1684.0                     1684.0
F000'           1686.30
h,k,lmax        11,16,35                   11,15,35
Nref            7104[ 3997]                7040
Tmin,Tmax       0.925,0.941                0.940,0.940
Tmin'           0.897

Correction method= # Reported T Limits: Tmin=0.940 Tmax=0.940
AbsCorr = NUMERICAL

Data completeness= 1.76/0.99      Theta(max)= 25.737

R(reflections)= 0.0525( 5511)     wR2(reflections)= 0.1000( 5511)

S = 0.999                  Npar= 512
```

The following ALERTS were generated. Each ALERT has the format
test-name_ALERT_alert-type_alert-level.
Click on the hyperlinks for more details of the test.

Alert level C

```
PLAT090_ALERT_3_C Poor Data / Parameter Ratio (Zmax > 18) ........       7.74 Note
PLAT241_ALERT_2_C High   'MainMol' Ueq as Compared to Neighbors of         O1 Check
PLAT241_ALERT_2_C High   'MainMol' Ueq as Compared to Neighbors of         O2 Check
PLAT241_ALERT_2_C High   'MainMol' Ueq as Compared to Neighbors of         O4 Check
PLAT242_ALERT_2_C Low    'MainMol' Ueq as Compared to Neighbors of        Mn1 Check
PLAT242_ALERT_2_C Low    'MainMol' Ueq as Compared to Neighbors of         C1 Check
PLAT341_ALERT_3_C Low Bond Precision on  C-C Bonds ...............    0.00774 Ang.
PLAT911_ALERT_3_C Missing FCF Refl Between Thmin & STh/L=    0.600         19 Report
```

Alert level G

```
PLAT003_ALERT_2_G Number of Uiso or Uij Restrained non-H Atoms ...          4 Report
PLAT007_ALERT_5_G Number of Unrefined Donor-H Atoms ..............          2 Report
PLAT232_ALERT_2_G Hirshfeld Test Diff (M-X)  Mn1      --O2       .        9.0 s.u.
PLAT242_ALERT_2_G Low    'MainMol' Ueq as Compared to Neighbors of        C14 Check
PLAT242_ALERT_2_G Low    'MainMol' Ueq as Compared to Neighbors of        C28 Check
PLAT300_ALERT_4_G Atom Site Occupancy of F4         Constrained at        0.5 Check
PLAT300_ALERT_4_G Atom Site Occupancy of F5         Constrained at        0.5 Check
PLAT300_ALERT_4_G Atom Site Occupancy of F6         Constrained at        0.5 Check
PLAT300_ALERT_4_G Atom Site Occupancy of F7         Constrained at        0.5 Check
PLAT300_ALERT_4_G Atom Site Occupancy of F8         Constrained at        0.5 Check
PLAT300_ALERT_4_G Atom Site Occupancy of F9         Constrained at        0.5 Check
PLAT301_ALERT_3_G Main Residue  Disorder ..............(Resd  1  )         5% Note
PLAT434_ALERT_2_G Short Inter HL..HL Contact F3       ..F6               2.68 Ang.
                                          3-x,-1/2+y,1/2-z  =      3_845 Check
PLAT769_ALERT_4_G CIF Embedded explicitly supplied scattering data     Please Note
PLAT794_ALERT_5_G Tentative Bond Valency for Mn1       (II)      .       1.88 Info
PLAT808_ALERT_5_G No Parseable SHELXL Style Weighting Scheme Found     Please Check
PLAT860_ALERT_3_G Number of Least-Squares Restraints .............         13 Note
PLAT882_ALERT_1_G No Datum for _diffrn_reflns_av_unetI/netI ......     Please Do !
PLAT912_ALERT_4_G Missing # of FCF Reflections Above STh/L=  0.600         14 Note
PLAT929_ALERT_5_G No Weight Pars,Obs and Calc R1,wR2,S not Checked          ! Info
PLAT960_ALERT_3_G Number of Intensities with I  <   - 2*sig(I) ...          3 Check
PLAT992_ALERT_5_G Repd & Actual _reflns_number_gt Values Differ by          2 Check
```

```
   0 ALERT level A = Most likely a serious problem - resolve or explain
   0 ALERT level B = A potentially serious problem, consider carefully
   8 ALERT level C = Check. Ensure it is not caused by an omission or oversight
  22 ALERT level G = General information/check it is not something unexpected

   1 ALERT type 1 CIF construction/syntax error, inconsistent or missing data
  10 ALERT type 2 Indicator that the structure model may be wrong or deficient
   6 ALERT type 3 Indicator that the structure quality may be low
   8 ALERT type 4 Improvement, methodology, query or suggestion
   5 ALERT type 5 Informative message, check
```

It is advisable to attempt to resolve as many as possible of the alerts in all categories. Often the minor alerts point to easily fixed oversights, errors and omissions in your CIF or refinement strategy, so attention to these fine details can be worthwhile. In order to resolve some of the more serious problems it may be necessary to carry out additional measurements or structure refinements. However, the purpose of your study may justify the reported deviations and the more serious of these should normally be commented upon in the discussion or experimental section of a paper or in the "special_details" fields of the CIF. checkCIF was carefully designed to identify outliers and unusual parameters, but every test has its limitations and alerts that are not important in a particular case may appear. Conversely, the absence of alerts does not guarantee there are no aspects of the results needing attention. It is up to the individual to critically assess their own results and, if necessary, seek expert advice.

Publication of your CIF in IUCr journals

A basic structural check has been run on your CIF. These basic checks will be run on all CIFs submitted for publication in IUCr journals (*Acta Crystallographica*, *Journal of Applied Crystallography*, *Journal of Synchrotron Radiation*); however, if you intend to submit to *Acta Crystallographica Section C* or *E* or *IUCrData*, you should make sure that full publication checks are run on the final version of your CIF prior to submission.

Publication of your CIF in other journals

Please refer to the *Notes for Authors* of the relevant journal for any special instructions relating to CIF submission.

PLATON version of 05/12/2020; check.def file version of 05/12/2020

Datablock I - ellipsoid plot

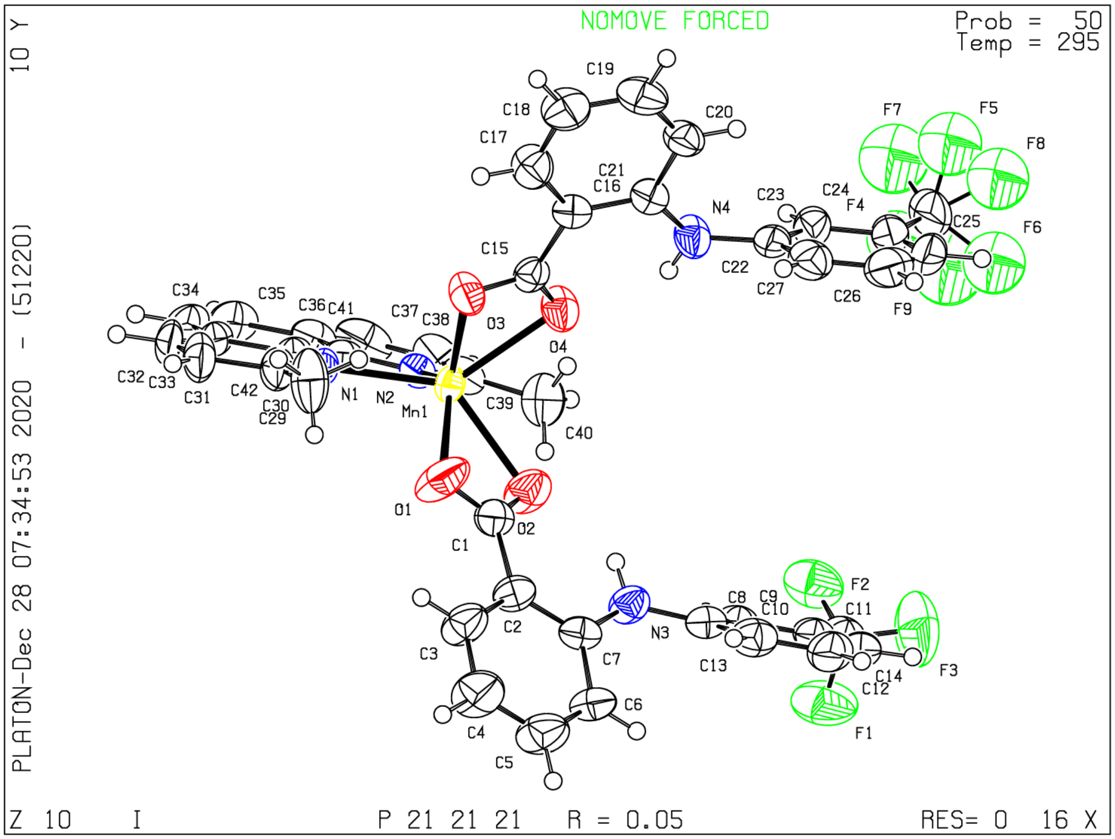

Supplement: Supplementary file 1 [file ijms-25-13457-s001.zip › Supplementary File S1/Checkcif of complex 7.pdf]
