# Supplementary material for: Manganese(II) Complexes with Non-Steroidal Anti-Inflammatory Drugs: Structure and Biological Activity
Source: Int J Mol Sci. 2024 Dec 16;25(24):13457. doi: 10.3390/ijms252413457 (PMC11676910; doi:10.3390/ijms252413457)

checkCIF/PLATON report

Structure factors have been supplied for datablock(s) I

THIS REPORT IS FOR GUIDANCE ONLY. IF USED AS PART OF A REVIEW PROCEDURE FOR PUBLICATION, IT SHOULD NOT REPLACE THE EXPERTISE OF AN EXPERIENCED CRYSTALLOGRAPHIC REFEREE.

No syntax errors found. CIF dictionary Interpreting this report

Datablock: I

Bond precision: C-C = 0.0039 A Wavelength=0.71073

Cell:	a=11.8346(6)	b=13.2879(6)	c=13.3701(6)
	alpha=112.102(2)	beta=94.981(2)	gamma=114.137(2)
Temperature:	295 K		

	Calculated	Reported
Volume	1704.17(15)	1704.17(15)
Space group	P -1	P -1
Hall group	-P 1	?
Moiety formula	2(C40 H26 F4 Mn N2 O6), 0.25(C2 O2), 0.5(C O)	C40.50 H26 F4 Mn1 N2 O6.50
Sum formula	C81 H52 F8 Mn2 N4 O13	C40.50 H26 F4 Mn1 N2 O6.50
Mr	1551.15	775.58
Dx,g cm-3	1.511	1.511
Z	1	2
Mu (mm-1)	0.465	0.465
F000	792.0	792.0
F000’	793.12	
h,k,lmax	14,16,16	14,16,16
Nref	6619	6542
Tmin,Tmax	0.915,0.937	0.930,0.940
Tmin’	0.874	

Correction method= # Reported T Limits: Tmin=0.930 Tmax=0.940
AbsCorr = NUMERICAL

Data completeness= 0.988 Theta(max)= 25.872

R(reflections)= 0.0420(4994) wR2(reflections)= 0.0846(4994)

S = 1.000 Npar= 494

The following ALERTS were generated. Each ALERT has the format
test-name_ALERT_alert-type_alert-level.
Click on the hyperlinks for more details of the test.

Alert level C

```
PLAT241_ALERT_2_C High   'MainMol' Ueq as Compared to Neighbors of         O5 Check
PLAT911_ALERT_3_C Missing FCF Refl Between Thmin & STh/L=    0.600         26 Report
```

Alert level G

```
PLAT003_ALERT_2_G Number of Uiso or Uij Restrained non-H Atoms ...          4 Report
PLAT007_ALERT_5_G Number of Unrefined Donor-H Atoms .............          2 Report
PLAT042_ALERT_1_G Calc. and Reported MoietyFormula Strings  Differ     Please Check
PLAT045_ALERT_1_G Calculated and Reported Z Differ by a Factor ...       0.50 Check
PLAT154_ALERT_1_G The s.u.'s on the Cell Angles are Equal ..(Note)      0.002 Degree
PLAT232_ALERT_2_G Hirshfeld Test Diff (M-X)  Mn1      --O2       .        5.4 s.u.
PLAT300_ALERT_4_G Atom Site Occupancy of O8         Constrained at       0.25 Check
PLAT300_ALERT_4_G Atom Site Occupancy of C42        Constrained at       0.25 Check
PLAT300_ALERT_4_G Atom Site Occupancy of O7         Constrained at       0.25 Check
PLAT300_ALERT_4_G Atom Site Occupancy of C41        Constrained at       0.25 Check
PLAT302_ALERT_4_G Anion/Solvent/Minor-Residue Disorder (Resd  2  )       100% Note
PLAT302_ALERT_4_G Anion/Solvent/Minor-Residue Disorder (Resd  3  )       100% Note
PLAT304_ALERT_4_G Non-Integer Number of Atoms in ..... (Resd  3  )       0.50 Check
PLAT315_ALERT_2_G Singly Bonded Carbon Detected (H-atoms Missing).        C41 Check
PLAT769_ALERT_4_G CIF Embedded explicitly supplied scattering data     Please Note
PLAT794_ALERT_5_G Tentative Bond Valency for Mn1       (II)      .       1.85 Info
PLAT808_ALERT_5_G No Parseable SHELXL Style Weighting Scheme Found     Please Check
PLAT860_ALERT_3_G Number of Least-Squares Restraints ............         309 Note
PLAT882_ALERT_1_G No Datum for _diffrn_reflns_av_unetI/netI ......     Please Do !
PLAT910_ALERT_3_G Missing # of FCF Reflection(s) Below Theta(Min).          1 Note
PLAT912_ALERT_4_G Missing # of FCF Reflections Above STh/L=  0.600         72 Note
PLAT929_ALERT_5_G No Weight Pars,Obs and Calc R1,wR2,S not Checked          ! Info
PLAT960_ALERT_3_G Number of Intensities with I  <   - 2*sig(I) ...        180 Check
PLAT992_ALERT_5_G Repd & Actual _reflns_number_gt Values Differ by          2 Check
```

---

0 **ALERT level A** = Most likely a serious problem - resolve or explain
0 **ALERT level B** = A potentially serious problem, consider carefully
2 **ALERT level C** = Check. Ensure it is not caused by an omission or oversight
24 **ALERT level G** = General information/check it is not something unexpected

4 ALERT type 1 CIF construction/syntax error, inconsistent or missing data
4 ALERT type 2 Indicator that the structure model may be wrong or deficient
4 ALERT type 3 Indicator that the structure quality may be low
9 ALERT type 4 Improvement, methodology, query or suggestion
5 ALERT type 5 Informative message, check

It is advisable to attempt to resolve as many as possible of the alerts in all categories. Often the minor alerts point to easily fixed oversights, errors and omissions in your CIF or refinement strategy, so attention to these fine details can be worthwhile. In order to resolve some of the more serious problems it may be necessary to carry out additional measurements or structure refinements. However, the purpose of your study may justify the reported deviations and the more serious of these should normally be commented upon in the discussion or experimental section of a paper or in the "special_details" fields of the CIF. checkCIF was carefully designed to identify outliers and unusual parameters, but every test has its limitations and alerts that are not important in a particular case may appear. Conversely, the absence of alerts does not guarantee there are no aspects of the results needing attention. It is up to the individual to critically assess their own results and, if necessary, seek expert advice.

Publication of your CIF in IUCr journals

A basic structural check has been run on your CIF. These basic checks will be run on all CIFs submitted for publication in IUCr journals (*Acta Crystallographica*, *Journal of Applied Crystallography*, *Journal of Synchrotron Radiation*); however, if you intend to submit to *Acta Crystallographica Section C* or *E* or *IUCrData*, you should make sure that full publication checks are run on the final version of your CIF prior to submission.

Publication of your CIF in other journals

Please refer to the *Notes for Authors* of the relevant journal for any special instructions relating to CIF submission.

PLATON version of 05/12/2020; check.def file version of 05/12/2020

Datablock I - ellipsoid plot

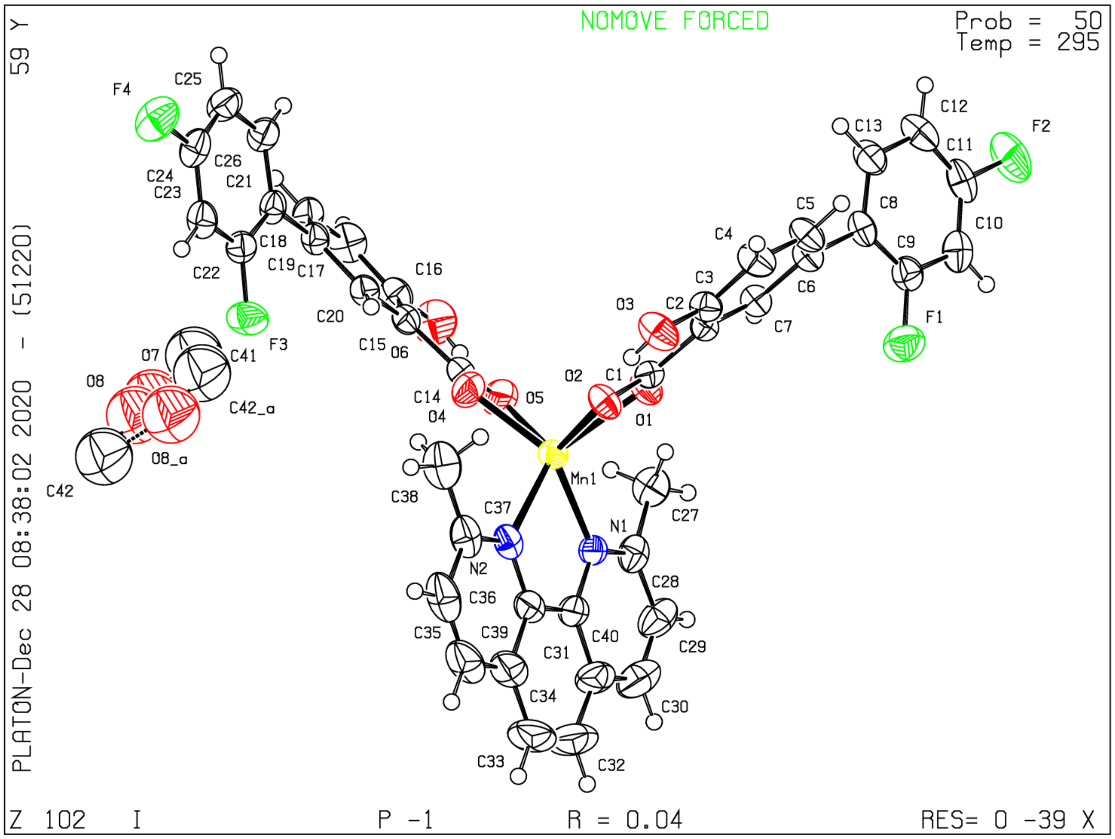

Supplement: Supplementary file 1 [file ijms-25-13457-s001.zip › Supplementary File S1/Checkcif of complex 9.pdf]
