# Supplementary material for: Manganese(II) Complexes with Non-Steroidal Anti-Inflammatory Drugs: Structure and Biological Activity
Source: Int J Mol Sci. 2024 Dec 16;25(24):13457. doi: 10.3390/ijms252413457 (PMC11676910; doi:10.3390/ijms252413457)
Supplement: Supplementary file 1 [file ijms-25-13457-s001.zip › Supplementary Figures and Tables.pdf]

Manganese(II) complexes with non-steroidal anti-inflammatory drugs: Structure, and biological activity

Filitsa Dimiza, Antonios G. Hatzidimitriou, George Psomas *

Department of General and Inorganic Chemistry, Faculty of Chemistry, Aristotle University of Thessaloniki, GR-54124 Thessaloniki, Greece

SUPPLEMENTARY MATERIAL

*Corresponding author's e-mail: gepsomas@chem.auth.gr

Content

EXPERIMENTAL PROTOCOLS 4

S1 Antioxidant activity assay 4

S2 Binding studies with CT DNA 4

S2.1 Binding study with CT DNA by UV−vis spectroscopy 5

S1.2 CT DNA−binding studies by viscosity measurements 5

S2.3 EB−displacement studies 6

S3 Albumin-binding studies 6

S4 References 8

TABLES 9

Table S1. Experimental crystallographic details for complexes **1** and **2**. 9

Table S2. Experimental crystallographic details for complexes **3** and **4**. 10

Table S3. Experimental crystallographic details for complexes **5** and **6**. 11

Table S4. Experimental crystallographic details for complexes **7** and **8**. 12

Table S5. Experimental crystallographic details for complex **9** 13

Table S6. Selected bond distances (Å) and angles (º) for complex **1** 14

Table S7. Selected bond distances (Å) and angles (º) for complex **2** 15

Table S8. Hydrogen bonds (lengths in Å, angles in °) for complexes **1-4**. 16

Table S9. Selected bond distances (Å) and angles (º) for complexes **3** and **4**. 17

Table S10. Selected bond distances (Å) and angles (º) for complex **5** (molecules **5A** and **5B**) 18

Table S11. Selected bond distances (Å) and angles (º) for complex **6** (molecules **6A** and **6B**) 19

Table S12. Selected bond distances (Å) and angles (º) for complexes **7** and **8**. 20

Table S13. Hydrogen bonds (lengths in Å, angles in °) for complexes **5-9**. 21

Table S14. Selected bond distances (Å) and angles (º) for complex **9** 22

FIGURES 23

Figure S1. IR spectra (KBr pellets) of complexes **1** and **2** 23

Figure S2. IR spectra (KBr pellets) of complexes **3** and **4** 24

Figure S3. IR spectra (KBr pellets) of complexes **5, 6** and **7** 25

Figure S4. IR spectra (KBr pellets) of complexes **8** and **9** 26

Figure S5. Molecular structure of complexes (A) **5B** and (B) **6B**. 27

Figure S6. The UV−vis spectra of a CT DNA solution in buffer (containing 150 mM NaCl and 15 mM trisodium citrate at pH 7.0) recorded in the presence of increasing amounts of the complexes. 28

Figure S7. UV−vis spectra of a DMSO solution of the complexes in the presence of increasing amounts of CT DNA. 30

Figure S8. Plots of $\frac{[DNA]}{(\varepsilon_A - \varepsilon_f)}$ *versus* [DNA] for the complexes. 32

Figure S9. Fluorescence emission spectra ($\lambda_{excitation}$ = 540 nm) for EB-DNA in buffer solution in the absence and presence of increasing amounts of the complexes. 34

Figure S10. Stern−Volmer plots of the EB−DNA quenching experiments upon addition of the complexes. 36

Figure S11. Fluorescence emission spectra ($\lambda_{excitation}$ = 295 nm) of a buffer solution (150 mM NaCl and 15 mM trisodium citrate at pH 7.0) of HSA (3 μM) in the presence of increasing amounts of the complexes. 38

Figure S12. Fluorescence emission spectra ($\lambda_{excitation}$ = 295 nm) of a buffer solution (150 mM NaCl and 15 mM trisodium citrate at pH 7.0) of BSA (3 μM) in the presence of increasing amounts of the complexes. 39

Figure S13. Stern−Volmer plots of the HSA quenching experiments upon addition of the complexes. 40

Figure S14. Stern−Volmer plots of the BSA quenching experiments upon addition of the complexes. 42

Figure S15. Scatchard plots of the HSA quenching experiments upon addition of the complexes. 44

Figure S16. Scatchard plots of the BSA quenching experiments upon addition of the complexes. 46

EXPERIMENTAL PROTOCOLS

S1 Antioxidant activity assay

The antioxidant activity of the compounds was evaluated *via* the ability to scavenge *in vitro* ABTS radicals. The experiments were carried out in triplicate and the standard deviation of absorbance was less than 10% of the mean.

The ABTS assay was performed to determine the activity of the compounds to scavenge the radical cation ABTS. Initially, a water solution of ABTS was prepared (2 mM). ABTS radical cation ($ABTS^{+\cdot}$) was produced by the reaction of ABTS stock solution with potassium persulfate (0.17 mM) and the mixture was stored in the dark at room temperature for 12−16 h before its use. The ABTS was oxidized incompletely because the stoichiometric reaction ratio of ABTS and potassium persulfate is 1:0.5. The absorbance became maximal and stable only after more than 6 h of reaction, although the oxidation of the ABTS started immediately. The radical was stable in this form for more than 2 days when allowed to stand in the dark at room temperature. Afterwards, the $ABTS^{+\cdot}$ solution was diluted in ethanol to an absorbance of 0.70 at 734 nm and 10 μL of diluted compound or standards (0.1 mM) in DMSO were added. The absorbance was recorded out exactly 1 min after initial mixing.[1] The ABTS radical scavenging activity was expressed as the percentage inhibition of the absorbance of the initial ABTS solution (ABTS%). Trolox was used as an appropriate standard.

S2 Binding studies with CT DNA

In order to study the interaction of the complex with DNA, the compound was initially dissolved in DMSO (1 mM). Mixing of such solutions with the aqueous buffer solutions DNA used in the studies never exceeded 5% DMSO (v/v) in the final solution, which was needed due to low aqueous solubility of most compounds. In all experiments, the effect of DMSO on the data has been taken into consideration and the appropriate

corrections have been performed. The interaction of the compound with CT DNA was monitored by UV−vis spectroscopy, and viscosity measurements, and *via* competitive studies with EB by fluorescence emission spectroscopy.

S2.1 Binding study with CT DNA by UV−vis spectroscopy

The interaction of the compound with CT DNA has been studied by UV−vis spectroscopy as a means to initially investigate the possible binding mode to CT DNA and to calculate the DNA−binding constant (K_b).

The UV−vis spectra of a CT DNA (0.13–0.24 mM) solution in buffer (containing 150 mM NaCl and 15 mM trisodium citrate at pH 7.0) were recorded in the presence of each compound at diverse [compound]/[DNA] mixing ratios (= *r*). Control experiments with DMSO were performed and no changes in the spectra of CT DNA were observed.

The K_b constant (in M^{-1}) of the compounds was determined by the Wolfe−Shimer equation (equation S1) [2] and the plots $[DNA]/(\varepsilon_A-\varepsilon_f)$ *versus* [DNA] using the UV−vis spectra of the compound (10–50 μM) recorded for a constant concentration with increasing amounts of CT DNA for diverse [compound]/[DNA] mixing ratios (= *r*). According to the Wolfe−Shimer equation (equation S1):

$$\frac{[DNA]}{(\varepsilon_A-\varepsilon_f)}=\frac{[DNA]}{(\varepsilon_b-\varepsilon_f)}+\frac{1}{K_b(\varepsilon_b-\varepsilon_f)} \qquad \text{(equation S1)}$$

where [DNA] is the concentration of DNA in base pairs, $\varepsilon_A = A_{obsd}/[compound]$, ε_f = the extinction coefficient for the free compound and ε_b = the extinction coefficient for the compound in the fully bound form. K_b is given by the ratio of slope to the y intercept in plots $[DNA]/(\varepsilon_A-\varepsilon_f)$ *versus* [DNA].

S1.2 CT DNA−binding studies by viscosity measurements

The interaction of compound with DNA was evaluated *via* the study of the CT DNA viscosity ([DNA] = 0.1 mM) in a buffer solution (150 mM NaCl and 15 mM trisodium citrate at pH 7.0) in the presence of increasing amounts of the compound (up to the value of *r* = 0.36). The obtained data are presented as $(\eta/\eta_0)^{1/3}$ *versus* *r*, where η is the viscosity of DNA in the presence of the compound, and η_0 is the viscosity of DNA alone in buffer solution.

S2.3 EB−displacement studies

The competition of the complex with EB was investigated by fluorescence emission spectroscopy to examine whether the compound can displace EB from its DNA−EB adduct. The CT DNA−EB complex was formed by pre−treating 40 μM EB and 45 μM CT DNA in buffer (150 mM NaCl and 15 mM trisodium citrate at pH 7.0). The possible displacement of EB by the compound and subsequently the intercalating effect was studied by the stepwise addition of a certain amount of the solution of each compound into the solution of the CT DNA−EB adduct. The solutions were excited at 540 nm and the emission was monitored from 550−700 nm with λ_{max} = 592−594 nm and the effect of the addition of the compound to the CT−DNA EB solution was recorded. The compound did not display any fluorescence emission bands at room temperature in solution or in the presence of CT DNA or EB under the same experimental conditions ($\lambda_{excitation}$ = 540 nm); therefore, the observed quenching of the EB−DNA solution may be attributed to the displacement of EB from its EB−DNA adduct.

The Stern−Volmer constants (K_{SV}, in M^{-1}) were calculated according by the linear Stern−Volmer equation (equation S2) [3] and the respective plots Io/I *versus* [compound]:

$$\frac{Io}{I} = 1 + k_q\tau_0[Q] = 1 + K_{SV}[Q] \qquad \text{(equation S2)}$$

where Io and I are the emission intensities of the EB−DNA solution in the absence and the presence of the compound, respectively, τ_o = the average lifetime of the emitting system without the quencher and k_q = the quenching constant. Taking τ_o = 23 ns as the fluorescence lifetime of the EB−DNA adduct,[4] the quenching constant (k_q, in $M^{-1}s^{-1}$) of the compound was calculated according to equation S3:[3]

$$K_{SV} = k_q\tau_o \qquad \text{(equation S3)}$$

S3 Albumin-binding studies

As a means to investigate if the compound can bind to carrier protein like serum albumins, albumin binding studies were carried out by tryptophan fluorescence quenching experiments using BSA or HSA (3 μM) in buffer (containing 15 mM trisodium citrate and

150 mM NaCl at pH 7.0). The quenching of the emission intensity of tryptophan residues of BSA at 345 nm or HSA at 340 nm was monitored using the compounds as quenchers with increasing concentration [3]. The fluorescence emission spectra of the compound were also recorded with λ_{ex} = 295 nm; in case that an additional emission band appeared the BSA−fluorescence emission spectra were corrected by subtracting the spectra of the compound. The influence of the inner−filter effect on the measurements was evaluated by equation S4: [5]

$$I_{corr} = I_{meas} \times 10^{\frac{\varepsilon(\lambda_{exc})cd}{2}} \times 10^{\frac{\varepsilon(\lambda_{em})cd}{2}} \qquad \text{(equation S4)}$$

where I_{corr} = corrected intensity, I_{meas} = the measured intensity, c = the concentration of the quencher, d = the cuvette (1 cm), $\varepsilon_{(\lambda exc)}$ and $\varepsilon_{(\lambda em)}$ = the ε of the quencher at the excitation and the emission wavelength, respectively, as calculated from the UV−vis spectra of the compound [5].

The Stern−Volmer and Scatchard graphs are used to study the interaction of the compound with the albumins. According to Stern−Volmer quenching equation (equation S2), where Io = initial tryptophan fluorescence intensity of the albumin, I = tryptophan fluorescence intensity of the albumin after the addition of the quencher, k_q = quenching constant, K_{SV} = Stern−Volmer constant, τ_o = average lifetime of the albumin without the quencher, and, taking as fluorescence lifetime (τ_o) of tryptophan in the albumin at around 10^{-8} s,[3] K_{SV} (in M^{-1}) can be obtained by the slope of the diagram Io/I *versus* [compound] (Stern−Volmer plots), and subsequently the quenching constant (k_q, in $M^{-1}s^{-1}$) may be calculated from equation S3.

From the Scatchard equation (equation S5):

$$\frac{\Delta I / Io}{[Q]} = nK - K\frac{\Delta I}{Io} \qquad \text{(equation S5)}$$

where n is the number of binding sites per albumin and K is the albumin−binding constant (K, in M^{-1}) is calculated from the slope in plots (ΔI/Io)/[compound] *versus* (ΔI/Io) and n is given by the ratio of y intercept to the slope [6].

S4 References

1. Kontogiorgis, C.; Hadjipavlou-Litina, D. Biological Evaluation of Several Coumarin Derivatives Designed as Possible Anti-Inflammatory/Antioxidant Agents. *J Enzyme Inhib Med Chem* **2003**, *18*, 63–69, doi:10.1080/1475636031000069291.
2. Wolfe, A.; Shimer, G.H.; Meehan, T. Polycyclic Aromatic Hydrocarbons Physically Intercalate into Duplex Regions of Denatured DNA. *Biochemistry* **1987**, *26*, 6392–6396, doi:10.1021/bi00394a013.
3. Lakowicz, J.R. *Principles of Fluorescence Spectroscopy*; Springer, 2006; ISBN 0387312781.
4. Heller, D.P.; Greenstock, C.L. Fluorescence Lifetime Analysis of DNA Intercalated Ethidium Bromide and Quenching by Free Dye. *Biophys Chem* **1994**, *50*, 305–312, doi:10.1016/0301-4622(93)E0101-A.
5. Stella, L.; Capodilupo, A.L.; Bietti, M. A Reassessment of the Association between Azulene and [60]Fullerene. Possible Pitfalls in the Determination of Binding Constants through Fluorescence Spectroscopy. *Chemical Communications* **2008**, 4744–4746, doi:10.1039/b808357f.
6. Wang, Y.-Q.; Zhang, H.-M.; Zhang, G.-C.; Tao, W.-H.; Tang, S.-H. Interaction of the Flavonoid Hesperidin with Bovine Serum Albumin: A Fluorescence Quenching Study. *J Lumin* **2007**, *126*, 211–218, doi:10.1016/J.JLUMIN.2006.06.013.

TABLES

Table S1. Experimental crystallographic details for complexes **1** and **2**.

	Complex **1**	Complex **2**
Crystal data		
CCDC no	2404798	2404799
Chemical formula	$C_{48}H_{44}Cl_4MnN_6O_6$	$C_{48.50}H_{51.50}MnN_{5.50}O_6$
M_r	997.65	862.40
Crystal system, space group	Triclinic, *P*-1	Triclinic, *P*-1
Temperature (K)	295	295
a, *b*, *c* (Å)	10.0646(7), 10.3148(8), 13.1670(11)	7.856 (3), 16.522 (7), 18.982 (8)
α, β, γ (°)	111.773 (4), 91.704 (4), 109.282 (4)	115.14 (2), 91.68 (2), 100.24 (2)
V (Å^3)	1180.00 (17)	2179.4 (17)
Z	1	2
Radiation type	Mo *K*α	Mo *K*α
μ (mm^{-1})	0.56	0.36
Crystal size (mm)	0.30 × 0.09 × 0.06	0.27 × 0.12 × 0.10
Data collection		
Diffractometer	Bruker Kappa Apex2	
Absorption correction	Numerical<br>Analytical Absorption (De Meulenaer & Tompa, 1965)	
T_{min}, T_{max}	0.95, 0.97	0.96, 0.96
No. of measured reflections	24612	58323
No. of independent reflections	4573	8799
No. of observed [$I > 2.0\sigma(I)$] reflections	3198	6452
R_{int}	0.031	0.055
$(\sin \theta/\lambda)_{max}$ (Å^{-1})	0.615	0.622
Refinement		
$R[F^2 > 2\sigma(F^2)]$, $wR(F^2)$, S	0.048, 0.068, 1.00	0.049, 0.066, 1.00
No. of reflections	3198	6452
No. of parameters	295	547
H-atom treatment	H-atom parameters constrained	
$\Delta\rho_{max}$, $\Delta\rho_{min}$ (e Å^{-3})	0.68, −0.49	0.44, −0.32

Table S2. Experimental crystallographic details for complexes **3** and **4**.

	Complex **3**	Complex **4**
Crystal data		
CCDC no	2404800	2404801
Chemical formula	$C_{42}H_{36}Cl_4MnN_4O_6$	$C_{40}H_{36}Cl_4MnN_4O_6$
M_r	889.51	865.49
Crystal system, space group	Monoclinic, *C*2/*c*	Monoclinic, *C*2/*c*
Temperature (K)	295	200
a, *b*, *c* (Å)	29.802(4), 10.4108(14), 13.4826(16)	29.6385(16), 9.7115(5), 13.9553(8)
β (°)	96.699 (3)	97.229 (2)
V (Å^3)	4154.6 (9)	3984.9 (4)
Z	4	4
Radiation type	Mo *Kα*	Mo *Kα*
μ (mm^{-1})	0.63	0.65
Crystal size (mm)	0.24 × 0.12 × 0.09	0.22 × 0.13 × 0.11
Data collection		
Diffractometer	Bruker Kappa Apex2	
Absorption correction	Numerical<br>Analytical Absorption (De Meulenaer & Tompa, 1965)	
T_{min}, T_{max}	0.93, 0.95	0.92, 0.93
No. of measured reflections	28681	16420
No. of independent reflections	3973	3783
No. of observed [$I > 2.0\sigma(I)$] reflections	2697	2766
R_{int}	0.023	0.010
$(\sin \theta/\lambda)_{max}$ (Å^{-1})	0.612	0.612
Refinement		
$R[F^2 > 2\sigma(F^2)]$, $wR(F^2)$, S	0.051, 0.081, 1.00	0.041, 0.064, 1.00
No. of reflections	2697	2766
No. of parameters	253	249
H-atom treatment	H-atom parameters constrained	
$\Delta\rho_{max}$, $\Delta\rho_{min}$ (e Å^{-3})	1.25, −1.38	0.70, −0.39

Table S3. Experimental crystallographic details for complexes **5** and **6**.

	Complex **5**	Complex **6**
**Crystal data**		
CCDC no	2404802	2404803
Chemical formula	$C_{84}H_{65}Cl_8Mn_2N_8O_{8.50}$	$C_{91}H_{93}Mn_2N_8O_{11.50}$
M_r	1715.97	1592.64
Crystal system, space group	Triclinic, *P*-1	Triclinic, *P*-1
Temperature (K)	295	140
a, *b*, *c* (Å)	12.1911(7), 15.3887(8), 23.1861(10)	13.7438(14), 15.908(2), 20.007(3)
α, β, γ (°)	109.331 (2), 92.573 (2), 108.898 (3)	71.419 (7), 87.773 (6), 84.475 (6)
V (Å^3)	3826.6 (4)	4126.7 (9)
Z	2	2
Radiation type	Mo *K*α	Mo *K*α
μ (mm^{-1})	0.67	0.37
Crystal size (mm)	0.26 × 0.16 × 0.14	0.26 × 0.15 × 0.10
**Data collection**		
Diffractometer	Bruker Kappa Apex2	
Absorption correction	Numerical<br>Analytical Absorption (De Meulenaer & Tompa, 1965)	
T_{min}, T_{max}	0.90, 0.91	0.95, 0.96
No. of measured reflections	65210	61549
No. of independent reflections	14590	15904
No. of observed [$I > 2.0\sigma(I)$] reflections	10015	10630
R_{int}	0.069	0.014
(sin θ/λ)$_{max}$ (Å^{-1})	0.612	0.618
**Refinement**		
$R[F^2 > 2\sigma(F^2)]$, $wR(F^2)$, S	0.049, 0.080, 1.00	0.053, 0.117, 1.00
No. of reflections	10015	10630
No. of parameters	1000	1007
No. of restraints		6
H-atom treatment	H-atom parameters constrained	
$\Delta\rho_{max}$, $\Delta\rho_{min}$ (e Å^{-3})	0.37, −0.49	0.39, −1.67

Table S4. Experimental crystallographic details for complexes **7** and **8**.

	Complex **7**	Complex **8**
Crystal data		
CCDC no	2404804	2404805
Chemical formula	$C_{42}H_{30}F_6MnN_4O_4$	$C_{42}H_{34}Cl_2MnN_4O_4$
M_r	823.64	784.59
Crystal system, space group	Orthorhombic, $P2_12_12_1$	Monoclinic, $P2_1/c$
Temperature (K)	295	295
a, b, c (Å)	9.780 (6), 13.108 (8), 29.107 (17)	11.324 (3), 21.360 (6), 14.989 (3)
β (°)	90	93.057 (8)
V (Å^3)	3731 (4)	3620.6 (15)
Z	4	4
Radiation type	Mo $K\alpha$	Mo $K\alpha$
μ (mm^{-1})	0.43	0.56
Crystal size (mm)	0.25 × 0.15 × 0.14	0.25 × 0.22 × 0.11
Data collection		
Diffractometer	Bruker Kappa Apex2	
Absorption correction	Numerical<br>Analytical Absorption (De Meulenaer & Tompa, 1965)	
T_{min}, T_{max}	0.94, 0.94	0.88, 0.94
No. of measured reflections	18223	42288
No. of independent reflections	7040	6935
No. of observed [$I > 2.0\sigma(I)$] reflections	5511	4939
R_{int}	0.035	0.039
(sin θ/λ)$_{max}$ (Å^{-1})	0.611	0.613
Refinement		
$R[F^2 > 2\sigma(F^2)]$, $wR(F^2)$, S	0.053, 0.090, 1.00	0.038, 0.053, 1.00
No. of reflections	5511	4939
No. of parameters	512	478
No. of restraints	13	
H-atom treatment	H-atom parameters constrained	
$\Delta\rho_{max}$, $\Delta\rho_{min}$ (e Å^{-3})	0.52, −0.39	0.30, −0.35
Absolute structure	Flack (1983), 3064 Friedel-pairs	
Absolute structure parameter	0.03 (2)	

Table S5. Experimental crystallographic details for complex **9**.

	Complex **9**
Crystal data	
CCDC no	2404806
Chemical formula	$C_{40.50}H_{26}F_4MnN_2O_{6.50}$
M_r	775.58
Crystal system, space group	Triclinic, *P*-1
Temperature (K)	295
a, *b*, *c* (Å)	11.8346 (6), 13.2879 (6), 13.3701 (6)
α, β, γ (°)	112.102 (2), 94.981 (2), 114.137 (2)
V (Å^3)	1704.17 (15)
Z	2
Radiation type	Mo *K*α
μ (mm^{-1})	0.47
Crystal size (mm)	0.29 × 0.16 × 0.14
Data collection	
Diffractometer	Bruker Kappa Apex2
Absorption correction	Numerical<br>Analytical Absorption (De Meulenaer & Tompa, 1965)
T_{min}, T_{max}	0.93, 0.94
No. of measured reflections	31013
No. of independent reflections	6542
No. of observed [$I > 2.0\sigma(I)$] reflections	4994
R_{int}	0.032
(sin θ/λ)$_{max}$ (Å^{-1})	0.614
Refinement	
$R[F^2 > 2\sigma(F^2)]$, $wR(F^2)$, S	0.042, 0.076, 1.00
No. of reflections	4994
No. of parameters	494
No. of restraints	309
H-atom treatment	H-atom parameters constrained
$\Delta\rho_{max}$, $\Delta\rho_{min}$ (e Å^{-3})	0.34, −0.27

Table S6. Selected bond distances (Å) and angles (º) for complex **1**.

**Bond**	**Distance (Å)**	**Bond**	**Distance (Å)**
Mn1—O1	2.1554 (19)	Mn1—N1	2.325 (2)
Mn1—O3	2.200 (2)		
O1—C1	1.263 (3)	O2—C1	1.233 (3)
**Bonds**	**Angle (º)**	**Bonds**	**Angle (º)**
O1—Mn1—O3	89.19 (8)	O3^{i}—Mn1—O1	90.81 (8)
O1—Mn1—N1	91.64 (8)	N1^{i}—Mn1—O1	88.36 (8)
O3—Mn1—N1	89.78 (8)	N1^{i}—Mn1—O3	90.22 (8)

Symmetry code: (i) $-x+1$, $-y+1$, $-z+1$.

Table S7. Selected bond distances (Å) and angles (º) for complex **2**.

**Bond**	**Distance (Å)**	**Bond**	**Distance (Å)**
Mn1—O1	2.1215 (18)	Mn1—O6	2.1602 (19)
Mn1—O3	2.1501 (19)	Mn1—N1	2.280 (2)
Mn1—O5	2.2294 (19)	Mn1—N2	2.259 (2)
O1—C1	1.256 (3)	O3—C16	1.255 (3)
O2—C1	1.240 (3)	O4—C16	1.241 (3)
**Bonds**	**Angle (º)**	**Bonds**	**Angle (º)**
O1—Mn1—O3	173.00 (7)	O3—Mn1—N1	87.59 (8)
O1—Mn1—O5	95.74 (8)	O5—Mn1—N1	85.56 (9)
O3—Mn1—O5	88.28 (8)	O6—Mn1—N1	94.74 (9)
O1—Mn1—O6	88.50 (8)	O1—Mn1—N2	91.86 (9)
O3—Mn1—O6	87.50 (8)	O3—Mn1—N2	94.11 (8)
O5—Mn1—O6	175.75 (7)	O5—Mn1—N2	86.48 (8)
O1—Mn1—N1	87.02 (9)	O6—Mn1—N2	93.34 (9)
N1—Mn1—N2	171.80 (9)		

Table S8. Hydrogen bonds (lengths in Å, angles in °) for complexes **1-4**.

D*—H···*A	***D*—H (Å)**	**H···*A* (Å)**	***D*···*A* (Å)**	***D*—H···*A* (º)**	**Symmetry code**
Complex **1**					
O3—H32···N3	0.81	2.05	2.824 (5)	162	
O3—H242···O2	0.82	1.86	2.660 (5)	165	
N2—H21···O1	0.85	1.98	2.635 (5)	133	
Complex **2**					
O6—H62···O2	0.82	1.98	2.612 (2)	133	
O5—H51···O4	0.85	1.83	2.624 (6)	154	
N4—H41···O4	0.87	1.94	2.628 (6)	134	
O6—H61···N5	0.83	2.01	2.843 (6)	178	
N3—H515···O2	0.86	1.98	2.637 (6)	133	
Complex **3**					
N2—H21···O1	0.82	1.95	2.571 (6)	131	
O3—H214···O2^{i}	0.82	1.87	2.680 (6)	174	(i) $-x+1$, y, $-z+1/2$
Complex **4**					
N2—H21···O1	0.84	1.94	2.588 (4)	134	
O3—H204···O2	0.85	1.87	2.619 (4)	146	

Table S9. Selected bond distances (Å) and angles (º) for complexes **3** and **4**.

	Complex **3**	Complex **4**
**Bond**	**Distance (Å)**	**Distance (Å)**
Mn1—O1	2.133 (2)	2.1162 (16)
Mn1—O3	2.190 (3)	2.1849 (18)
Mn1—N1	2.259 (3)	2.283 (2)
O1—C1	1.270 (5)	1.267 (3)
O2—C1	1.240 (4)	1.236 (3)
**Bonds**	**Angle (º)**	**Angle (º)**
O1—Mn1—O3	88.00 (10)	82.50 (7)
O1—Mn1—N1	89.52 (10)	91.23 (7)
O3—Mn1—N1	93.23 (10)	165.06 (7)
$O3^{i}$—Mn1—O1	85.73 (9)	90.55 (7)
$N1^{i}$—Mn1—O1	98.27 (10)	97.44 (7)
$O1^{i}$—Mn1—O1	170.26 (14)	169.12 (10)
$O3^{i}$—Mn1—O3	99.87 (14)	100.76 (10)
$N1^{i}$—Mn1—O3	165.88 (10)	92.82 (8)
$N1^{i}$—Mn1—N1	74.28 (15)	74.48 (11)
Symmetry code	(i) $-x+1, y, -z+1/2$	(i) $-x+1, y, -z+1/2$

Table S10. Selected bond distances (Å) and angles (º) for complex **5** (molecules **5A** and **5B**).

	Molecule **5A**		Molecule **5B**
**Bond**	**Distance (Å)**	**Bond**	**Distance (Å)**
Mn1—O1	2.216 (3)	Mn2—O5	2.224 (3)
Mn1—O2	2.226 (3)	Mn2—O6	2.238 (3)
Mn1—O3	2.195 (3)	Mn2—O7	2.212 (3)
Mn1—O4	2.233 (3)	Mn2—O8	2.207 (3)
Mn1—N1	2.239 (3)	Mn2—N5	2.237 (3)
Mn1—N2	2.231 (3)	Mn2—N6	2.215 (3)
O1—C1	1.244 (4)	O5—C43	1.251 (4)
O2—C1	1.263 (4)	O6—C43	1.253 (5)
O3—C15	1.244 (4)	O7—C57	1.251 (4)
O4—C15	1.259 (4)	O8—C57	1.260 (4)
**Bonds**	**Angle (º)**	**Bonds**	**Angle (º)**
O1—Mn1—O2	58.51 (10)	O5—Mn2—O6	58.09 (10)
O1—Mn1—O3	98.28 (13)	O5—Mn2—O7	93.17 (12)
O2—Mn1—O3	102.12 (11)	O6—Mn2—O7	96.24 (11)
O1—Mn1—O4	102.86 (10)	O5—Mn2—O8	106.57 (11)
O2—Mn1—O4	153.20 (11)	O6—Mn2—O8	151.67 (11)
O3—Mn1—O4	58.90 (10)	O7—Mn2—O8	58.79 (10)
O1—Mn1—N1	102.01 (11)	O5—Mn2—N5	98.62 (11)
O2—Mn1—N1	113.92 (11)	O6—Mn2—N5	117.08 (11)
O3—Mn1—N1	143.90 (10)	O7—Mn2—N5	145.99 (10)
O4—Mn1—N1	87.45 (10)	O8—Mn2—N5	87.23 (10)
O1—Mn1—N2	149.97 (10)	O5—Mn2—N6	140.42 (11)
O2—Mn1—N2	94.59 (10)	O6—Mn2—N6	89.29 (10)
O3—Mn1—N2	100.56 (12)	O7—Mn2—N6	113.76 (11)
O4—Mn1—N2	106.86 (11)	O8—Mn2—N6	112.07 (11)
N1—Mn1—N2	75.41 (11)	N5—Mn2—N6	75.43 (11)

Table S11. Selected bond distances (Å) and angles (º) for complex **6** (molecules **6A** and **6B**).

	Molecule **6A**		Molecule **6B**
**Bond**	**Distance (Å)**	**Bond**	**Distance (Å)**
Mn1—N1	2.246 (3)	Mn2—N5	2.254 (3)
Mn1—N2	2.246 (3)	Mn2—N6	2.241 (3)
Mn1—O1	2.198 (2)	Mn2—O5	2.229 (2)
Mn1—O2	2.216 (2)	Mn2—O6	2.245 (2)
Mn1—O3	2.203 (2)	Mn2—O7	2.176 (2)
Mn1—O4	2.215 (2)	Mn2—O8	2.225 (2)
**Bonds**	**Angle (º)**	**Bonds**	**Angle (º)**
N1—Mn1—N2	74.95 (11)	N5—Mn2—N6	74.34 (10)
N1—Mn1—O1	136.57 (9)	N5—Mn2—O5	89.94 (9)
N2—Mn1—O1	102.01 (9)	N6—Mn2—O5	127.09 (9)
N1—Mn1—O2	82.85 (10)	N5—Mn2—O6	137.17 (9)
N2—Mn1—O2	114.56 (9)	N6—Mn2—O6	99.75 (10)
O1—Mn1—O2	58.55 (8)	O5—Mn2—O6	59.34 (8)
N1—Mn1—O3	102.77 (10)	N5—Mn2—O7	121.09 (9)
N2—Mn1—O3	145.61 (9)	N6—Mn2—O7	85.92 (9)
O1—Mn1—O3	102.21 (9)	O5—Mn2—O7	141.52 (9)
O2—Mn1—O3	98.86 (9)	O6—Mn2—O7	100.20 (8)
N1—Mn1—O4	123.07 (9)	N5—Mn2—O8	104.80 (10)
N2—Mn1—O4	92.07 (10)	N6—Mn2—O8	139.42 (9)
O1—Mn1—O4	100.19 (8)	O5—Mn2—O8	93.22 (9)
O2—Mn1—O4	147.93 (9)	O6—Mn2—O8	105.92 (9)
O3—Mn1—O4	59.81 (9)	O7—Mn2—O8	59.21 (9)

Table S12. Selected bond distances (Å) and angles (º) for complexes **7** and **8**.

	Complex **7**	Complex **8**
**Bond**	**Distance (Å)**	**Distance (Å)**
Mn1—O1	2.157 (4)	2.1850 (18)
Mn1—O2	2.343 (4)	2.2752 (16)
Mn1—O3	2.158 (3)	2.2085 (17)
Mn1—O4	2.349 (3)	2.2463 (16)
Mn1—N1	2.249 (3)	2.2322 (19)
Mn1—N2	2.237 (4)	2.2474 (19)
O1—C1	1.256 (6)	1.257 (3)
O2—C1	1.257 (6)	1.271 (3)
O3—C15	1.259 (5)	1.265 (3)
O4—C15	1.256 (5)	1.270 (3)
**Bonds**	**Angle (º)**	**Angle (º)**
O1—Mn1—O2	57.33 (13)	58.63 (6)
O1—Mn1—O3	99.99 (15)	91.37 (7)
O2—Mn1—O3	124.95 (14)	144.69 (6)
O1—Mn1—O4	119.13 (16)	85.49 (7)
O2—Mn1—O4	88.64 (13)	97.50 (6)
O3—Mn1—O4	57.50 (12)	58.90 (6)
O1—Mn1—N1	92.30 (15)	167.61 (7)
O2—Mn1—N1	133.52 (14)	109.13 (7)
O3—Mn1—N1	91.79 (13)	100.81 (7)
O4—Mn1—N1	137.72 (13)	98.84 (7)
O1—Mn1—N2	131.63 (16)	106.49 (7)
O2—Mn1—N2	98.78 (13)	112.07 (7)
O3—Mn1—N2	126.27 (14)	93.01 (6)
O4—Mn1—N2	99.29 (13)	150.22 (6)
N1—Mn1—N2	74.77 (13)	75.34 (7)

Table S13. Hydrogen bonds (lengths in Å, angles in °) for complexes **5-9**.

D—H···*A*	*D*—H (Å)	H···*A* (Å)	*D*···*A* (Å)	*D*—H···*A* (º)	Symmetry code
Complex **5**					
O9—H91···O5	0.82	2.11	2.931 (6)	180	
O9—H92···O5^{i}	0.82	1.88	2.696 (6)	179	(i) $-x$, $-y$, $-z$
N4—H41···O4	0.86	2.10	2.909 (6)	156	
N3—H31···O2	0.87	2.01	2.821 (6)	156	
N8—H81···O8	0.87	2.06	2.845 (6)	150	
N7—H825···O6	0.86	2.06	2.844 (6)	150	
Complex **6**					
N4—H41···O3	0.84	2.10	2.707 (7)	129	
N3—H31···O1	0.84	2.03	2.687 (7)	135	
N8—H81···O8	0.84	1.99	2.654 (7)	135	
N7—H71···O6	0.85	1.95	2.654 (7)	139	
O15—H950···O13	0.82	1.78	2.603 (7)	178	
O12—H942···O15	1.16	1.97	3.075 (7)	158	
O12—H948···O13^{ii}	0.82	1.88	2.697 (7)	178	(ii) $-x$+1, $-y$+1, $-z$+2
O14—H913···O12	0.84	1.93	2.584 (7)	134	
O14—H946···O13	0.95	2.38	3.293 (7)	161	
O13—H949···O14	0.95	2.50	3.293 (7)	141	
O9—H91···O1	0.82	2.15	2.906 (7)	154	
O9—H92···O3	0.82	2.06	2.830 (7)	156	
O11—H947···O10^{iii}	0.82	2.07	2.882 (7)	174	(iii) $-x$+1, $-y$, $-z$+2
O10—H101···O11	0.82	2.28	3.012 (7)	149	
Complex **7**					
N3—H31···O2	0.87	1.99	2.698 (8)	137	
N4—H405···O4	0.86	2.04	2.714 (8)	135	
Complex **8**					
N3—H31···O2	0.85	1.98	2.694 (4)	140	
N4—H41···O4	0.87	1.96	2.645 (4)	136	
Complex **9**					
O3—H31···O2	0.83	1.83	2.595 (5)	152	
O6—H61···O5	0.82	1.84	2.594 (5)	152	

Table S14. Selected bond distances (Å) and angles (º) for complex **9**.

**Bond**	**Distance (Å)**	**Bond**	**Distance (Å)**
Mn1—N1	2.2029 (19)	Mn1—O2	2.3130 (17)
Mn1—N2	2.2289 (19)	Mn1—O4	2.0357 (17)
Mn1—O1	2.1777 (16)		
**Bonds**	**Angle (º)**	**Bonds**	**Angle (º)**
N1—Mn1—N2	76.11 (7)	O1—Mn1—O2	58.26 (6)
N1—Mn1—O1	99.19 (7)	N1—Mn1—O4	135.46 (8)
N2—Mn1—O1	141.74 (7)	N2—Mn1—O4	102.53 (7)
N1—Mn1—O2	124.39 (7)	O1—Mn1—O4	106.41 (7)
N2—Mn1—O2	92.61 (6)	O2—Mn1—O4	100.11 (7)

FIGURES

Complex **1**	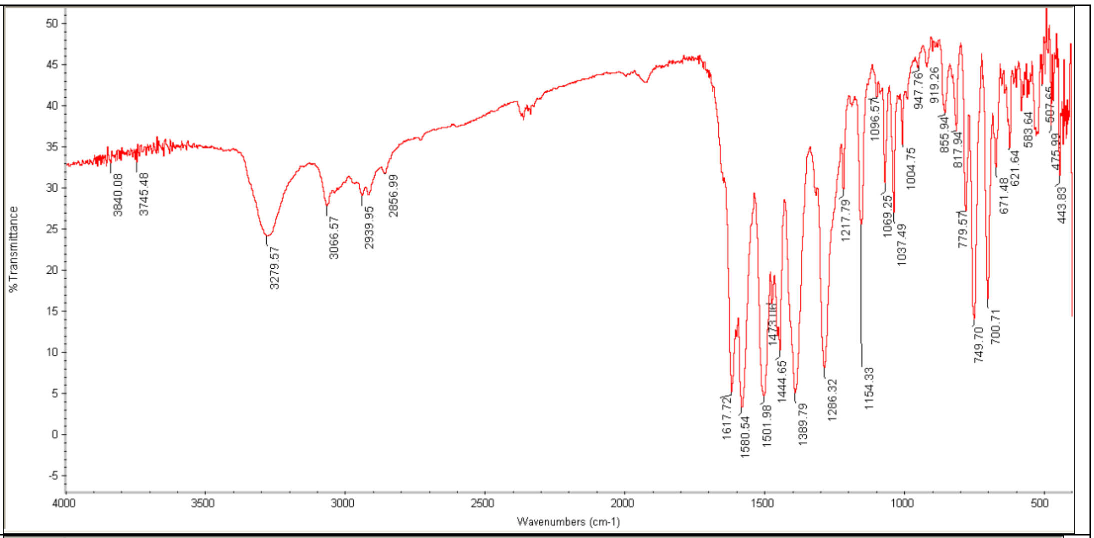
Complex **2**	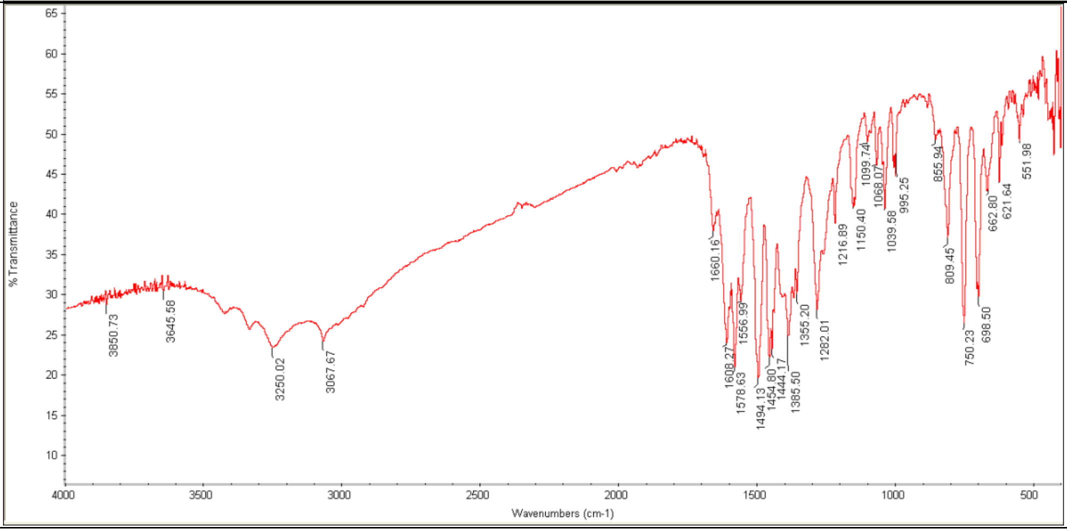

Figure S1. IR spectra (KBr pellets) of complexes **1** and **2**.

Complex **3**

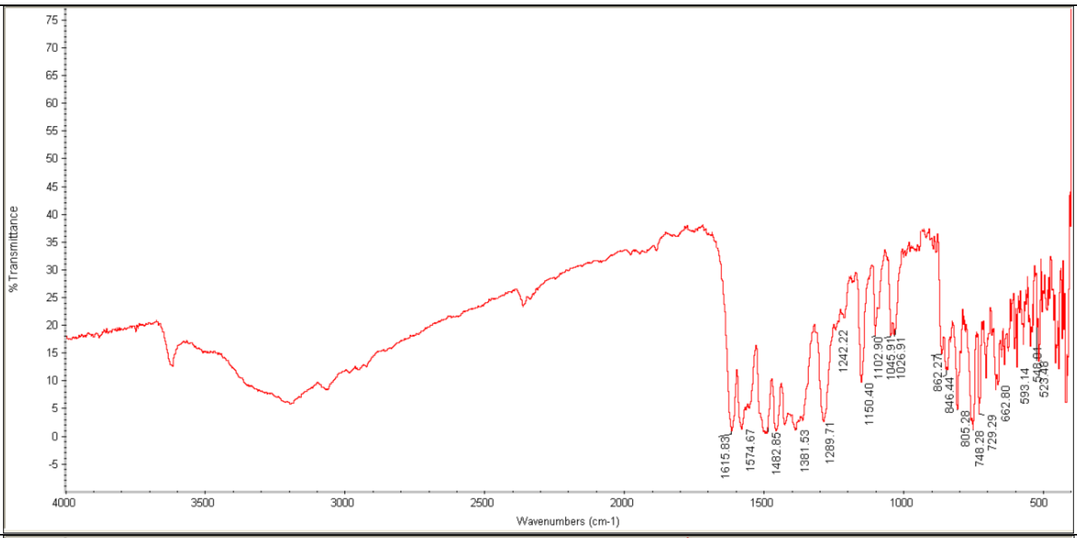

Complex **4**

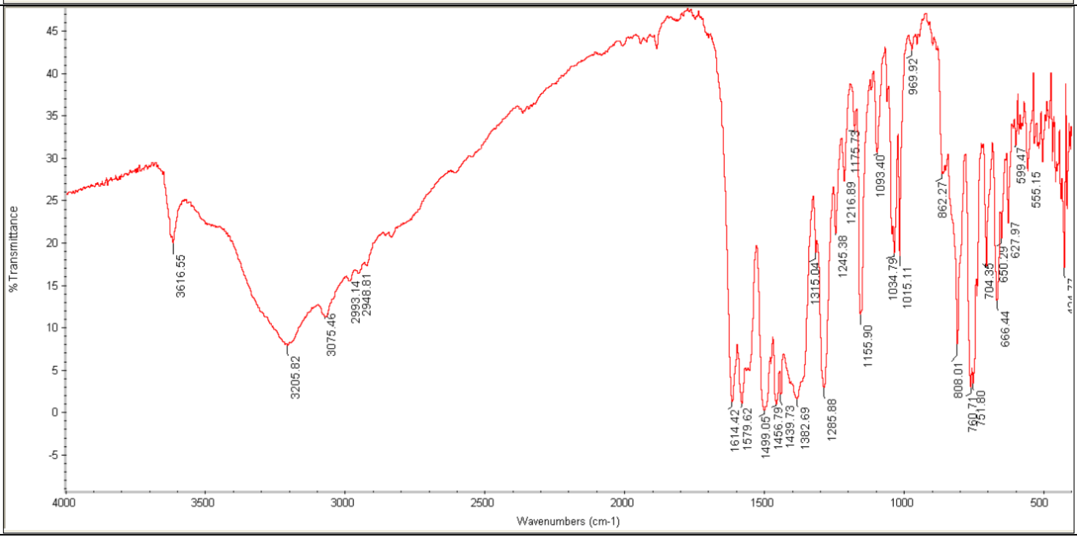

Figure S2. IR spectra (KBr pellets) of complexes **3** and **4**.

Complex **5**

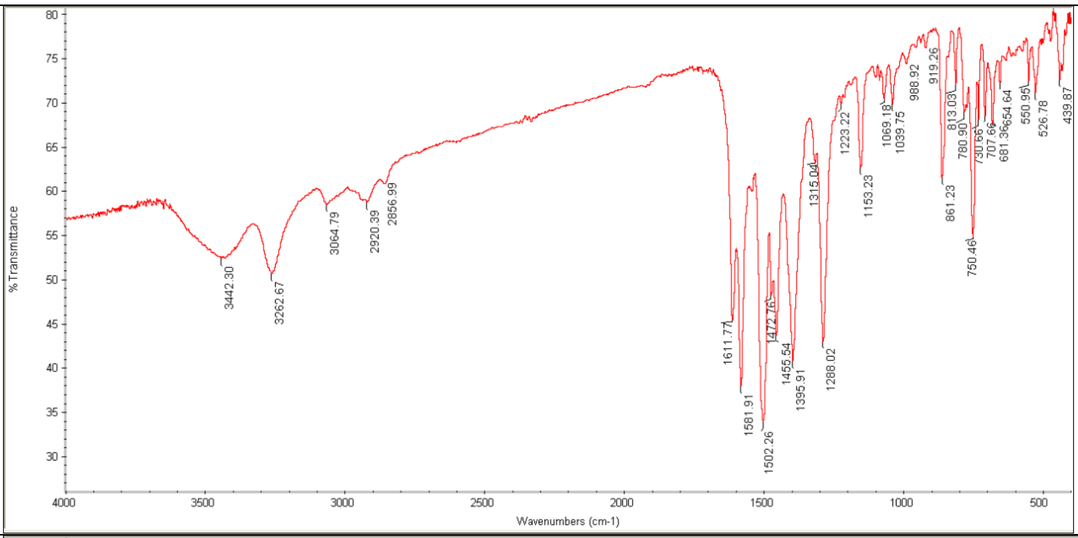

Complex **6**

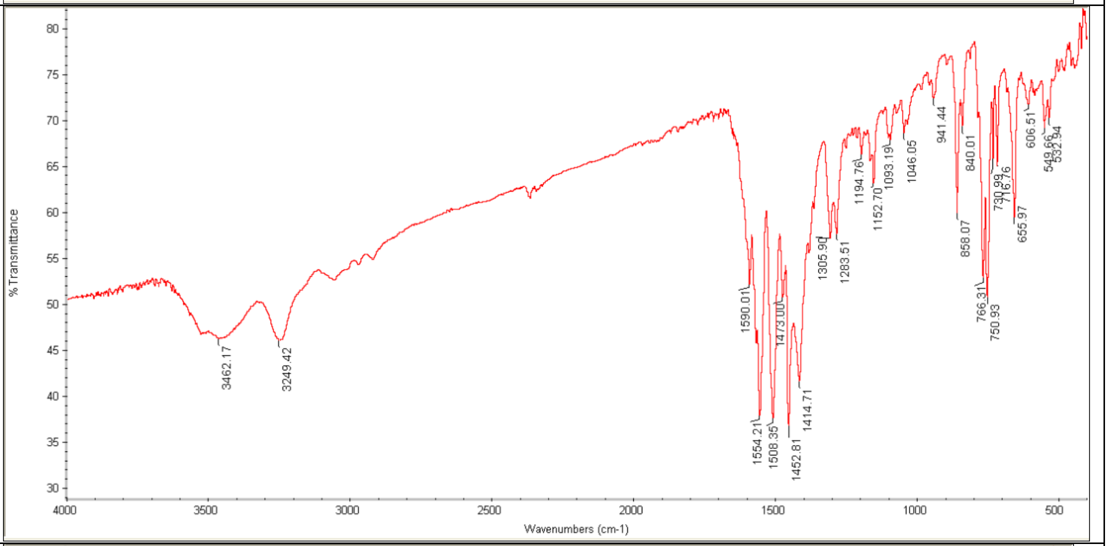

Complex **7**

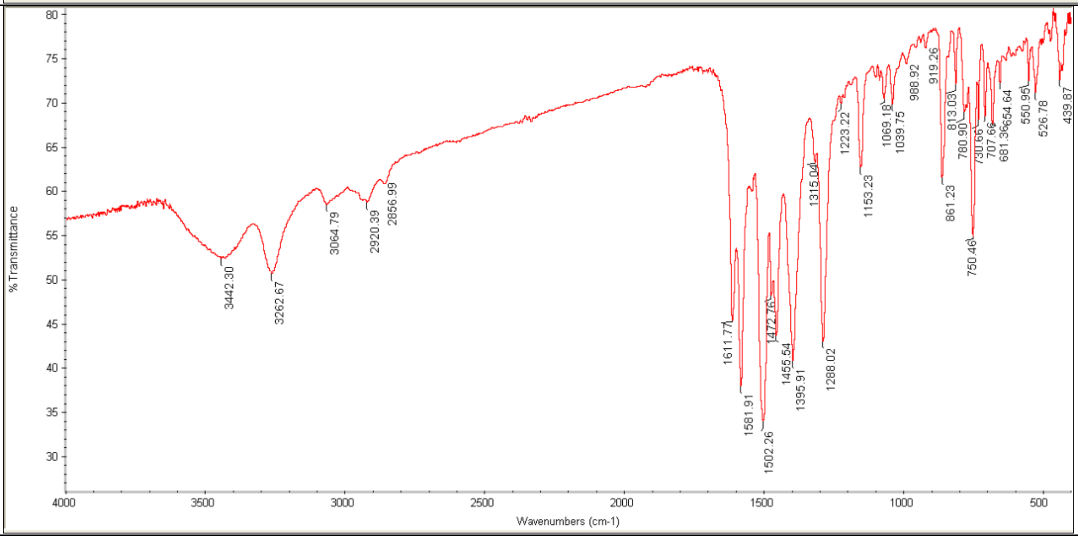

Figure S3. IR spectra (KBr pellets) of complexes **5, 6** and **7**.

Complex **8**	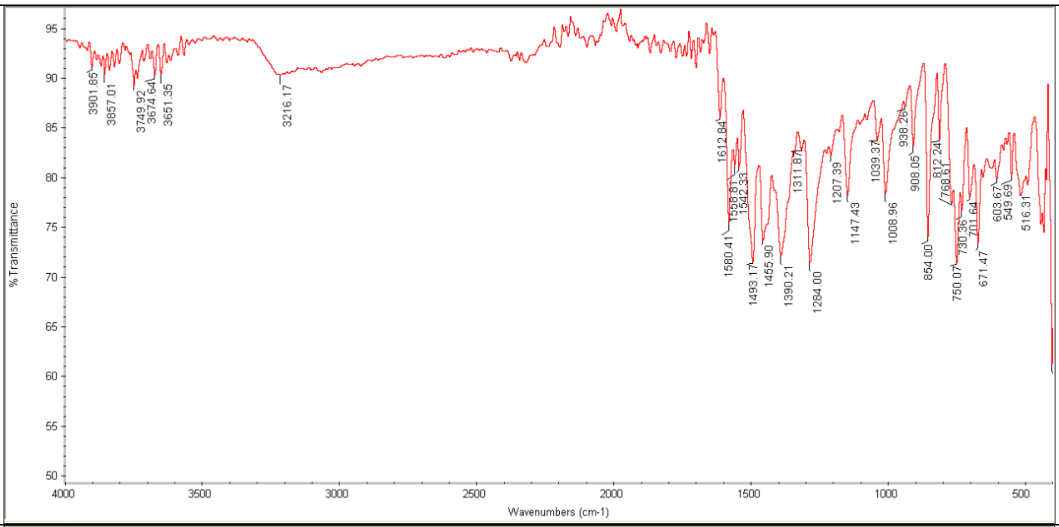
Complex **9**	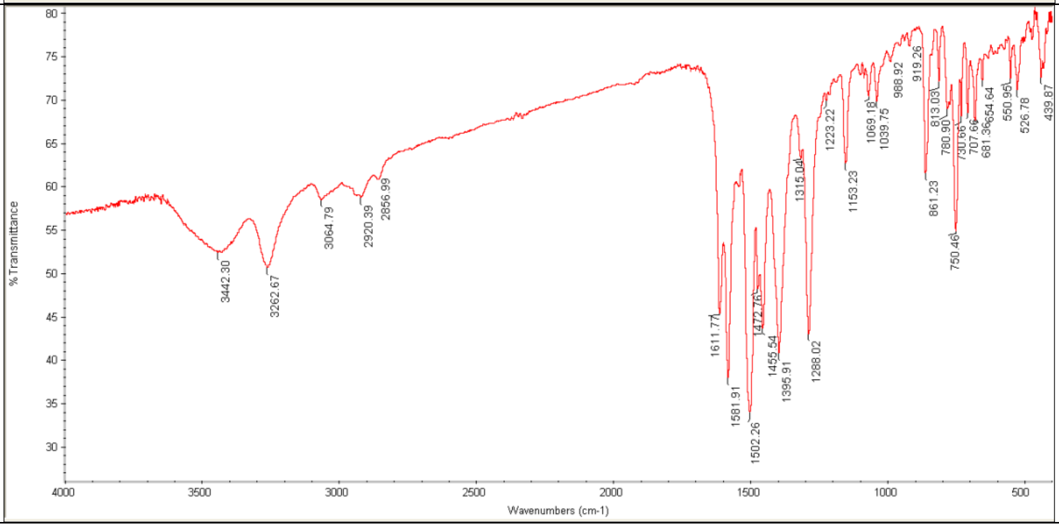

Figure S4. IR spectra (KBr pellets) of complexes **8** and **9**.

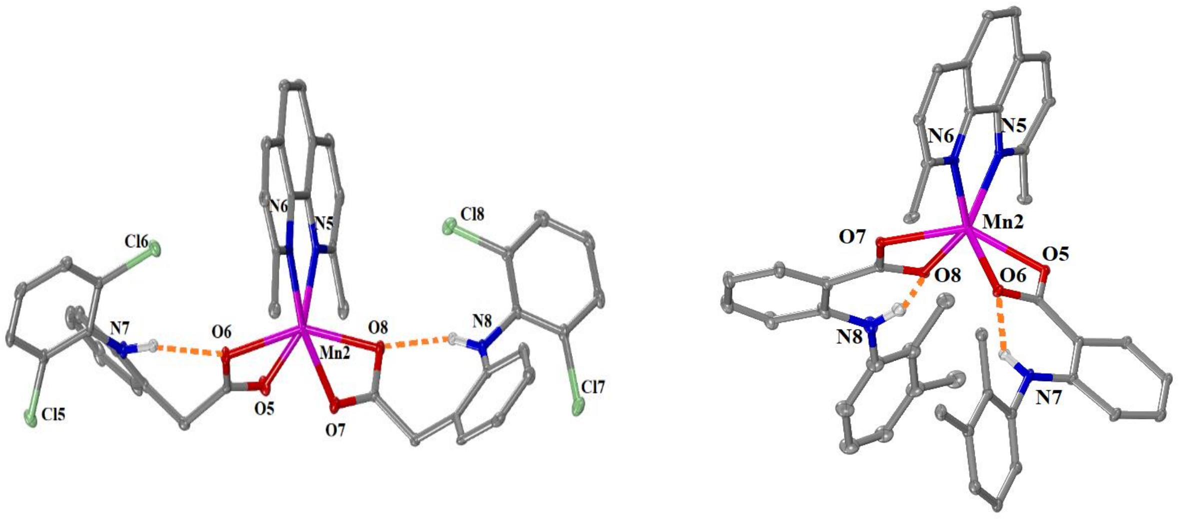

(A) (B)

Figure S5. Molecular structure of complexes (A) **5B** and (B) **6B**.

Methyl and aromatic hydrogen atoms and solvate molecules are omitted for clarity. Intraligand hydrogen bonds are given in orange dotted lines.

Figure S6. The UV−vis spectra of a CT DNA solution in buffer (containing 150 mM NaCl and 15 mM trisodium citrate at pH 7.0) recorded in the presence of increasing amounts of the complexes.

The concentration of the DNA solution is given in parentheses. The arrow shows the changes upon increasing amounts of the complex.

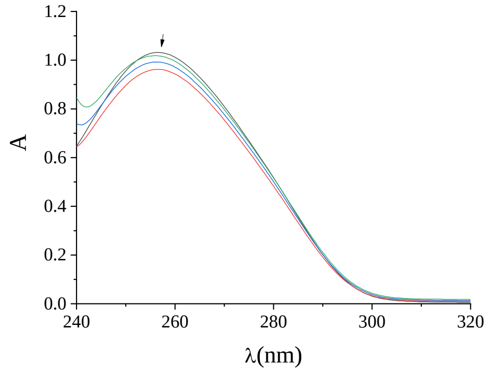

Complex **1** ([DNA] = 1.56×10^{-4} M)

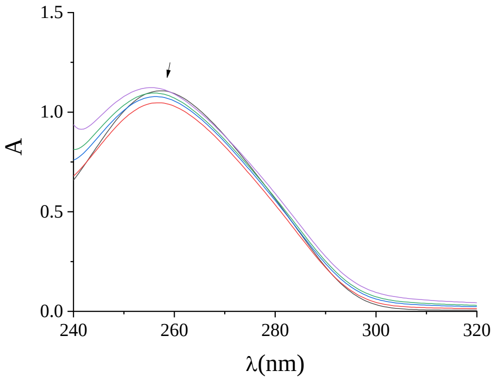

Complex **2** ([DNA] = 1.68×10^{-4} M)

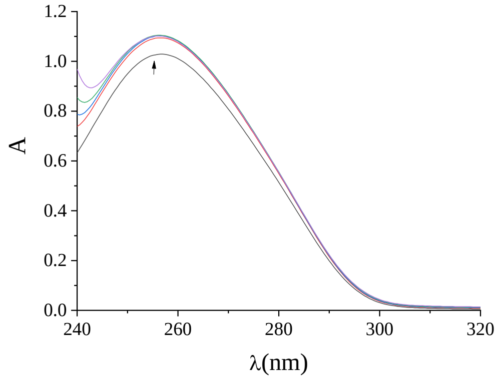

Complex **3** ([DNA] = 1.56×10^{-4} M)

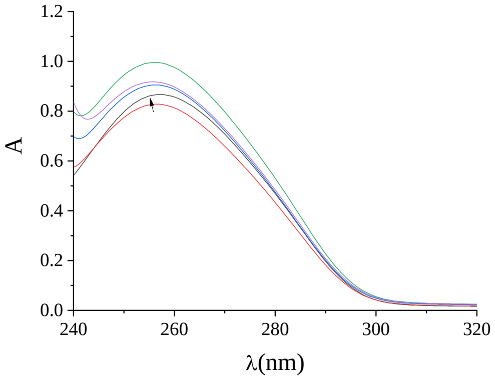

Complex **4** ([DNA] = 1.31×10^{-4} M)

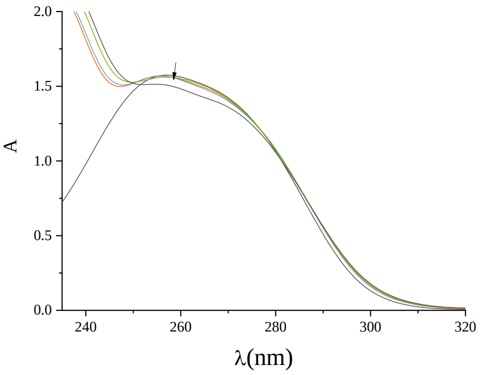

Complex **5** ([DNA] = 2.38×10^{-4} M)

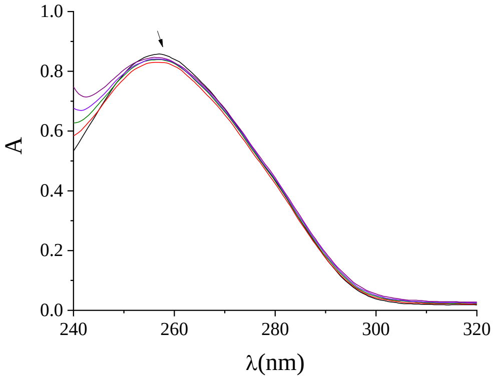

Complex **6** ([DNA] = 1.30×10^{-4} M)

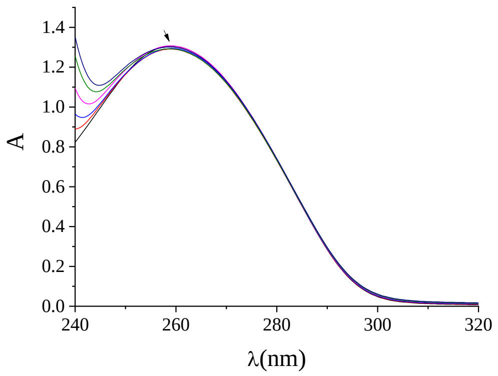

Complex **7** ([DNA] = 1.98×10^{-4} M)

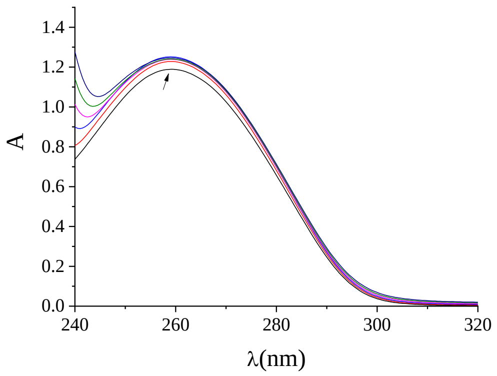

Complex **8** ([DNA] = 1.80×10^{-4} M)

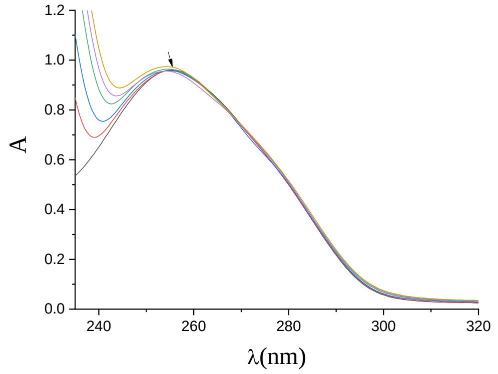

Complex **9** ([DNA] = 1.45×10^{-4} M)

Figure S7. UV−vis spectra of a DMSO solution of the complexes in the presence of increasing amounts of CT DNA.

The concentrations of the solution of the complexes are given in parentheses. The arrows show the changes upon increasing amounts of CT DNA.

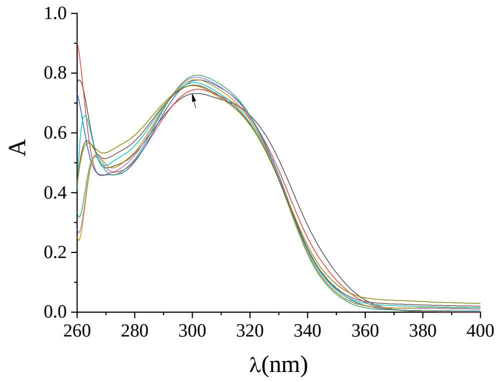

Complex **1** (50 μM)

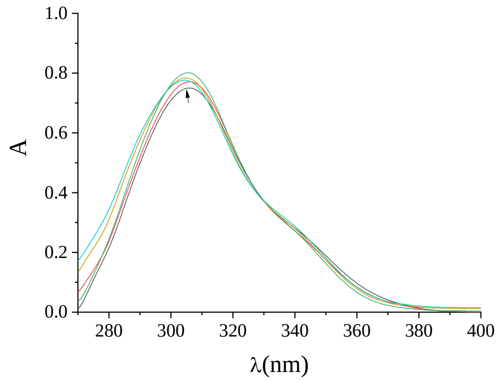

Complex **2** (20 μM)

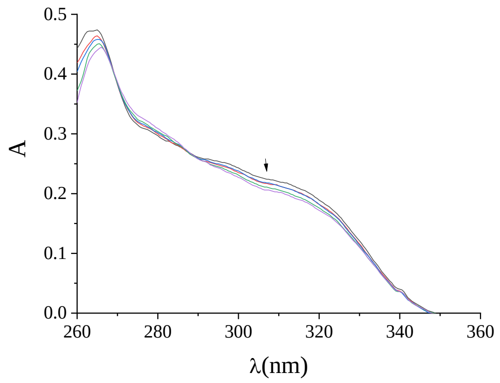

Complex **3** (10 μM)

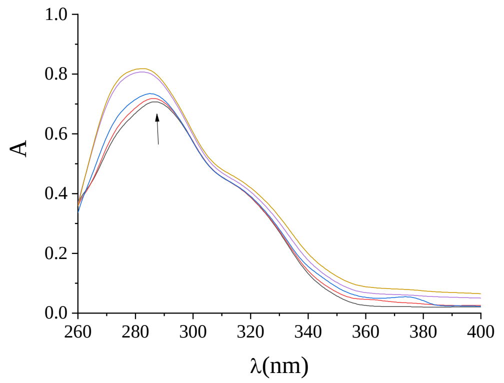

Complex **4** (20 μM)

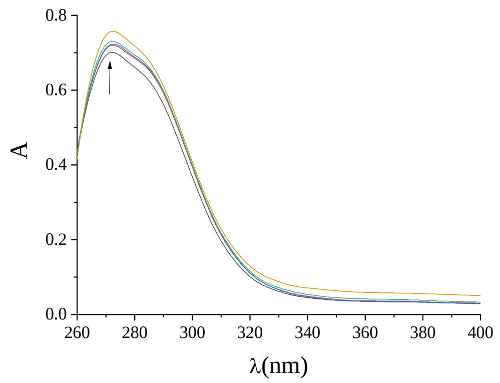

Complex **5** (10 μM)

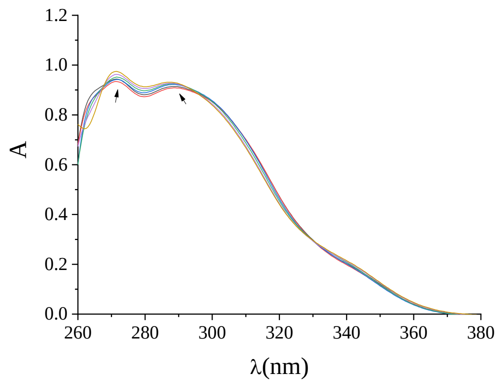

Complex **6** (20 μM)

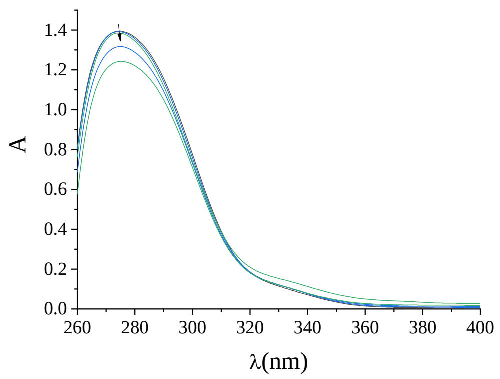

Complex **7** (10 μM)

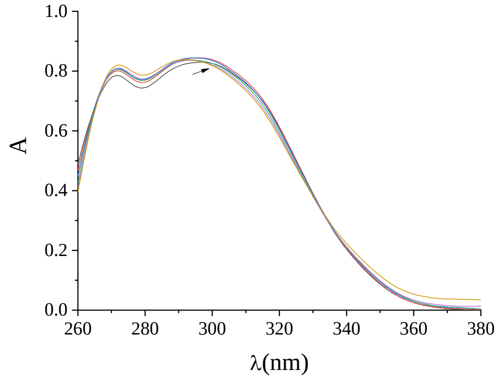

Complex **8** (20 μM)

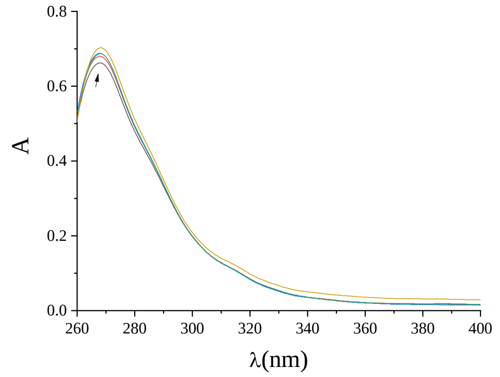

Complex **9** (10 μM)

Figure S8. Plots of $\frac{[\mathrm{DNA}]}{(\varepsilon_{\mathrm{A}} - \varepsilon_{\mathrm{f}})}$ *versus* [DNA] for the complexes.

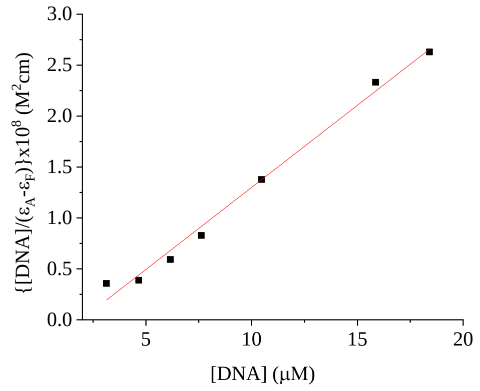

Complex **1**

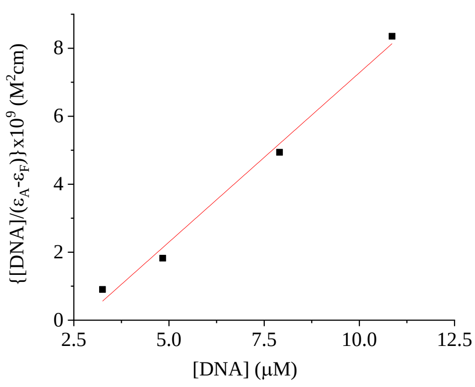

Complex **2**

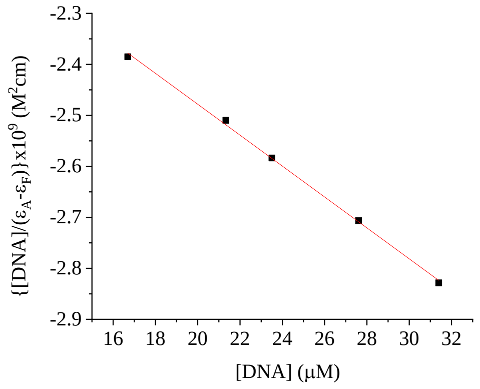

Complex **3**

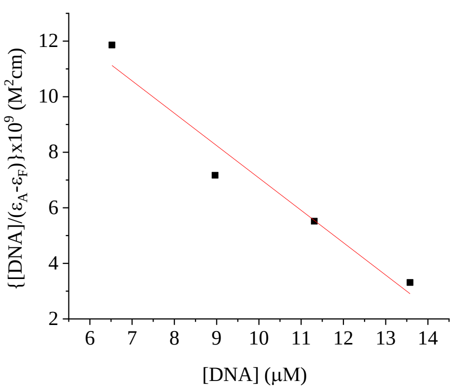

Complex **4**

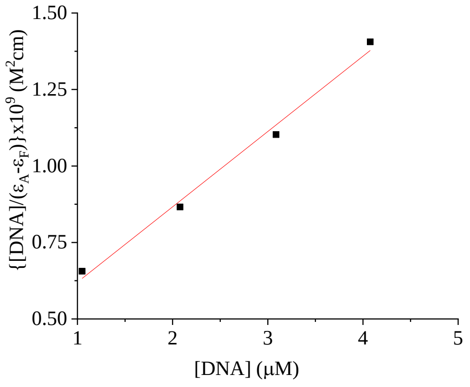

Complex **5**

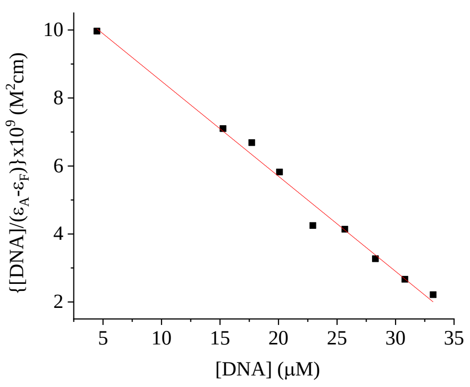

Complex **6**

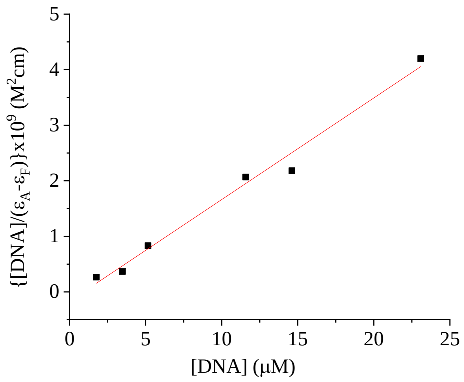

Complex **7**

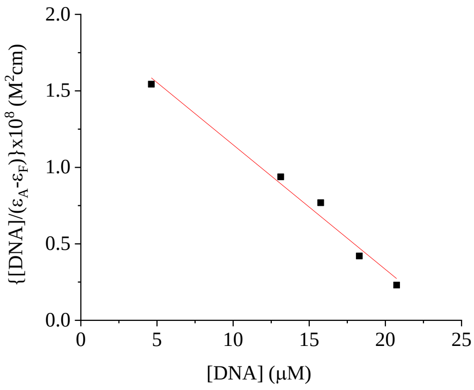

Complex **8**

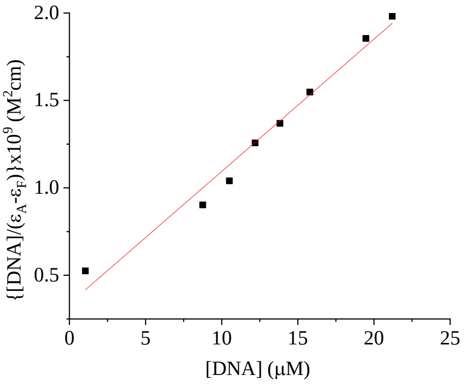

Complex **9**

Figure S9. Fluorescence emission spectra ($\lambda_{excitation}$ = 540 nm) for EB-DNA in buffer solution in the absence and presence of increasing amounts of the complexes.

Conditions: [EB] = 40 μM, [DNA] = 45 μM, buffer solution: 150 mM NaCl and 15 mM trisodium citrate at pH 7.0. The arrow shows the changes of intensity upon increasing amounts of the complex.

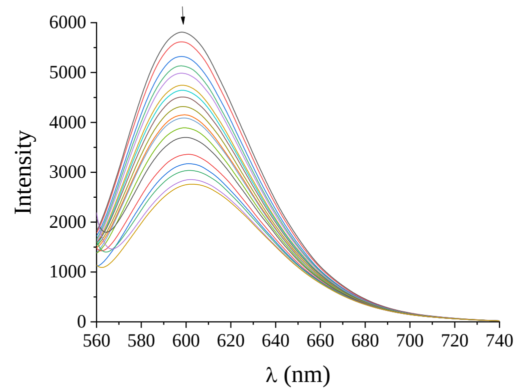

Complex **1**

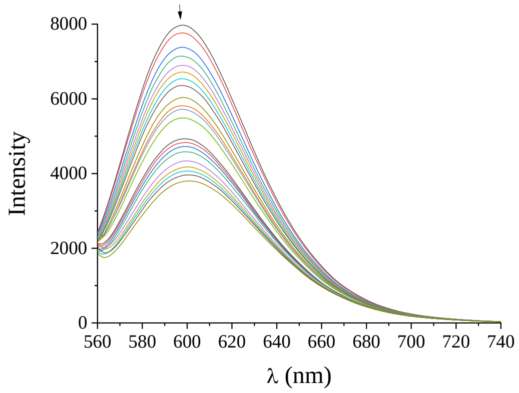

Complex **2**

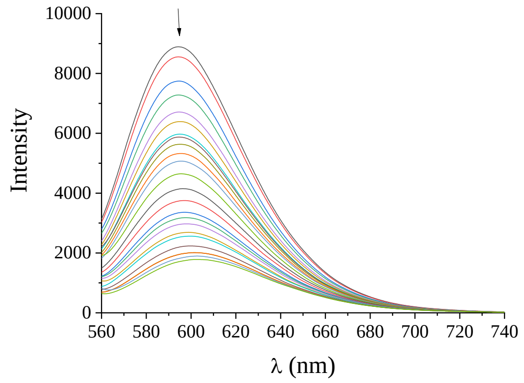

Complex **3**

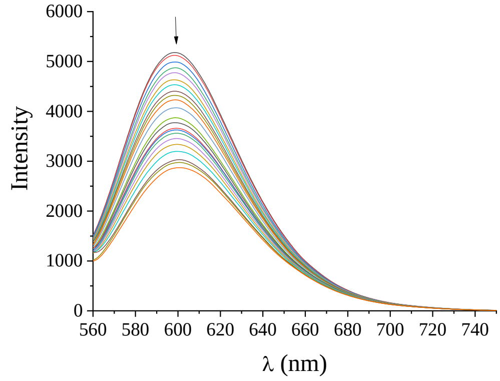

Complex **4**

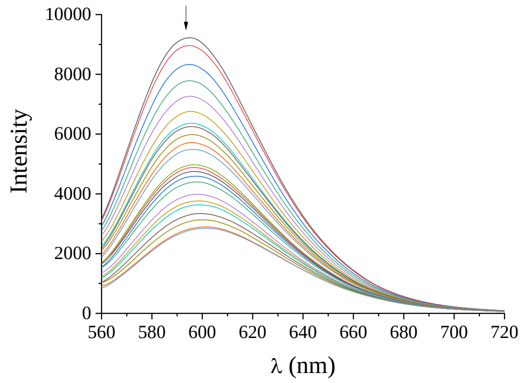

Complex **5**

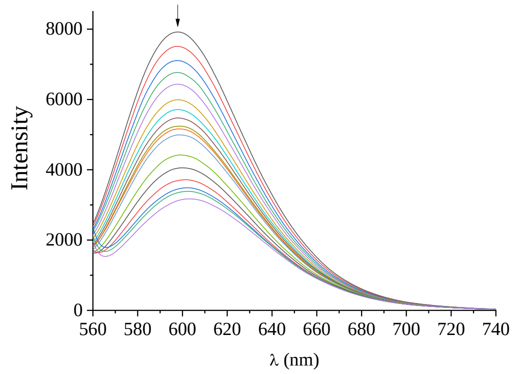

Complex **6**

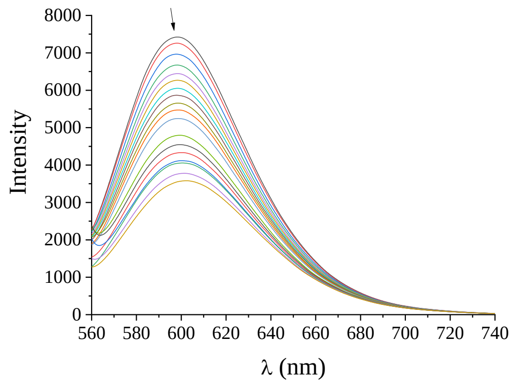

Complex **7**

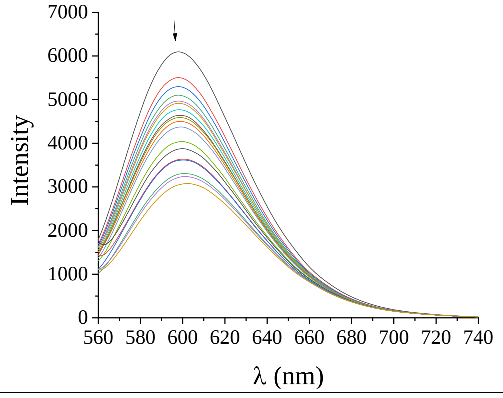

Complex **8**

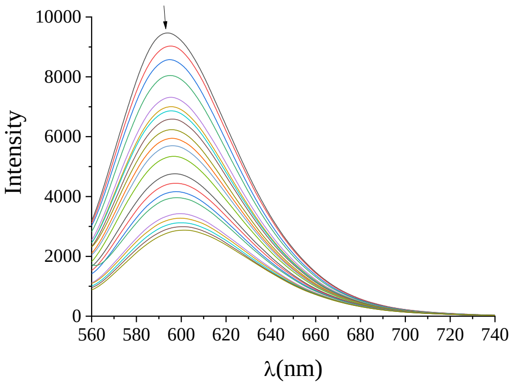

Complex **9**

Figure S10. Stern−Volmer plots of the EB−DNA quenching experiments upon addition of the complexes.

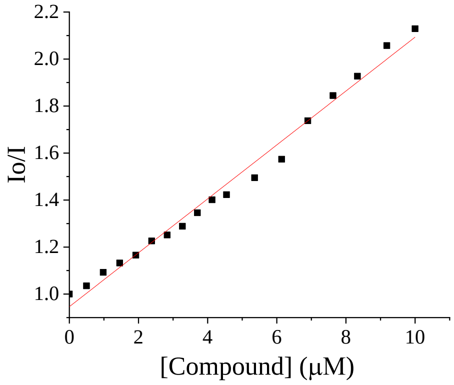

Complex **1**

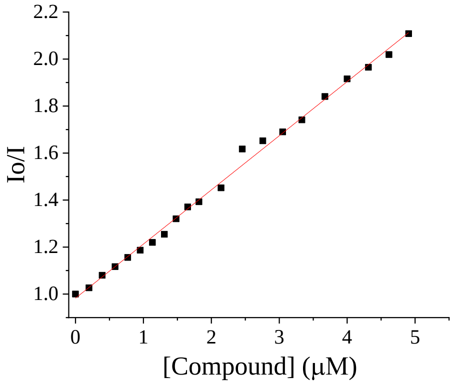

Complex **2**

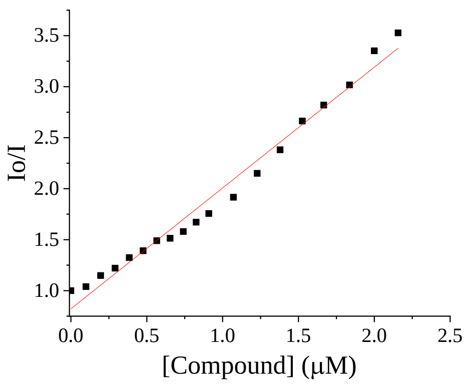

Complex **3**

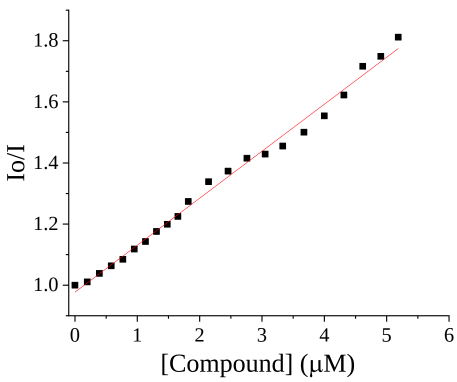

Complex **4**

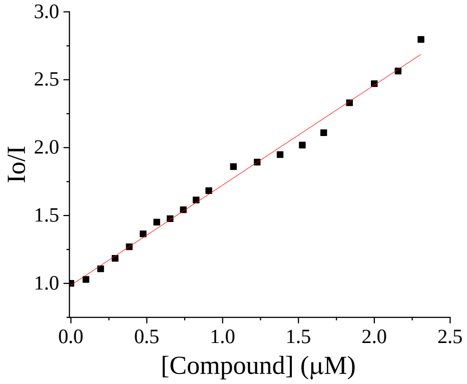

Complex **5**

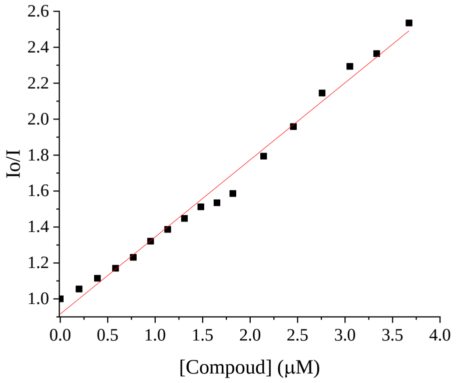

Complex **6**

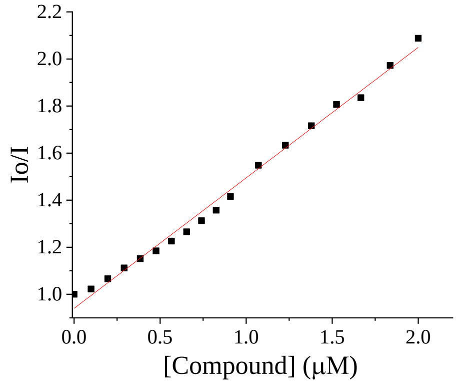

Complex **7**

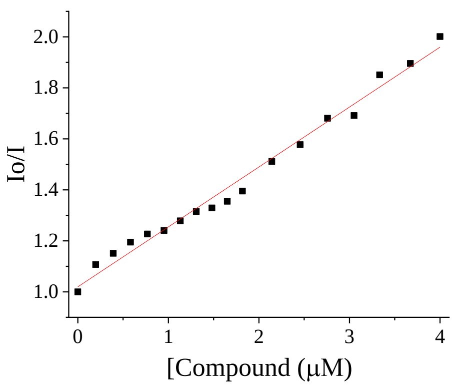

Complex **8**

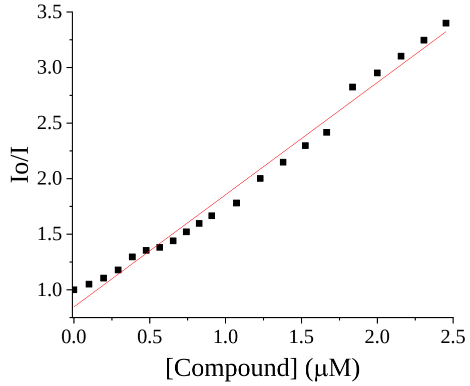

Complex **9**

Figure S11. Fluorescence emission spectra ($\lambda_{excitation}$ = 295 nm) of a buffer solution (150 mM NaCl and 15 mM trisodium citrate at pH 7.0) of HSA (3 μM) in the presence of increasing amounts of the complexes.

The arrow shows the changes of intensity upon increasing amounts of the complex.

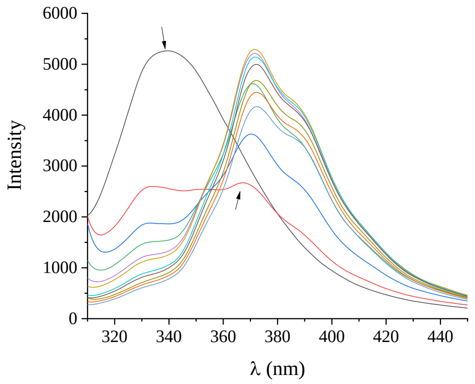

Complex **6**

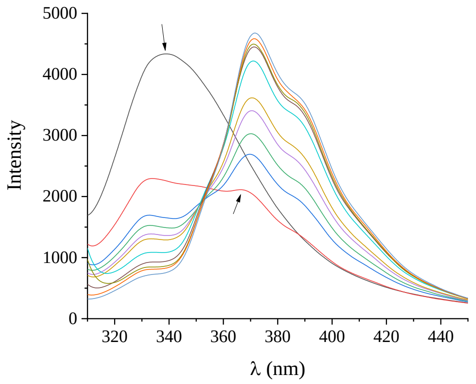

Complex **7**

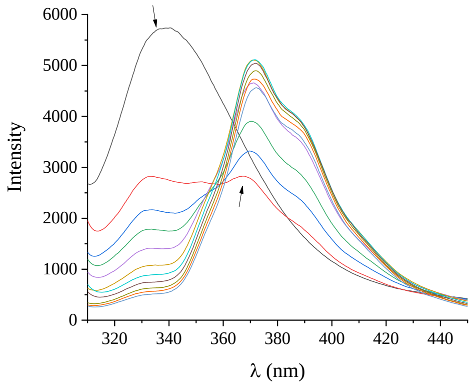

Complex **8**

Figure S12. Fluorescence emission spectra ($\lambda_{excitation}$ = 295 nm) of a buffer solution (150 mM NaCl and 15 mM trisodium citrate at pH 7.0) of BSA (3 μM) in the presence of increasing amounts of the complexes.

The arrow shows the changes of intensity upon increasing amounts of the complex.

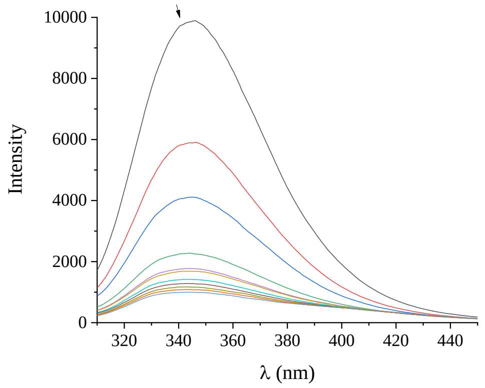

Complex **2**

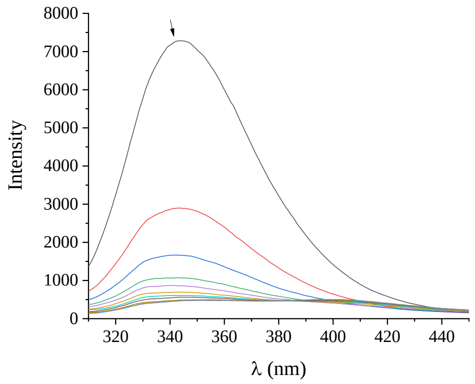

Complex **4**

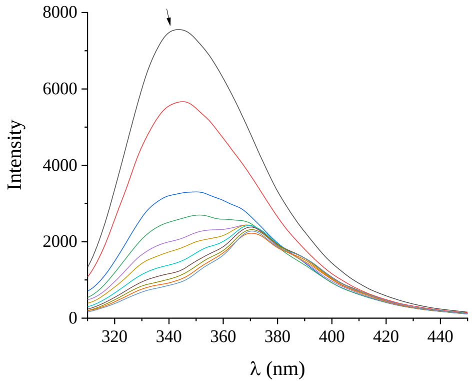

Complex **7**

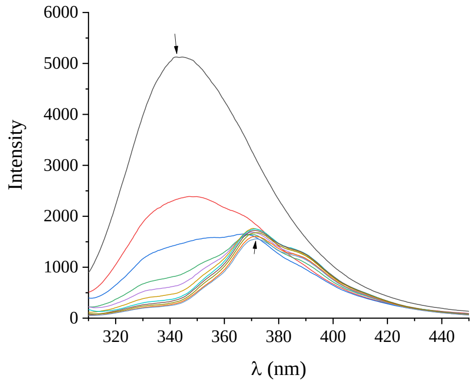

Complex **8**

Figure S13. Stern−Volmer plots of the HSA quenching experiments upon addition of the complexes.

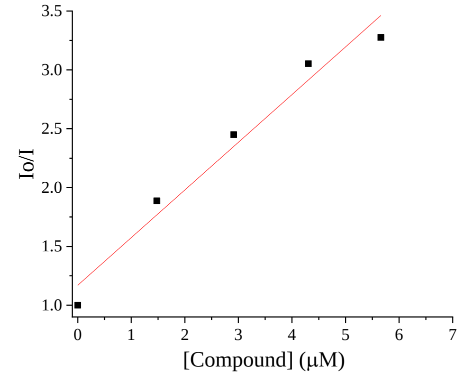	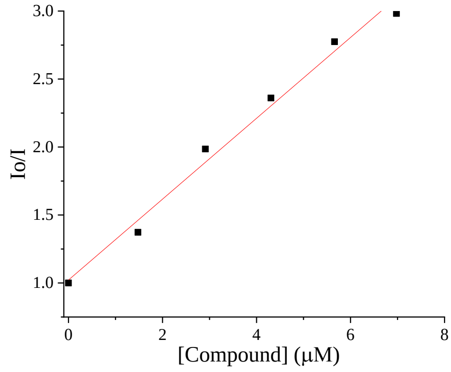
Complex **1**	Complex **2**
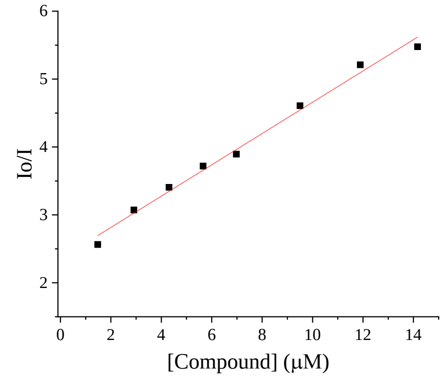	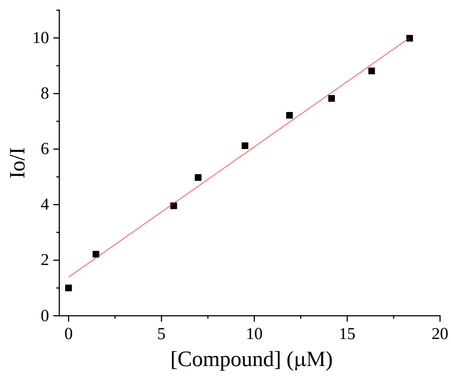
Complex **3**	Complex **4**
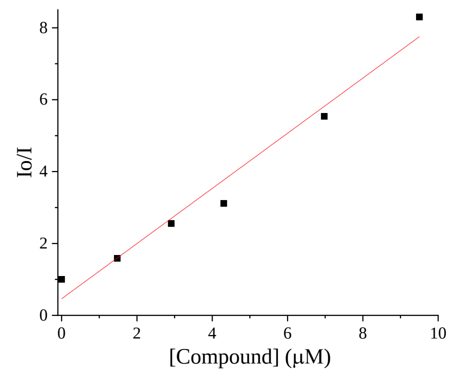	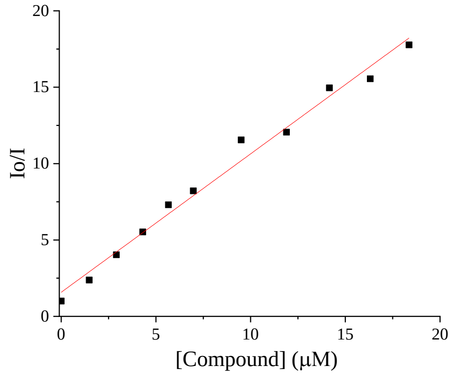
Complex **5**	Complex **6**

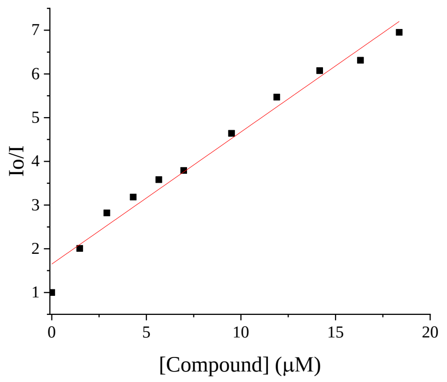

Complex **7**

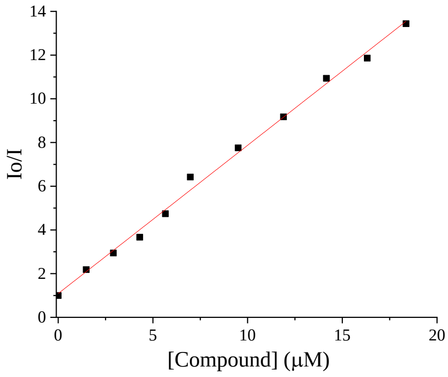

Complex **8**

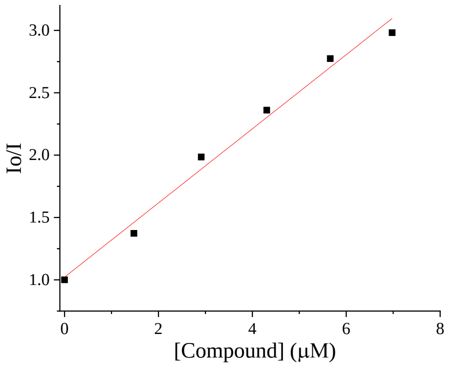

Complex **9**

Figure S14. Stern–Volmer plots of the BSA quenching experiments upon addition of the complexes.

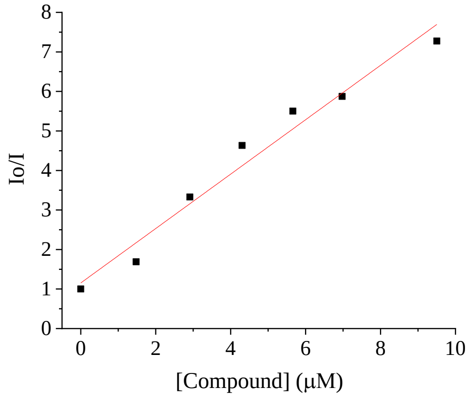

Complex **1**

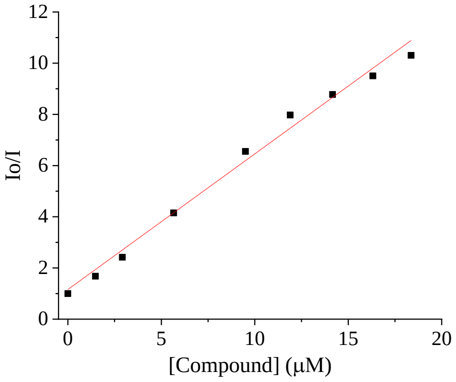

Complex **2**

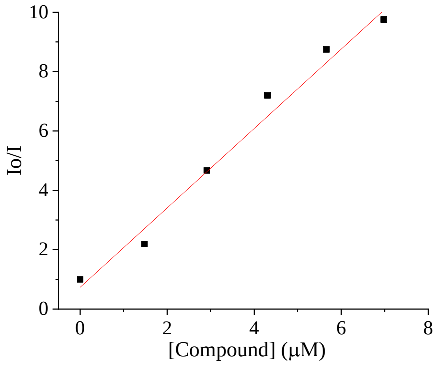

Complex **3**

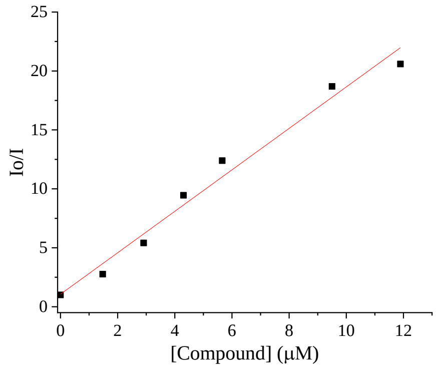

Complex **4**

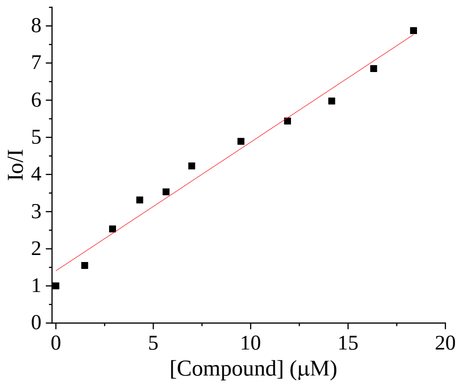

Complex **5**

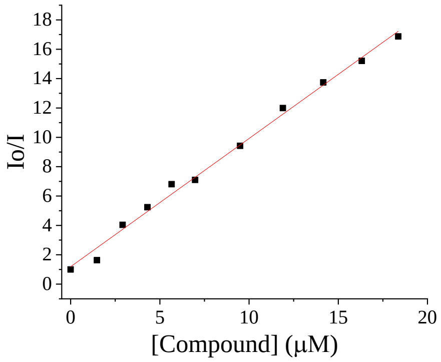

Complex **6**

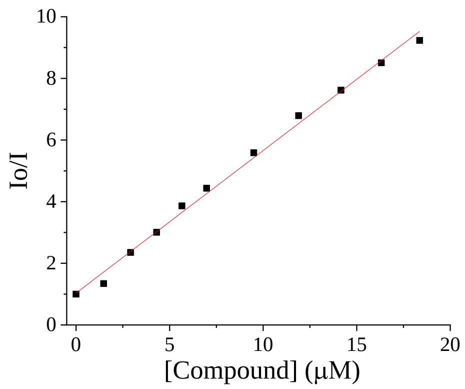

Complex **7**

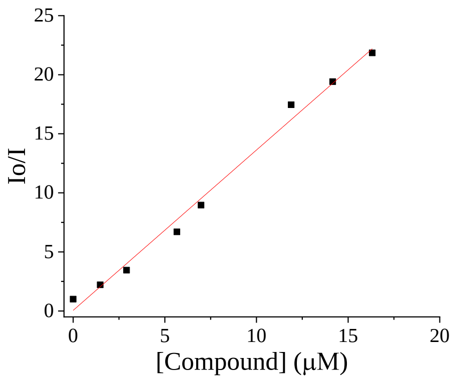

Complex **8**

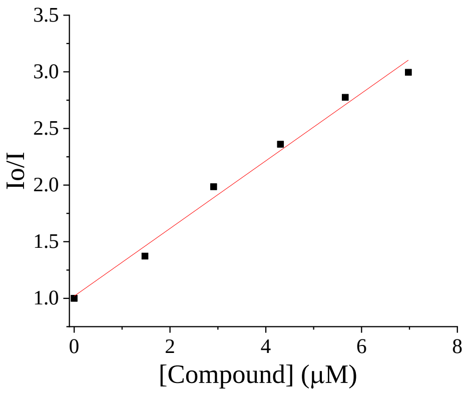

Complex **9**

Figure S15. Scatchard plots of the HSA quenching experiments upon addition of the complexes.

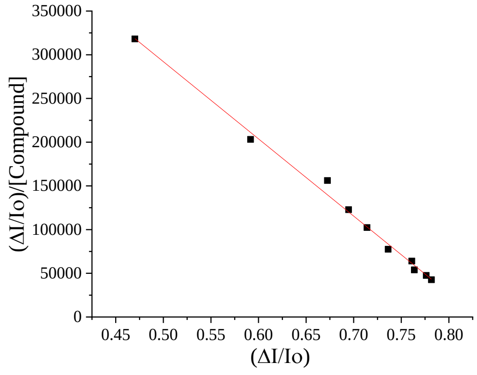

Complex **1**

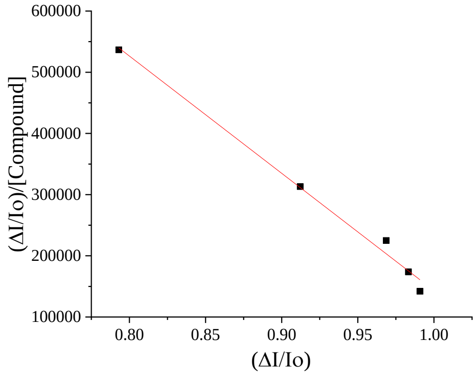

Complex **2**

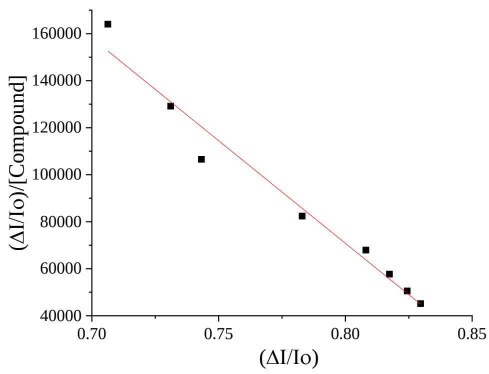

Complex **3**

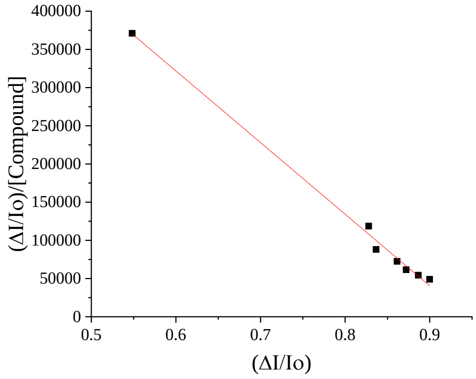

Complex **4**

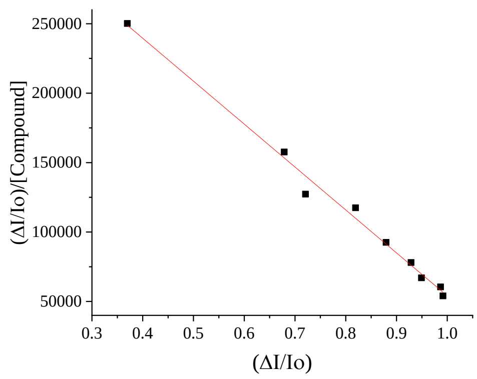

Complex **5**

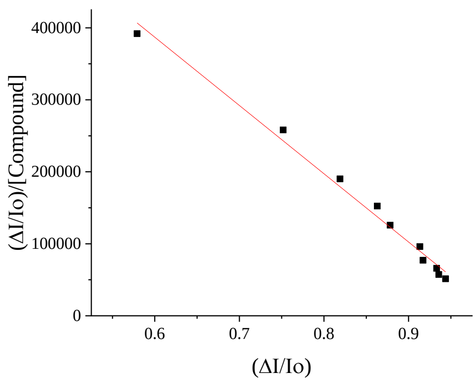

Complex **6**

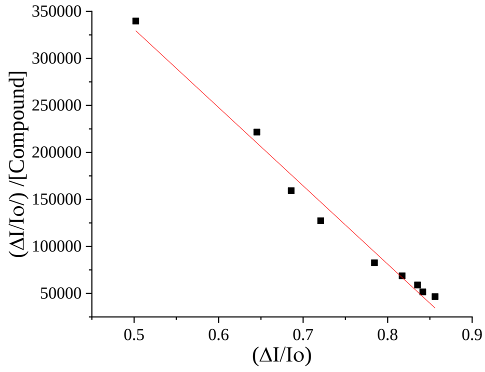	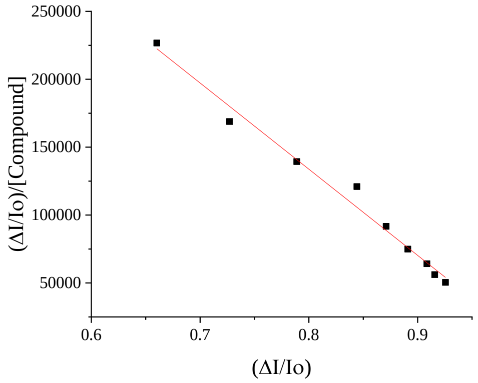
Complex **7**	Complex **8**
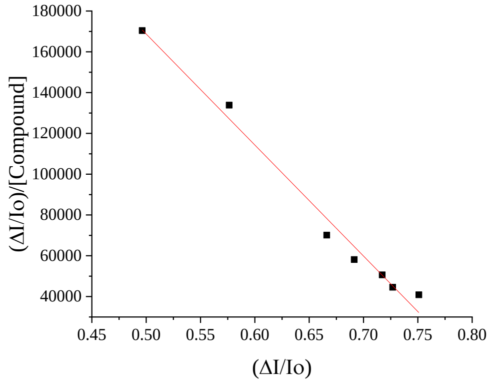	
Complex **9**	

Figure S16. Scatchard plots of the BSA quenching experiments upon addition of the complexes.

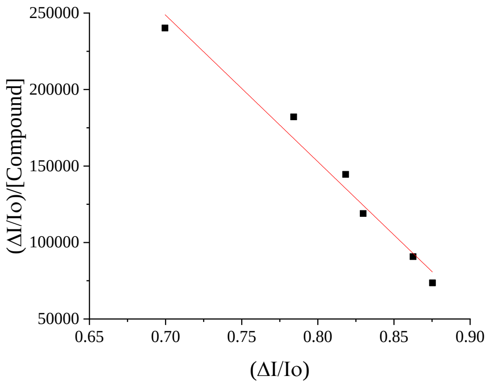

Complex **1**

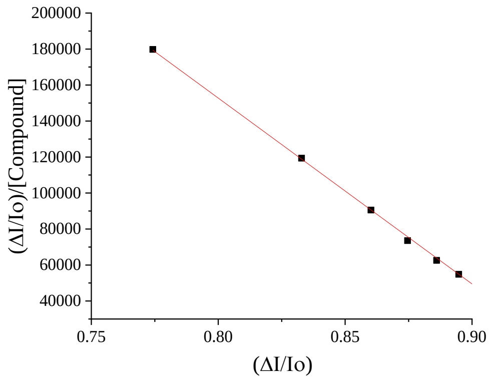

Complex **2**

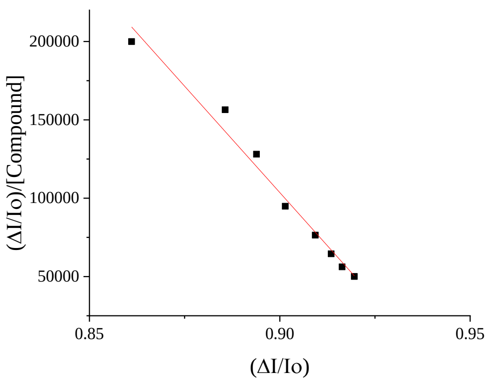

Complex **3**

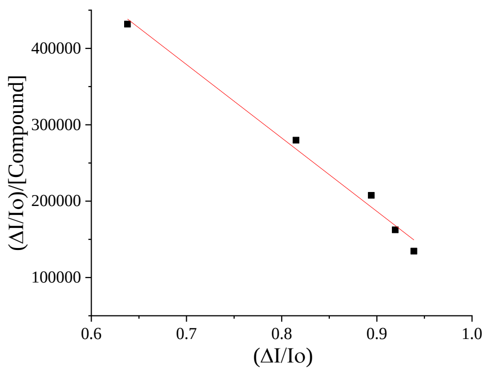

Complex **4**

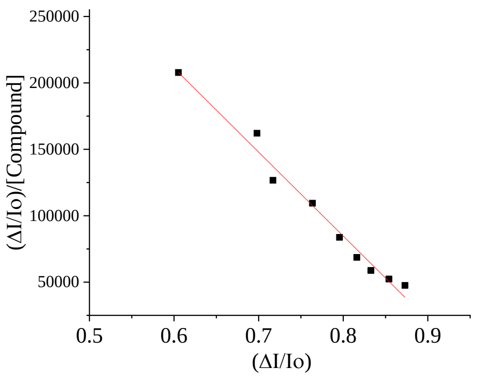

Complex **5**

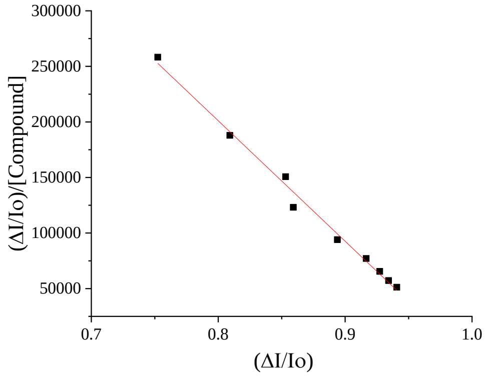

Complex **6**

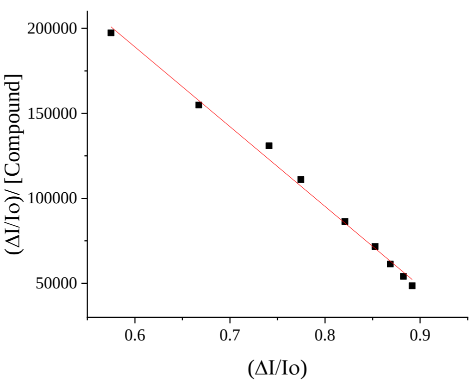

Complex **7**

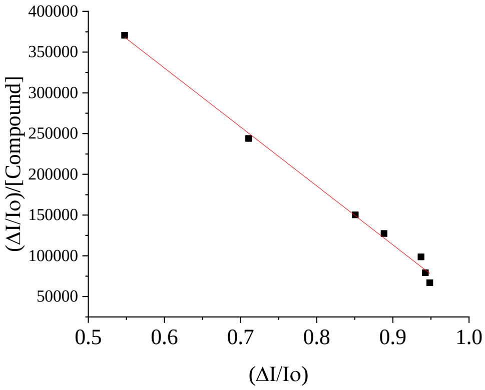

Complex **8**

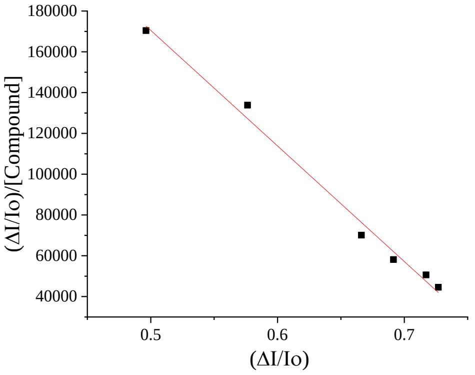

Complex **9**
